# Computed tomography reveals the endocranial anatomy of Crocodylia: Implications for phylogenetic relationships and ecomorphological convergence across Crocodylomorpha

**DOI:** 10.1111/joa.70182

**Published:** 2026-06-26

**Authors:** Paul M. J. Burke, Philip D. Mannion

**Affiliations:** ^1^ Department of Earth Sciences University College London London UK; ^2^ Department of Paleobiology Naturhistoriska Riksmuseet Stockholm Sweden

**Keywords:** computed tomography, Crocodylia, ecomorphological variation, endocranial anatomy, longirostrine

## Abstract

Living crocodylians (alligators, caimans, gavials and ‘true’ crocodiles) are the remnants of a broader clade, Crocodylomorpha, that displays high ecomorphological diversity, particularly in skull shape. However, there are several instances of ecomorphological convergence between distant lineages, especially pertaining to the independent acquisition of a long, slender, longirostrine snout. In some instances, it is difficult to determine whether extinct species belong to one longirostrine lineage or another, with this problem especially prevalent with regards to the gavialoid crocodylian clade. The recent application of computed tomography (CT) scanning to the skulls of vertebrate species has revealed a plethora of previously hidden information on their endocranial anatomy. This in turn has the potential to shed new light on a group's evolutionary interrelationships, with endocranial data potentially helping to distinguish between shared phylogenetic ancestry of an anatomical feature and ecomorphological convergence. Although CT scans of skulls of several crocodylian species have been evaluated, previous studies have focused on describing the endocranial anatomy of an individual species or assessing intraclade variation of a specific morphological feature. Here, we evaluate the endocranial anatomy of Crocodylia based on a combination of published and newly presented CT‐scan data for 43 extant and extinct species, including representatives from the three major subclades, Alligatoroidea (13 species), Crocodyloidea (14 species), and Gavialoidea (16 species), alongside four non‐crocodylian eusuchians. Alligatoroids possess a sigmoidal encephalic endocast, an endosseous labyrinth wherein the area of the anterior semicircular canal is more than three times that of its posterior counterpart, as well as snout sinuses lateral and ventral to the nasal cavity. Crocodyloids have dorsoventrally tall pharyngotympanic tubes, antorbital and postvestibular sinuses, and a cerebrum that has its greatest transverse width at its midpoint. Gavialoids have a relatively ‘simple’ endocranial morphology, with a straight encephalic endocast, and reduced or absent snout sinuses. Several extinct gavialoids are also characterised by depressions on the internal surface of the prefrontal bones, which have been previously hypothesised to be osteological correlates for salt glands; however, similar depressions in some extant species of *Crocodylus* appear to be associated with airspace instead. We identify several differences between the endocranial anatomy of phylogenetically distant longirostrine crocodylomorphs: by contrast with thalattosuchians, crocodylians lack a confluent dorsal dural venous sinus and orbital arteries, whereas they possess an intertympanic sinus and nasolacrimal ducts. Our study demonstrates the viability of endocranial data for identifying phylogenetically informative morphological features in crocodylomorphs more broadly.

## Graphical abstract

The evaluation of the endocranial anatomy of 43 crocodylian species reveals several differences, including variation in the curvature of the encephalic endocast, the size of the endosseous labyrinth and paratympanic sinuses, and the presence of additional snout sinuses. Differences between phylogenetically distant longirostrine crocodylomorphs further demonstrate the value of endocranial data for identifying informative morphological features.

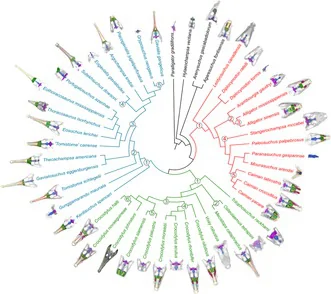

## 1 INTRODUCTION

Crocodylia is a clade that today consists of semi‐aquatic, ambush predators and piscivores that inhabit both freshwater and estuarine environments (Grigg & Kirshner, 2015). They are broadly restricted to the subtropical latitudinal belt, with 27 extant species currently recognised (Murray et al., 2019; Shirley et al., 2018), comprising alligators, caimans, ‘true’ crocodiles and gavials (Grigg & Kirshner, 2015). From taxa first appearing in the Late Cretaceous, approximately 100 million years ago (Darlim et al., 2022; Mateus et al., 2019), more than 140 species are currently recognised as members of Crocodylia (Brochu, 2003; Rio & Mannion, 2021). The phylogenetic interrelationships of Crocodylia have been debated for decades, with conflicting results from analyses based on molecular (Densmore & Owen, 1989) versus morphological (Brochu, 1997) data. The recovery of *Gavialis gangeticus* as the closest living relative of *Tomistoma schlegelii*, forming the gavialoid clade Gavialidae, has been resolved in recent morphological analyses, removing the main discrepancy with molecular analyses (see Rio & Mannion, 2021; Ristevski et al., 2022). However, a temporal incongruence remains, in which several stratigraphically early putative gavialoid species are typically recovered within Gavialidae (Burke et al., 2025; Rio & Mannion, 2021), despite molecular divergence date estimates placing the origin of this clade much more recently (Oaks, 2011; Pan et al., 2021). These putative gavialoids, commonly referred to as ‘thoracosaurs’, inhabited coastal to marine environments and share the presence of a long and narrow rostrum (=‘longirostrine’ skull morphology) with gavialids, but it remains possible that longirostry is convergent between these lineages and that thoracosaurs lie outside of Gavialidae or Crocodylia altogether (see Boerman et al., in review; Brochu, 2004; Burke et al., 2025; Darlim et al., 2022; Groh et al., 2020; Lee & Yates, 2018; Rio & Mannion, 2021; Vélez‐Rosado et al., 2025; Zamora‐Vega et al., 2025).

Within Crocodylia, longirostry also characterises the extant ‘true’ crocodiles, *Mecistops cataphractus* (Gray, 1844) and *Crocodylus johnstoni* (Krefft, 1873), in addition to the aforementioned gavialoids. It is clear that longirostry has evolved independently several times throughout the evolutionary history of the larger clade to which crocodylians belong, Crocodylomorpha, including members of the fully marine clade Thalattosuchia, as well as in the semi‐to‐fully aquatic species of Tethysuchia (e.g. Ballel et al., 2019; Brochu, 2001; Groh et al., 2020). As is the case in crocodylians, the phylogenetic positions of the longirostrine groups within Crocodylomorpha are debated, with ecomorphological convergence a likely factor in obfuscating interrelationships (Groh et al., 2020; Wilberg, 2015).

Given that these problems remain despite increased sampling of taxa and morphological variation in crocodylomorph phylogenetic datasets (e.g. Groh et al., 2020; Rio & Mannion, 2021; Young et al., 2024), we need to turn to other sources of information. One possible avenue is the internal cranial anatomy of these species, which has started to be documented in a growing number of studies via computed tomography (CT) scanning. The use of CT data is a relatively recent expansion in palaeontology, and its utilisation in fossil taxa has helped further our knowledge of these extinct organisms, with the first applications to crocodylians and their extinct relatives implemented by Willis et al. (1995), Rowe et al. (1999) and Tykoski et al. (2002). Whereas early studies to utilise CT data were mainly focused on extant species, especially *Alligator mississippiensis* (Dufeau & Witmer, 2015; Gold et al., 2014; Lessner & Holliday, 2022; Witmer et al., 2008; Witmer & Ridgely, 2008), there has been an increase in applications to extinct taxa in the last decade (Barrios et al., 2023). Many of these studies have focused on early‐diverging crocodylomorphs, including the marine thalattosuchians (Brusatte et al., 2016; Herrera et al., 2018; Pierce et al., 2017; Schwab et al., 2021; Wilberg et al., 2022) and their fully terrestrial relatives (Leardi et al., 2020; Melstrom et al., 2022; Ruebenstahl et al., 2022), as well as notosuchians (Sereno & Larsson, 2009; Kley et al., 2010; Sertich & O'Connor, 2014; Fonseca et al., 2020; Pochat‐Cottilloux et al., 2022; Dumont Jr. et al., 2022; Pochat‐Cottilloux et al., 2023), and eusuchian taxa outside of the crocodylian radiation (Blanco et al., 2015; Holliday & Gardner, 2012; Puértolas‐Pascual et al., 2022; Puértolas‐Pascual et al., 2023; Serrano‐Martínez et al., 2019; Serrano‐Martínez et al., 2021).

A small number of studies have used CT data to evaluate interspecific variation of individual features of the crocodylomorph skull. This includes the morphology of individual elements that form the braincase (Kuzmin et al., 2021), the size and shape of the Eustachian canals (Gold et al., 2014), the shape and robusticity of the endosseous labyrinth (Pochat‐Cottilloux et al., 2025; Ristevski, 2022; Schwab et al., 2020), and ontogenetic changes in the shape of the intertympanic sinus (Perrichon et al., 2023). Collectively, these studies, amongst others (e.g. Boerman et al., in review; Burke & Mannion, 2023; Perrichon et al., 2024), suggest that a broader survey of endocranial anatomy could shed light on both the phylogenetic relationships of crocodylomorphs, as well as ecomorphological convergence between distant lineages.

Here, we evaluate endocranial anatomy across Crocodylia, detailing and illustrating variation in morphological features of the encephalic endocast, endosseous labyrinth, paratympanic sinus, nasopharyngeal ducts, as well as the nasal cavity and associated features of 43 extant and extinct species, representing all three major subclades (Alligatoroidea, Crocodyloidea and Gavialoidea). We utilise these data to better understand how endocranial anatomy varies within Crocodylia in a phylogenetic context, in addition to identifying key ecomorphological similarities and differences with other extinct crocodylomorph lineages, especially those characterised by longirostry.

*Institutional abbreviations*. DSM, Dinosaur State Museum, Connecticut, USA; DVZ, Department of Vertebrate Zoology of Saint Petersburg State University, Saint Petersburg, Russia; FLMNH, Florida Museum of Natural History, Florida, USA; IRSNB, Institut Royal des Sciences Naturalles de Belgique, Brussels, Belgium; KIT, Karlsruhe Institute of Technology, Karlsruhe, Germany; KME, Krahuletz Museum, Eggenburg, Austria; LO, Lund University, Lund, Sweden; MGL, Musée d'Histoire Naturelle de Lille, Lille, France; MHNL, Musée d'Histoire Naturelle de Lyon, Lyon, France; MLP, Museo de La Plata, Buenos Aires, Argentina; MNHN, Muséum National d'Histoire Naturelle, Paris, France; MOU, Museum of Osaka University, Osaka, Japan; MPZ, Museo de Ciencias Naturales de la Universidad de Zaragoza, Zaragoza, Spain; MUPA, Museo Paleontológico de Castilla‐La Mancha, Cuenca, Spain; MZS, Musée Zoologique de Strasbourg, Strasbourg, France; NMB, National Museum of the Bahamas, Nassau, Bahamas; NHMUK, Natural History Museum, London, United Kingdom; OUVC, Ohio University Vertebrate Collection, Athens, Ohio, USA; PIN, Borissiak Paleontological Institute, Russian Academy of Sciences, Moscow, Russia; QM, Queensland Museum, Brisbane, Queensland, Australia; SMM, Science Museum of Minnesota, Minnesota, USA; SMNK, Staatliches Museum für Naturkunde Karlsruhe, Karlsruhe, Germany; SMNS, Staatliches Museum für Naturkunde Stuttgart, Stuttgart, Germany; STUS, Sala de las Tortugas de la Universidad de Salamanca, Salamanca, Spain; TMM, Texas Memorial Museum, Austin, Texas, USA; UCBL, Université Claude Bernard Lyon 1, Lyon, France; UMMZ, University of Michigan Museum of Zoology, Ann Arbor, Michigan, USA.

## 2 MATERIALS AND METHODS

In total, CT data were acquired for 43 species of Crocodylia, including both extant and extinct taxa (Table 1), as well as four non‐crocodylian eusuchians: *Agaresuchus fontisensis*, *Arenysuchus gascabadiolorum, Hylaeochampsa vectiana* and *Paralligator gradilifrons*. Table 1 lists all species evaluated in this study, along with information on their spatiotemporal distribution and the specimens (and their sources) that form the basis for the CT‐scan reconstructions.

**TABLE 1:** List of specimens included in this study.

Clade	Species	Specimen number	Spatiotemporal distribution	Preservation	Source
Non‐crocodylian eusuchian	*Agaresuchus fontisensis* ^a^	MUPA‐HUE‐02702	Campanian–Maastrichtian (Late Cretaceous), Spain	Cranium	Serrano‐Martínez et al. (2021)
	*Arenysuchus gascabadiolorum* ^a^	MPZ 2011/184	Maastrichtian (Late Cretaceous), Spain	Partial cranium (missing right side)	Puértolas‐Pascual et al. (2022)
	*Hylaeochampsa vectiana* ^a^	NHMUK R177	Barremian (Early Cretaceous), United Kingdom	Partial skull	Nikon Metrology XTH 225 ST, NHMUK
	*Paralligator gradilifrons* ^a^	PIN 554–1/1	Early–Late Cretaceous, Mongolia	Cranium	Kuzmin et al. (2025)
Alligatoroidea	*Alligator mississippiensis*	OUVC 9761	Extant, USA	Cranium	MorphoSourcedoi:10.17602/M2/M71998
	*Alligator sinensis*	NHMW‐Zoo‐HS‐37966	Extant, China	Cranium	Donzé et al. (2026)
	*Arambourgia gaudryi* ^a^	MNHN QU17155	Middle–Late Eocene, France	Cranium	Conedera et al. (2023)
	*Caiman crocodilus*	UMMZ 128024	Extant, Central and South America	Cranium	MorphoSource ark:/87602/m4/M83806
	*Caiman latirostris*	UMMZ 155287	Extant, South America	Cranium	MorphoSource ark:/87602/m4/M100361
	*Caiman yacare*	UMMZ 155289	Extant, South America	Cranium	MorphoSource ark:/87602/m4/M100363
	*Diplocynodon ratelii* ^a^	MHNL‐LA86	Early Miocene, Czechia	Cranium	Donzé et al. (2026)
	*Diplocynodon tormis* ^a^	STUS‐344	Middle Eocene, Spain	Cranium	Serrano‐Martínez et al. (2021)
	*Leidyosuchus canadensis* ^a^	TMP 1986.221.1	Late Cretaceous, Canada	Cranium	Donzé et al. (2026)
	*Mourasuchus arendsi* ^a^	MLP 73‐IV‐15‐9	Late Miocene, Argentina	Partial skull table	Bona et al. (2013)
	*Paleosuchus palpebrosus*	L‐ERS:M:2020–004	Extant, South America	Full body	MorphoSource ark:/87602/m4/529473
	*Paranasuchus gasparinae* ^a^	MLP‐73‐IV‐15‐1	Late Miocene, Argentina	Partial skull table	Bona et al. (2013)
	*Stangerochampsa mccabei* ^a^	TMP 1986.61.1	Late Cretaceous, Canada	Cranium	Donzé et al. (2026)
Crocodyloidea	*Crocodylus acutus*	MZS Cro 055	Extant, Americas	Skull table	Perrichon et al. (2023)
	*Crocodylus halli*	FLMNH UF 145927	Extant, New Guinea	Cranium	MorphoSource ark:/87602/m4/M95917
	*Crocodylus johnstoni*	OUVC 10425	Extant, Australia	Cranium	Witmer et al. (2008)
	*Crocodylus moreletii*	TMM 4980	Extant, Central America	Cranium	MorphoSource ark:/87602/m4/611715
	*Crocodylus niloticus*	MHNL 50001387 & MZB 2003–1423	Extant, Sub‐Saharan Africa	Skull table & Cranium	Perrichon et al. (2023); MorphoSource ark:/87602/m4/600965
	*Crocodylus novaeguineae*	DVZ M 9/13	Extant, New Guinea	Cranium	Kuzmin et al. (2021)
	*Crocodylus palustris*	MHNL 50001398	Extant, South Asia	Skull table	Perrichon et al. (2023)
	*Crocodylus rhombifer*	NMB.AB50.0171	Extant, Caribbean	Cranium (Holocene)	MorphoSource ark:/87602/m4/615487
	*Crocodylus siamensis*	MHNL 50001389	Extant, Southeast Asia	Skull table	Perrichon et al. (2023)
	*Mecistops cataphractus*	TMM M 3529	Extant, West Africa	Cranium	MorphoSource ark:/87602/m4/M114916
	*Osteolaemus tetraspis*	FMNH 98936	Extant, West Africa	Cranium	MorphoSource ark:/87602/m4/M114918
	*Paludirex vincenti* ^a^	QMF59017	Cenozoic, Australia	Partial cranium	Ristevski et al. (2020)
	*Trilophosuchus rackhami* ^a^	QMF16856	Middle Miocene, Australia	Skull table	Ristevski (2022)
	*Voay robustus* ^a^	NHMUK PV R 36685	Holocene, Madagascar	Skull table	Perrichon et al. (2024)
Gavialoidea	*Argochampsa krebsi* ^a^	NHMUK PV R36872	Paleocene–early Eocene, Morocco	Cranium	Pligersdorffer et al. (2025)
	*Eogavialis gavialoides* ^a^	NHMUK R3325	Late Eocene–early Oligocene, Egypt	Skull table	Nikon Metrology XTH 225 ST, NHMUK
	*Eosuchus lerichei* ^a^	IRSNB R49	Late Paleocene, Belgium	Cranium	Burke et al. (2025)
	*Eothoracosaurus mississippiensis* ^a^	DSM 3293	Maastrichtian (Late Cretaceous), USA	Cranium	Boerman et al. (in review)
	*Gavialis gangeticus*	FLMNH UF 118998	Extant, South Asia	Cranium	Burke and Mannion (2023)
	*Gavialosuchus eggenburgensis* ^a^	KME	Miocene, Austria	Cranium	Gmünd CT Diagnostic Center, Austria
	*Gryposuchus neogaeus* ^a^	MLP 68‐IX‐V‐1	Late Miocene, Argentina	Partial skull table	Bona et al. (2015)
	*Gunggamarandu maunala* ^a^	QMF 14.548	Pliocene–Pleistocene, Australia	Partial skull table	Ristevski et al. (2021)
	*Kentisuchus spenceri* ^a^	NHMUK R19633	Early Eocene, United Kingdom	Partial skull	Nikon Metrology XTH 225 ST, NHMUK
	*Piscogavialis jugaliperforatus* ^a^	SMNK 1282	Late Miocene, Peru	Cranium	KIT
	*Portugalosuchus azenhae* ^a^	ML1818	Cenomanian (Late Cretaceous), Portugal	Partial skull table	Puértolas‐Pascual et al. (2023)
	*Sutekhsuchus dowsoni* ^a^	NHMUK PV R4769	Early Miocene, Egypt	Cranium	Burke and Mannion (2023)
	*Thecachampsa americana* ^a^	SMM P86.8.1	Miocene, USA	Cranium	SMM
	*Thoracosaurus isorhynchus* ^a^	MGL 54101 & LO 3076 T	Maastrichtian–early Paleocene, Europe	Partial skulls	Boerman et al. (in review)
	‘*Tomistoma*’ *cairense* ^a^	SMNS 10575	Middle Eocene, Egypt	Partial skull	Burke et al., (in review)
	*Tomistoma schlegelii*	TMM M6342 & NHMUK 1893.3.6.14	Extant, Indonesia and Malaysia	Cranium & skull table	Burke and Mannion (2023); Perrichon et al. (2023)

^a^ Extinct taxa.

CT scan data were reconstructed using Avizo v 9.7 (FEI Visualisation Science Group; https://www.thermofisher.com), in which the internal cranial anatomy was segmented manually for most of the species evaluated, with the exception of *Agaresuchus fontisensis*, *Arenysuchus gascabadiolorum*, *Crocodylus johnstoni*, *Diplocynodon ratelii*, *Diplocynodon tormis*, *Gryposuchus neogaeus*, *Gunggamarandu maunala*, *Leidyosuchus canadensis*, *Mourasuchus arendsi*, *Paludirex vincenti*, *Paralligator gradilifrons*, *Paranasuchus gasparinae*, *Stangerochampsa mccabei*, *Trilophosuchus rackhami* and *Voay robustus*, for which reconstructions were taken from their respective literature (see Table 1). The subsequent 3D models were smoothed in Blender (Stichting Blender Foundation, Amsterdam) and formatted in Inkscape (Inkscape Project, 2020).

To facilitate discussion and map out endocranial anatomical changes in a phylogenetic context, we assembled an informal supertree (Figure 1) of the 43 taxa included in our study, based on combining topologies from recent literature. The relationships of extant species follow recent molecular‐based topologies (e.g. Hekkala et al., 2021; Pan et al., 2021). The phylogenetic position of several extinct taxa included in this study differs notably between studies. *Diplocynodon* and *Leidyosuchus* are typically recovered as early‐diverging alligatoroids (e.g. Rio & Mannion, 2021), but have recently been placed outside of Crocodylia (Walter et al., 2025). The ‘thoracosaurs’ have been recovered within Gavialoidea in some studies (e.g. Burke et al., 2025), but outside of Crocodylia in others (e.g. Zamora‐Vega et al., 2025). *Trilophosuchus rackhami* (and Mekosuchinae more broadly) has often been recovered as member of Crocodyloidea, but has also been placed outside of Longirostres (e.g. Ristevski et al., 2023). Lastly, *Voay robustus* has usually been recovered as an osteolaemine (e.g. Brochu, 2007), but was recently positioned as a crocodyline based on palaeogenomic data (Hekkala et al., 2021). Here, we include *Diplocynodon* and *Leidyosuchus* as alligatoroids, ‘thoracosaurs’ as gavialoids, *Trilophosuchus* as a crocodyloid and *Voay* as a crocodyline (Figure 1).

**FIGURE 1:**
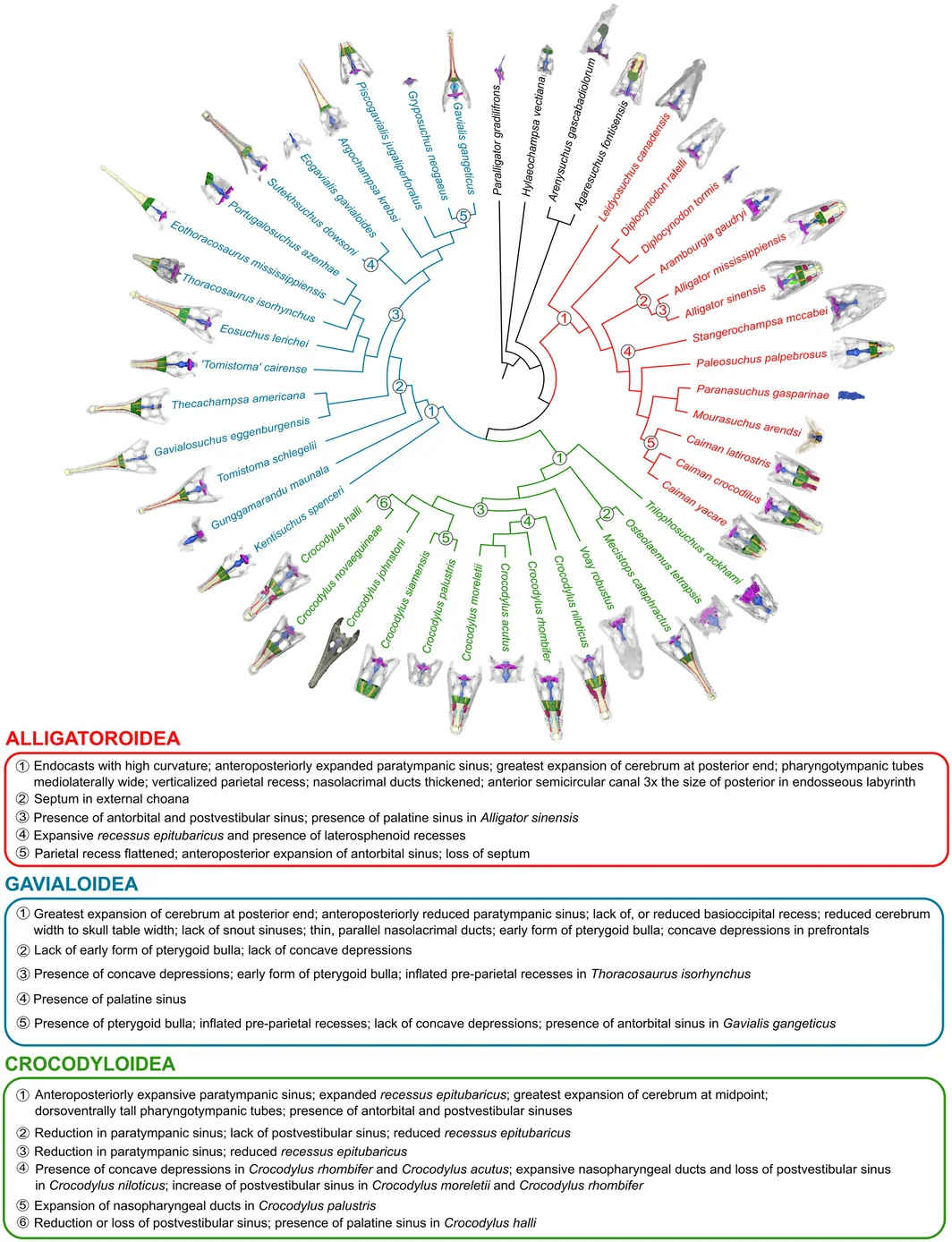
Supertree showing the interrelationships of Crocodylia based on topologies from Hekkala et al. (2021); Pan et al. (2021); Rio & Mannion (2021) and Burke et al. (2024).

## 3 RESULTS

### 3.1 Encephalic endocast

As the brain of extant crocodylians does not fill the entirety of the endocranial cavity, the encephalic endocast is considered a cast of the dural envelope (Brusatte et al., 2016; Hopson, 1979; Jerison, 1973; Rogers, 1999; Witmer et al., 2008). The encephalic endocast itself is divided into three sections: the forebrain (prosencephalon), the midbrain (mesencephalon) and the hindbrain (rhombencephalon). The forebrain comprises the olfactory bulb, olfactory tract and the cerebrum. The olfactory bulb, located at the most anterior point of the encephalic endocast, connects to the olfactory region of the nasal cavity via the olfactory nerve (Cranial Nerve I). The olfactory bulb contains mitral cells and odour receptors, and the relative size of the bulb has been hypothesised to indicate olfactory capabilities (Zelenitsky et al., 2011). Olfactory capabilities have been estimated in fossil species across Crocodylomorpha, which show relatively similar capabilities to extant crocodylian species (Serrano‐Martínez et al., 2021).

The cerebrum represents the greatest expansion in the encephalic endocast, which has been associated with refined sensory inputs in birds and mammals, as a larger cerebral region implies a greater neuronal area to execute complex behaviours (Pierce et al., 2017; Rogers, 1999). At the most ventral point of the cerebrum is the pituitary fossa, which houses the pituitary gland, responsible for the secretion of hormones. In reptiles, this is thought to have an antidiuretic effect, wherein the glomerular capillaries are constricted, leading to the sub‐sequential decrease of blood flow and water loss (Heller, 1942, 1950).

The mesencephalon region of the encephalic endocast has been used to estimate the location of the optic lobes in fossil species (Serrano‐Martínez et al., 2021). Optic lobes are most prominent early in the ontogeny of extant crocodylians; throughout development, the optic lobes and the dural envelope that surrounds them subside on the endocast, to the point where skeletally mature individuals lack distinct optic lobes (Hu et al., 2021; Jirak & Janacek, 2017; Ristevski, 2022). As a result, they can only be inferred from the endocast in a narrowing between the cerebral hemispheres and the pituitary fossa, and from the otic capsule concavities of the rhombencephalon (Jirak & Janacek, 2017; Serrano‐Martínez et al., 2021; Watanabe et al., 2019).

Cephalic and pontine flexure angles pertain to the degree of flexion of the encephalic endocast (see Hopson, 1979; Pierce et al., 2017). The encephalic endocast of non‐crocodylian eusuchians is relatively straight in lateral view; amongst them, *Agaresuchus fontisensis* shows the greatest curvature, with *Arenysuchus gascabadiolorum* displaying the least degree of curvature (see Serrano‐Martínez et al., 2021; Puértolas‐Pascual et al., 2022; Table 2). In all four non‐crocodylian eusuchians evaluated herein, the olfactory bulb is difficult to distinguish from the rest of the olfactory tract in dorsal view, with only a gradual increase in mediolateral width anteriorly (e.g. *Hylaeochampsa*; Figure 2). The olfactory bulb is more clearly distinguishable in lateral view in *Hylaeochampsa*, characterized by an increase in dorsoventral height anteriorly (Figure 2f). In *Paralligator*, the olfactory bulb is bifurcated at its most anterior point (Kuzmin et al., 2025: fig. 16). The cerebrum is difficult to distinguish in both dorsal and lateral views in *Agaresuchus* (Serrano‐Martínez et al., 2021); however, in *Hylaeochampsa* and *Paralligator*, the greatest expansion in transverse width is at the most posterior point, narrowing in width anteriorly (Figure 2; Kuzmin et al., 2025).

**TABLE 2:** List of measurements of the endocasts and endosseous labyrinths for all taxa included in the study.

Clade	Species	CW: SW	CF	PF	AA:PA	NC:SnW
Non‐crocodylian eusuchian	*Agaresuchus fontisensis* ^a^	0.19	135°	153°	?	0.28
	*Arenysuchus gascabadiolorum* ^a^	0.40	155°	156°	?	?
	*Hylaeochampsa vectiana* ^a^	0.32	142°	157°	?	?
	*Paralligator gradilifrons* ^a^	0.44	139°	144°	?	?
Alligatoroidea	*Alligator mississippiensis*	0.32	120°	139°	6.97	0.39
	*Alligator sinensis*	0.33	123°	139°	2.87	0.39
	*Arambourgia gaudryi* ^a^	0.40	126°	136°	3.67	?
	*Caiman crocodilus*	0.36	129°	152°	3.85	0.39
	*Caiman latirostris*	0.38	128°	140°	3.45	0.29
	*Caiman yacare*	0.34	123°	144°	4.44	0.31
	*Diplocynodon ratelii* ^a^	0.33	?	?	?	?
	*Diplocynodon tormis* ^a^	?	137°	145°	?	?
	*Leidyosuchus canadensis* ^a^	0.27	139°	140°		
	*Mourasuchus arendsi* ^a^	?	?	?	?	?
	*Paleosuchus palpebrosus*	0.44	103°	130°	?	0.33
	*Paranasuchus gasparinae* ^a^	?	?	?	?	?
	*Stangerochampsa mccabei* ^a^	0.34	137°	145°	?	?
Crocodyloidea	*Crocodylus acutus*	0.32	?	137°	3.19	?
	*Crocodylus halli*	0.35	133°	140°	2.23	0.49
	*Crocodylus johnstoni*	0.44	130°	144°	?	?
	*Crocodylus moreletii*	0.37	138°	136°	1.88	0.26
	*Crocodylus niloticus*	0.26	146°	158°	2.97	0.33
	*Crocodylus novaeguineae*	0.49	143°	155°	2.22	0.43
	*Crocodylus palustris*	0.26	133°	150°	2.39	?
	*Crocodylus rhombifer*	0.42	143°	154°	2.55	0.27
	*Crocodylus siamensis*	0.31	128°	134°	2.35	?
	*Mecistops cataphractus*	0.39	125°	139°	5.81	0.30
	*Osteolaemus tetraspis*	0.37	?	153°	6.17	?
	*Paludirex vincenti* ^a^	?	?	?	?	?
	*Trilophosuchus rackhami* ^a^	0.46	136°	142°	2.00	?
	*Voay robustus* ^a^	0.29	134°	141°	3.19	?
Gavialoidea	*Argochampsa krebsi* ^a^	0.36	123°	128°	?	0.46
	*Eogavialis gavialoides* ^a^	0.19	139°	150°	?	?
	*Eosuchus lerichei* ^a^	0.27	158°	155°	3.17	0.39
	*Eothoracosaurus mississippiensis* ^a^	0.22	168°	166°	?	0.54
	*Gavialis gangeticus*	0.19	150°	154°	3.33	0.38
	*Gavialosuchus eggenburgensis* ^a^	0.18	130°	134°	?	0.38
	*Gryposuchus neogaeus* ^a^	?	?	?	?	?
	*Gunggamarandu maunala* ^a^	?	?	?	?	?
	*Kentisuchus spenceri* ^a^	0.29	140°	149°	?	0.42
	*Piscogavialis jugaliperforatus* ^a^	0.20	135°	149°	2.14	0.55
	*Portugalosuchus azenhae* ^a^	?	152°	156°	3.01	?
	*Sutekhsuchus dowsoni* ^a^	0.28	144°	149°	2.10	0.43
	*Thecachampsa americana* ^a^	0.26	?	?	?	0.49
	*Thoracosaurus isorhynchus* ^a^	0.16	150°	152°	1.95	0.48
	‘*Tomistoma*’ *cairense* ^a^	?	132°	153°	?	0.46
	*Tomistoma schlegelii*	0.36	130°	146°	2.42	?

Abbreviations: AA, anterior semicircular canal area; CF, cephalic flexure angle; CW, cerebrum width; NC, nasal cavity width; PF, pontine flexure angle; PP, posterior semicircular canal area; SnW, snout width; SW, skull width.

^a^ Extinct taxa.

**FIGURE 2:**
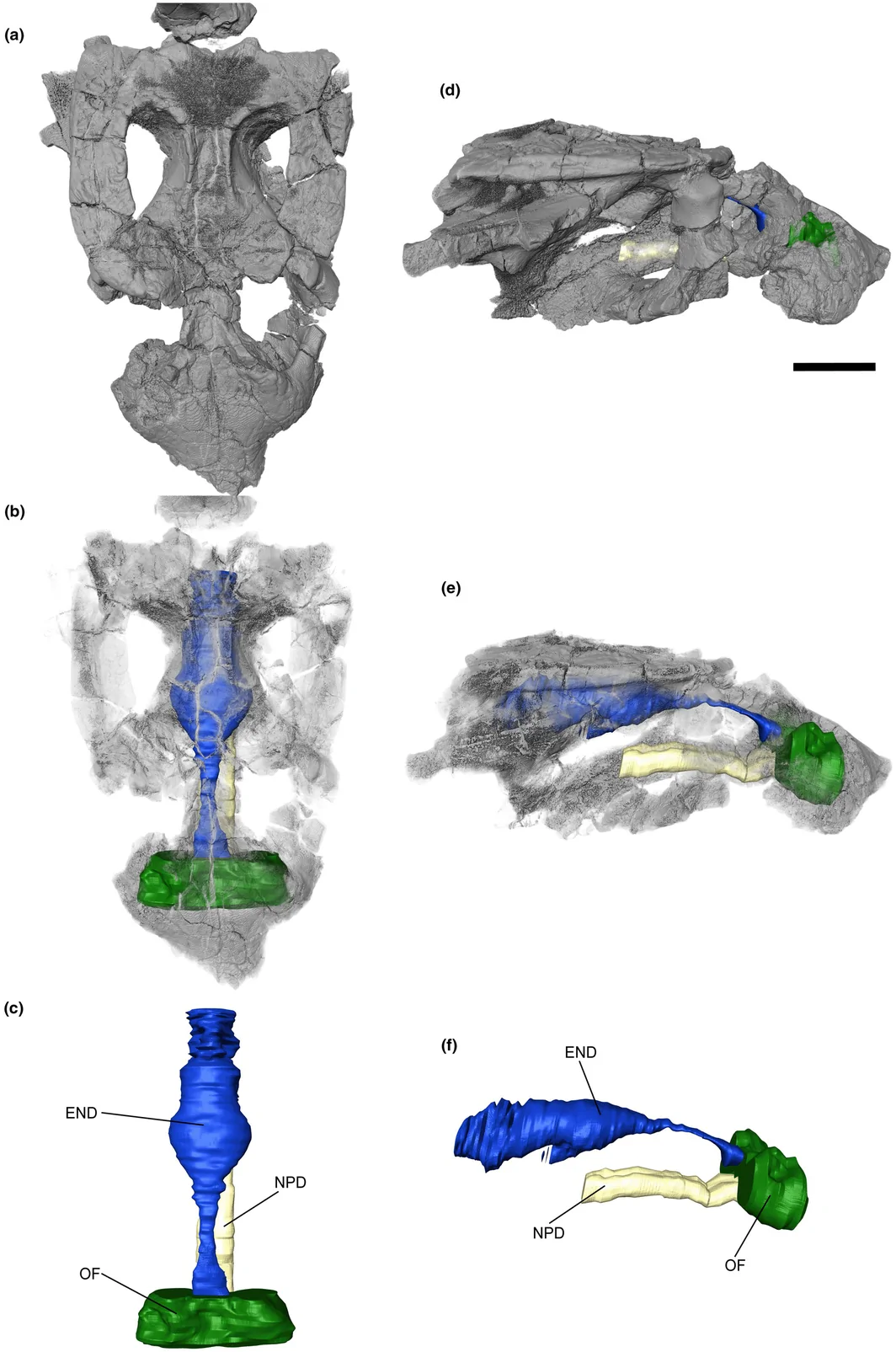
Skull of *Hylaeochampsa vectiana* (NHMUK R177) (a) skull rendering in dorsal view; (b) skull rendered transparent with internal anatomy visible in dorsal view; (c) internal anatomy of *Hylaeochampsa vectiana* in dorsal view; (d) skull rendering in lateral view; (b) skull rendered transparent with internal anatomy visible in lateral view; (c) internal anatomy of *Hylaeochampsa vectiana* in lateral view. END, endocast; OF, olfactory region; NPD, nasopharyngeal duct. Scale bar = 2 cm.

Within Crocodylia, the encephalic endocasts of Alligatoroidea (Figures 3, 4, 5, 6, 7, 8, 9, 10, 11, 12) show the greatest degree of curvature in lateral view, reflected by low cephalic and pontine flexure angles (Table 2). *Paleosuchus palpebrosus* shows the greatest curvature within Alligatoroidea, with *Diplocynodon tormis* displaying the least curvature in the clade (Serrano‐Martínez et al., 2019; Table 2). Most alligatoroids are characterized by an olfactory bulb in which the anteroposterior length is greater than half that of the olfactory tract, with *Caiman crocodilus* (Figure 6c), *Leidyosuchus canadensis* (Figure 10c) and *Paleosuchus palpebrosus* (Figure 11c) being exceptions within the clade. The greatest transverse width of the cerebrum in Alligatoroidea is at its posterior end, before narrowing in width anteriorly towards the olfactory tract, comparable to the condition in *Hylaeochampsa* and *Paralligator*. Except for *Leidyosuchus*, the transverse width of the cerebrum of alligatoroids is more than one third that of the skull table (Table 2).

**FIGURE 3:**
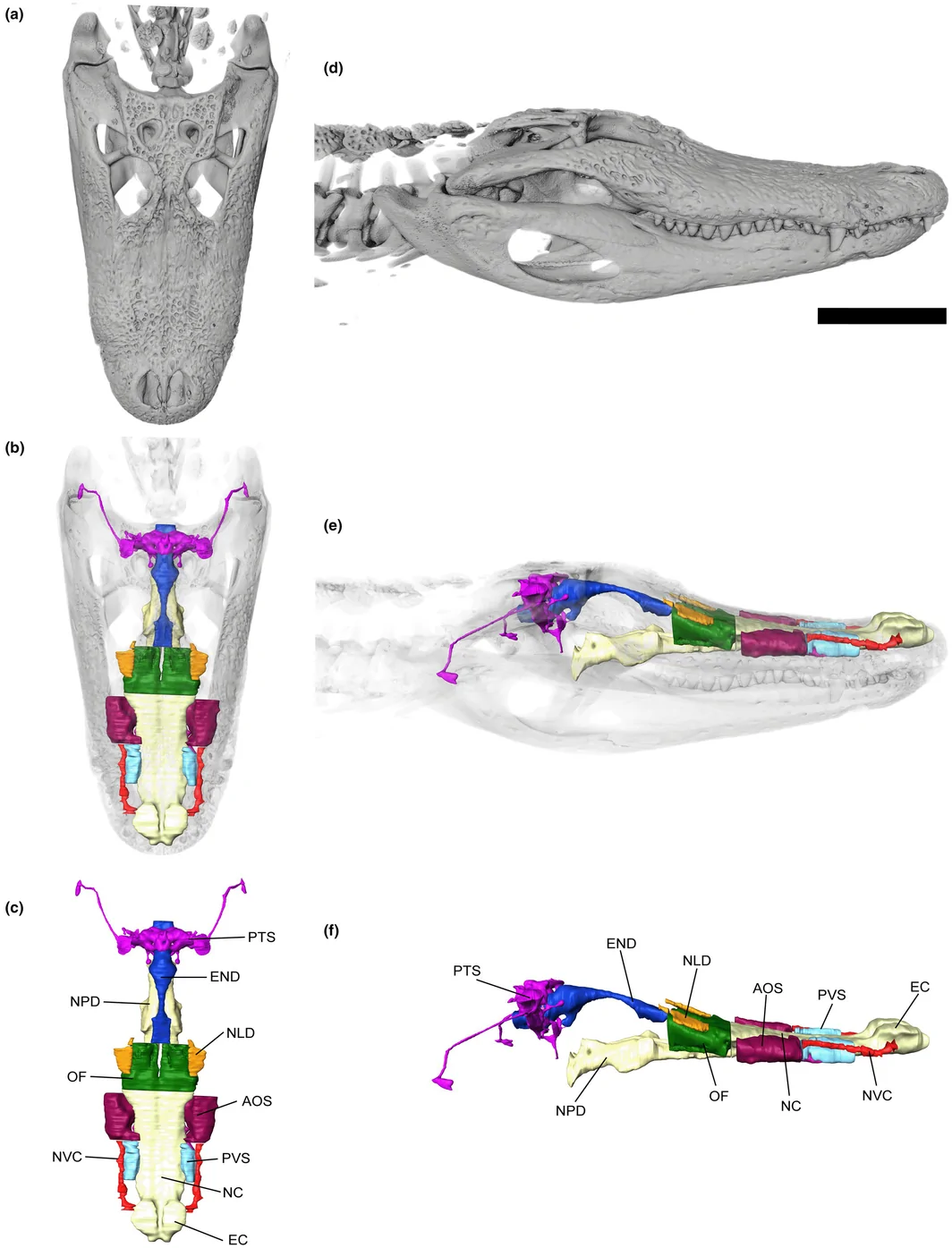
Skull of *Alligator mississippiensis* (OUVC 9761). (a) skull rendering in dorsal view; (b) skull rendered transparent with internal anatomy visible in dorsal view; (c) internal anatomy of *Alligator mississippiensis* in dorsal view; (d) skull rendering in lateral view; (e) skull rendered transparent with internal anatomy visible in lateral view; (f) internal anatomy of *Alligator mississippiensis* in lateral view. AOS, antorbital sinus; EC, external choana; END, endocast; NC, nasal cavity; NLD, nasolacrimal duct; NPD, nasopharyngeal duct; NVC, neurovascular canals; OF, olfactory region; PTS, paratympanic sinus; PVS, postvestibular sinus. Scale bar = 10 cm.

**FIGURE 4:**
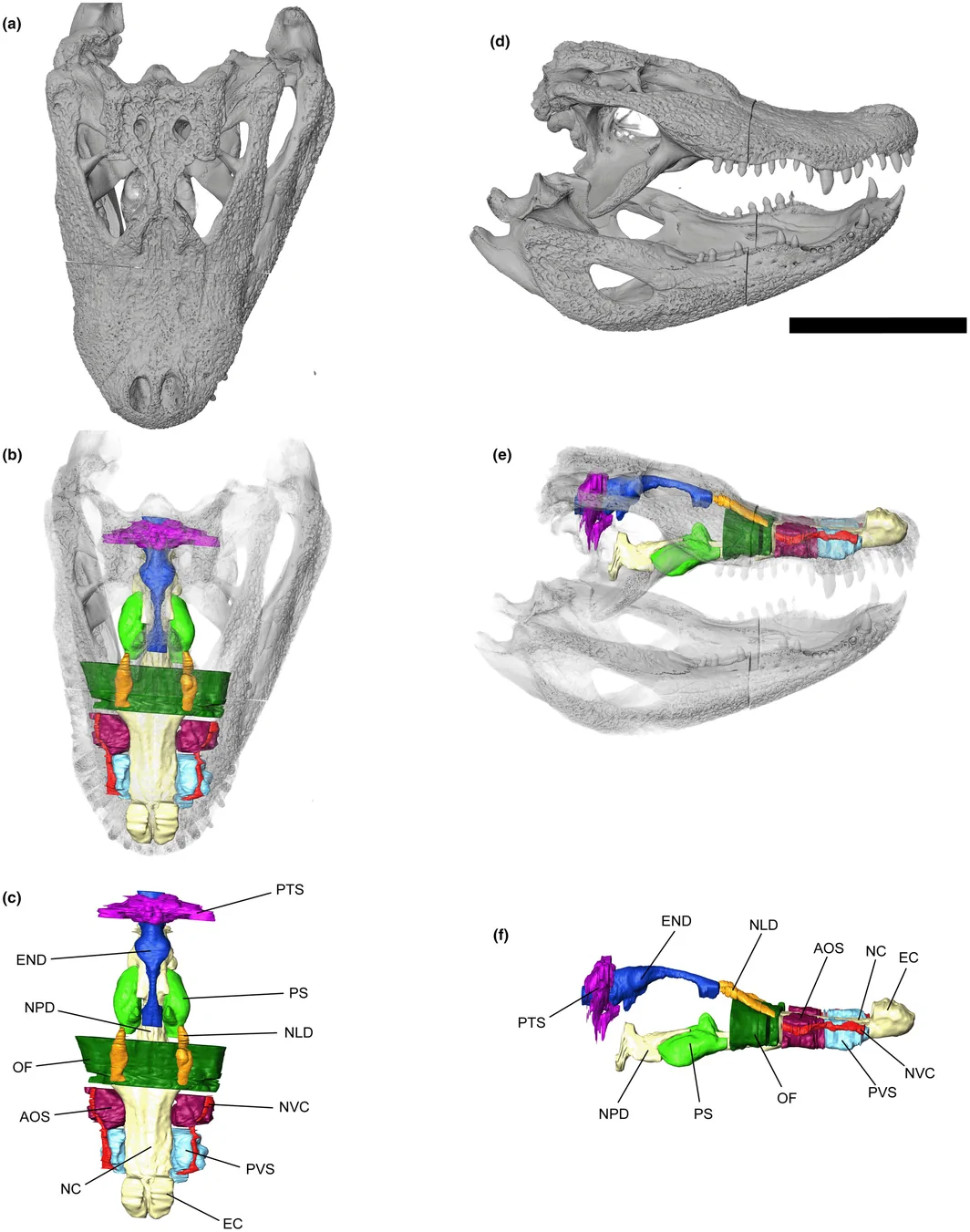
Skull of *Alligator sinensis* (NHMW‐Zoo‐HS‐37966) adapted from Donzé et al. (2026). (a) skull rendering in dorsal view; (b) skull rendered transparent with internal anatomy visible in dorsal view; (c) internal anatomy of *Alligator sinensis* in dorsal view; (d) skull rendering in lateral view; (e) skull rendered transparent with internal anatomy visible in lateral view; (f) internal anatomy of *Alligator sinensis* in lateral view. AOS, antorbital sinus; EC, external choana; END, endocast; NC, nasal cavity; NLD, nasolacrimal duct; NPD, nasopharyngeal duct; NVC, neurovascular canals; OF, olfactory region; PS, palatine sinus; PTS, paratympanic sinus; PVS, postvestibular sinus. Scale bar = 10 cm.

**FIGURE 5:**
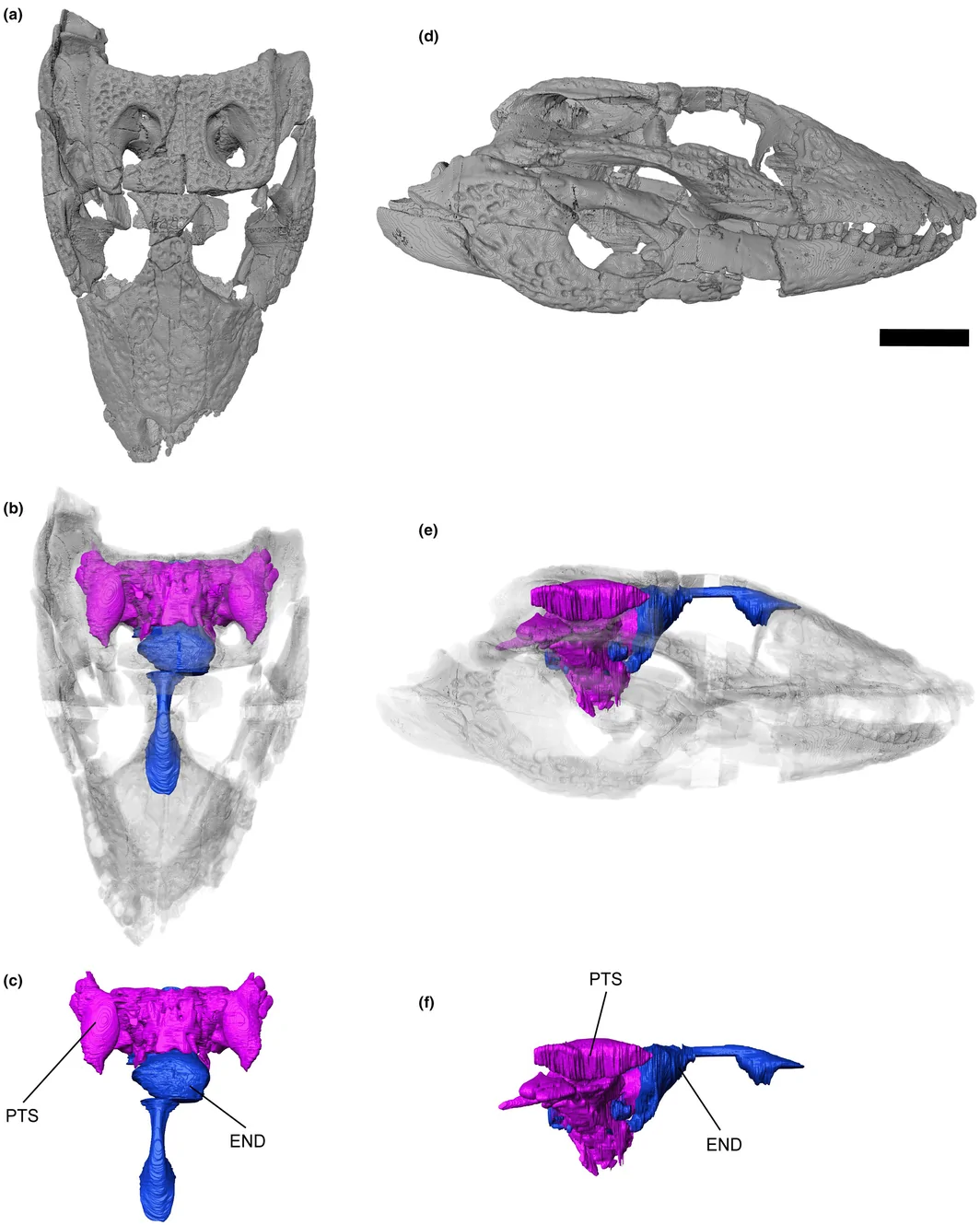
Skull of *Arambourgia gaudryi* (MNHN QU17155) adapted from Conedera et al. (2023). (a) skull rendering in dorsal view; (b) skull rendered transparent with internal anatomy visible in dorsal view; (c) internal anatomy of *Arambourgia gaudryi* in dorsal view; (d) skull rendering in lateral view; (e) skull rendered transparent with internal anatomy visible in lateral view; (f) internal anatomy of *Arambourgia gaudryi* in lateral view. END, endocast; PTS, paratympanic sinus. Scale bar = 2 cm.

**FIGURE 6:**
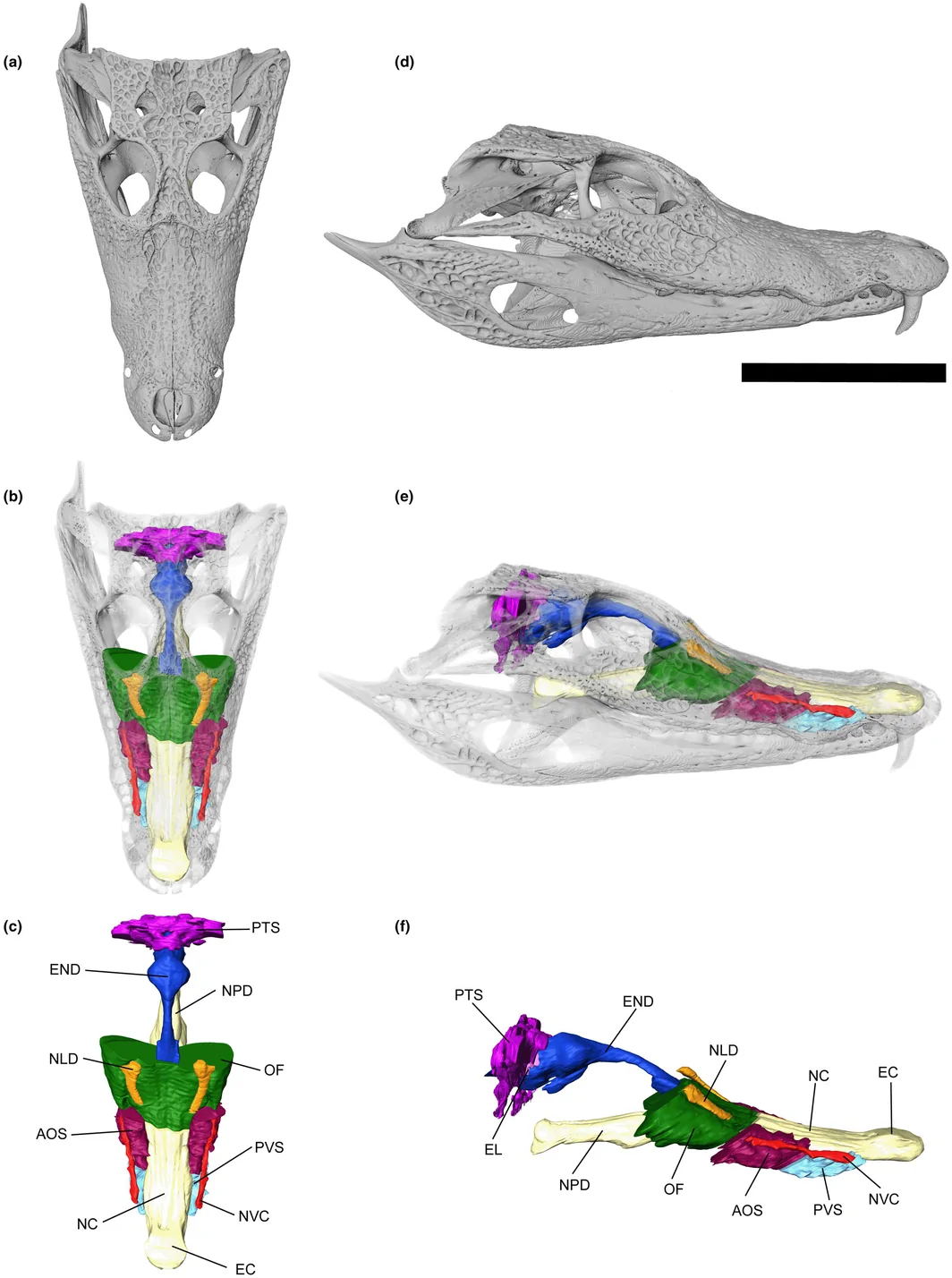
Skull of *Caiman crocodilus* (UMMZ 128024). (a) skull rendering in dorsal view; (b) skull rendered transparent with internal anatomy visible in dorsal view; (c) internal anatomy of *Caiman crocodilus* in dorsal view; (d) skull rendering in lateral view; (e) skull rendered transparent with internal anatomy visible in lateral view; (f) internal anatomy of *Caiman crocodilus* in lateral view. AOS, antorbital sinus; EC, external choana; EL; endosseous labyrinth; END, endocast; NC, nasal cavity; NLD, nasolacrimal duct; NPD, nasopharyngeal duct; NVC, neurovascular canals; OF, olfactory region; PVS, postvestibular sinus. Scale bar = 10 cm.

**FIGURE 7:**
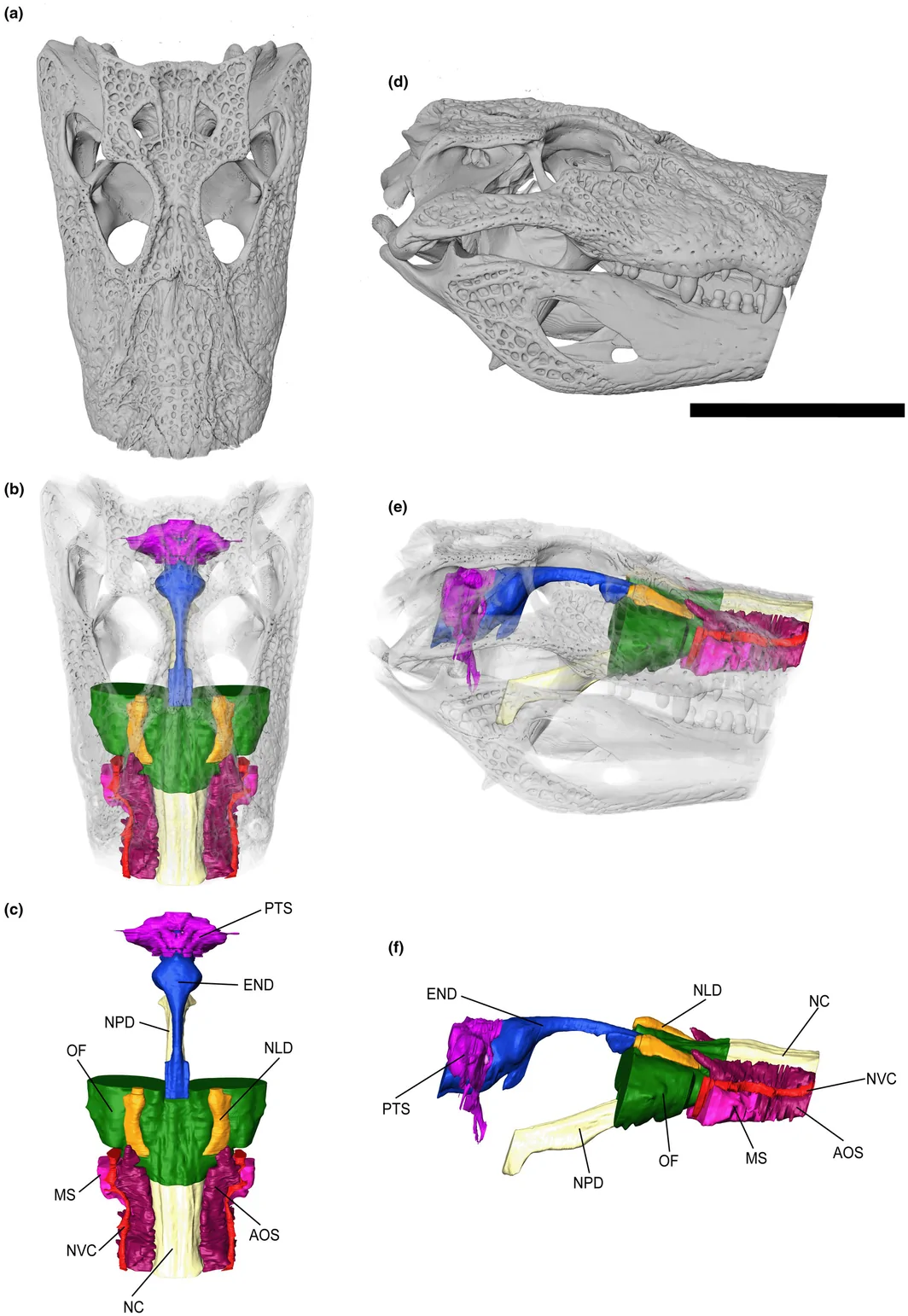
Skull of *Caiman latirostris* (UMMZ 155287). (a) skull rendering in dorsal view; (b) skull rendered transparent with internal anatomy visible in dorsal view; (c) internal anatomy of *Caiman latirostris* in dorsal view; (d) skull rendering in lateral view; (e) skull rendered transparent with internal anatomy visible in lateral view; (f) internal anatomy of *Caiman latirostris* in lateral view. AOS, antorbital sinus; END, endocast; MS, maxillary sinus; NC, nasal cavity; NLD, nasolacrimal duct; NPD, nasopharyngeal duct; NVC, neurovascular canals; OF, olfactory region; PTS, paratympanic sinus. Scale bar = 10 cm.

**FIGURE 8:**
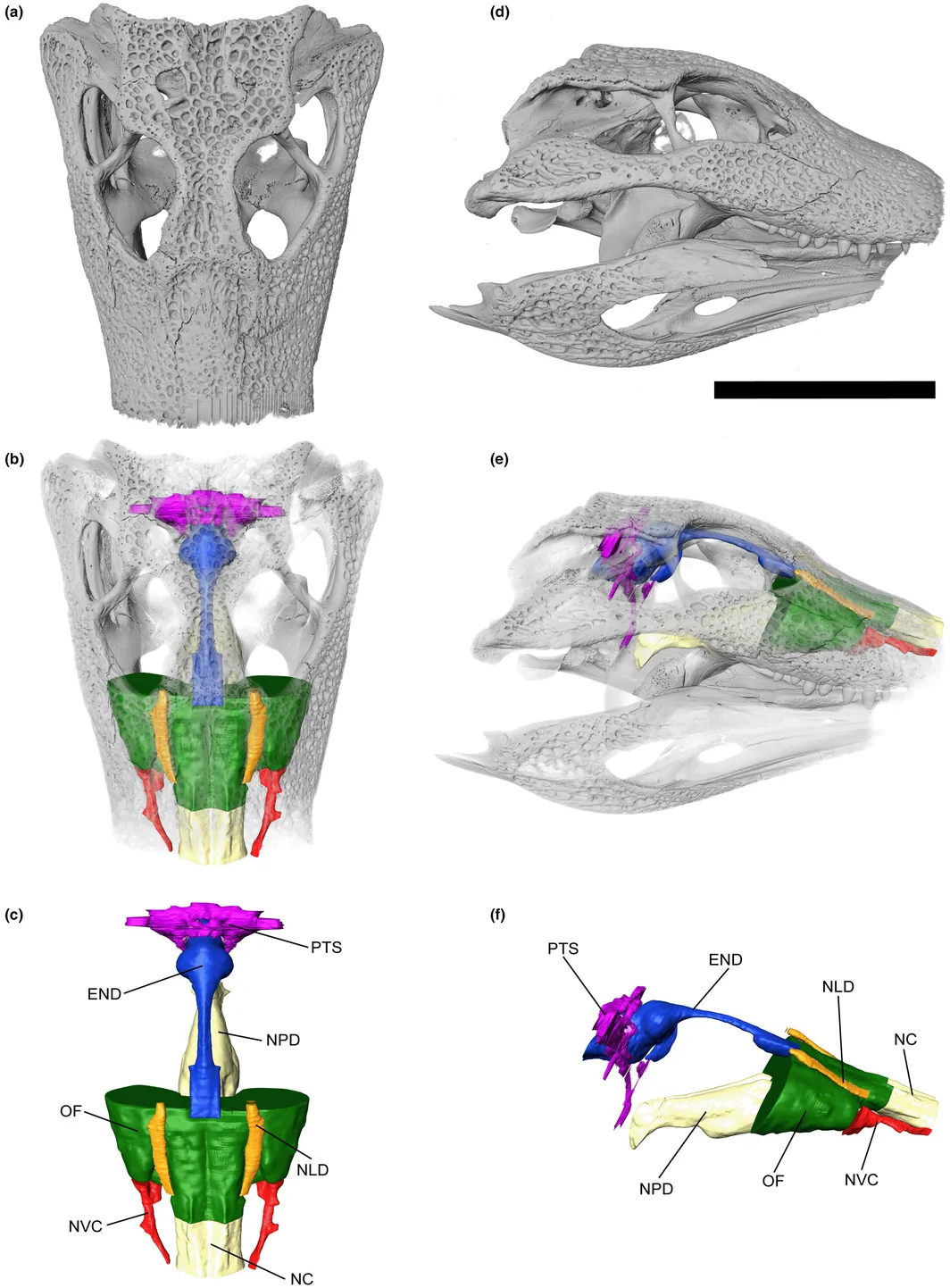
Skull of *Caiman yacare* (UMMZ 155289). (a) skull rendering in dorsal view; (b) skull rendered transparent with internal anatomy visible in dorsal view; (c) internal anatomy of *Caiman yacare* in dorsal view; (d) skull rendering in lateral view; (e) skull rendered transparent in lateral view; (f) internal anatomy of *Caiman yacare* in lateral view. END, endocast; NC, nasal cavity; NLD, nasolacrimal duct; NPD, nasopharyngeal duct; NVC, neurovascular canals; OF, olfactory region; PTS, paratympanic sinus. Scale bar = 10 cm.

**FIGURE 9:**
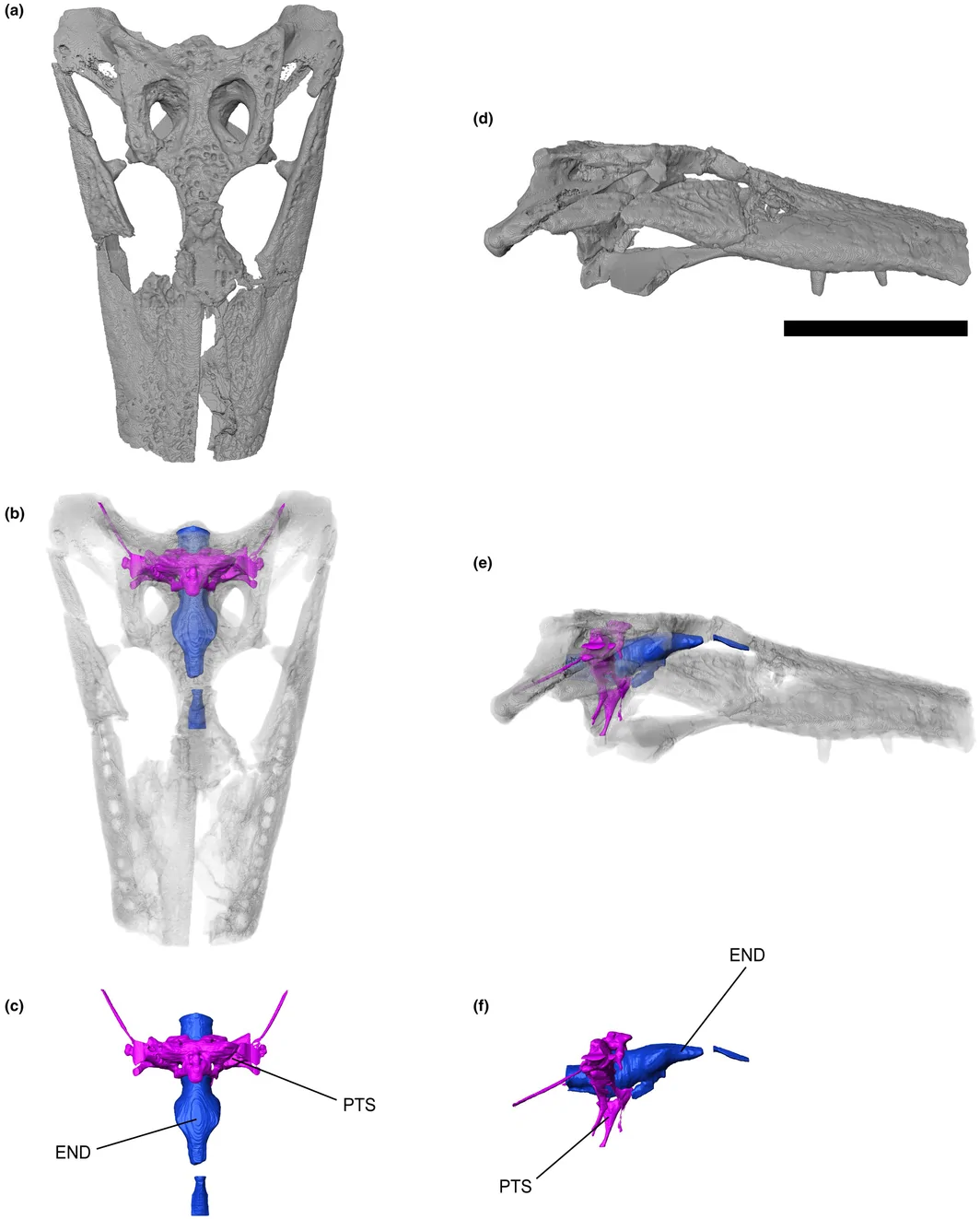
Skull of *Diplocynodon ratelii* (MHNL‐LA86) adapted from Donzé et al. (2026). (a) skull rendering in dorsal view; (b) skull rendered transparent with internal anatomy visible in dorsal view; (c) internal anatomy of *Diplocynodon ratelii* in dorsal view; (d) skull rendering in lateral view; (e) skull rendered transparent in lateral view; (f) internal anatomy of *Diplocynodon ratelii* in lateral view. END, endocast; PTS, paratympanic sinus. Scale bar = 10 cm.

**FIGURE 10:**
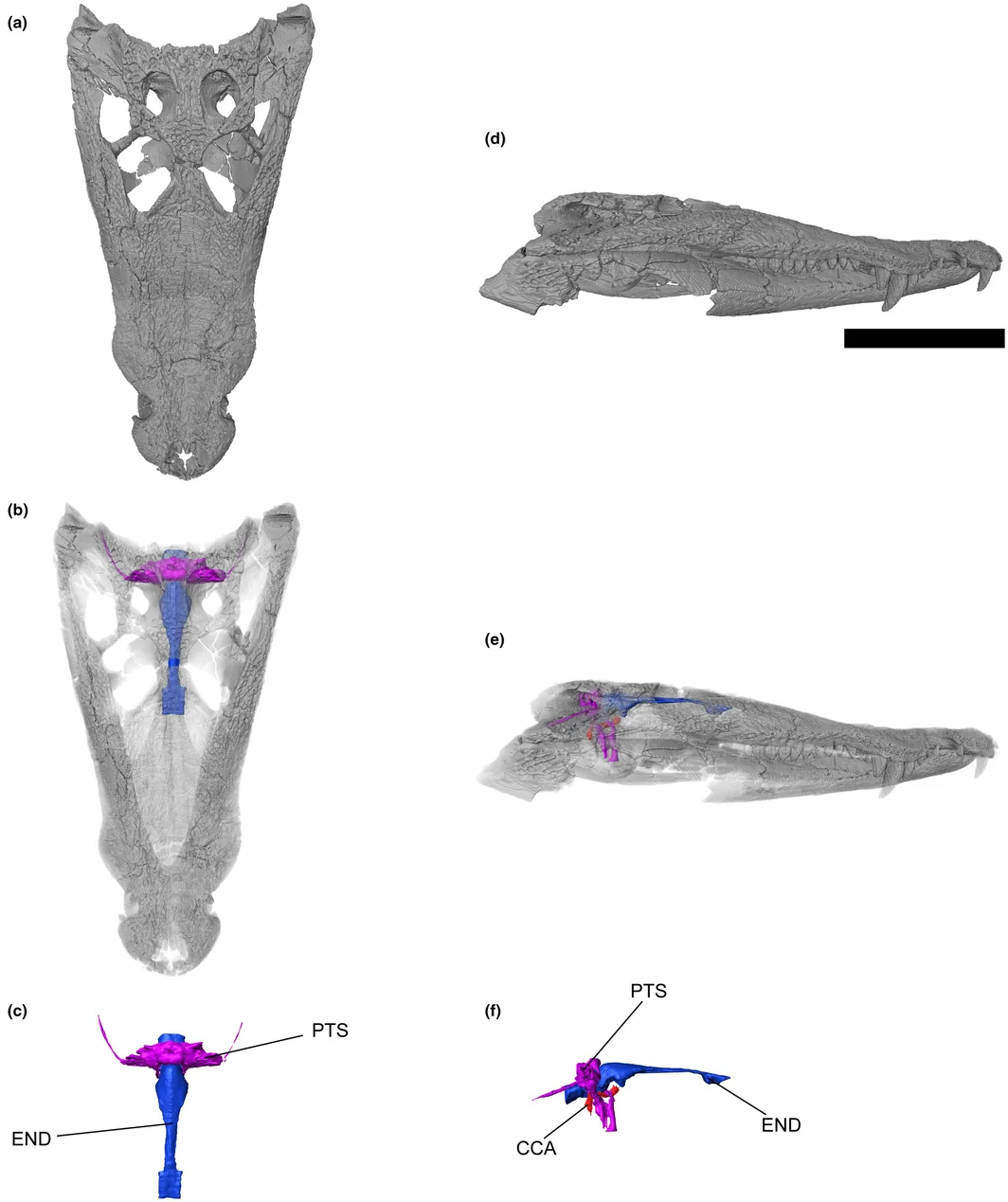
Skull of *Leidyosuchus canadensis* (TMP 1986.221.1) adapted from Donzé et al. (2026). (a) skull rendering in dorsal view; (b) skull rendered transparent with internal anatomy visible in dorsal view; (c) internal anatomy of *Leidyosuchus canadensis* in dorsal view; (d) skull rendering in lateral view; (e) skull rendered transparent in lateral view; (f) internal anatomy of *Leidyosuchus canadensis* in lateral view. CCA, cephalic carotid arteries; END, endocast; PTS, paratympanic sinus. Scale bar = 10 cm.

**FIGURE 11:**
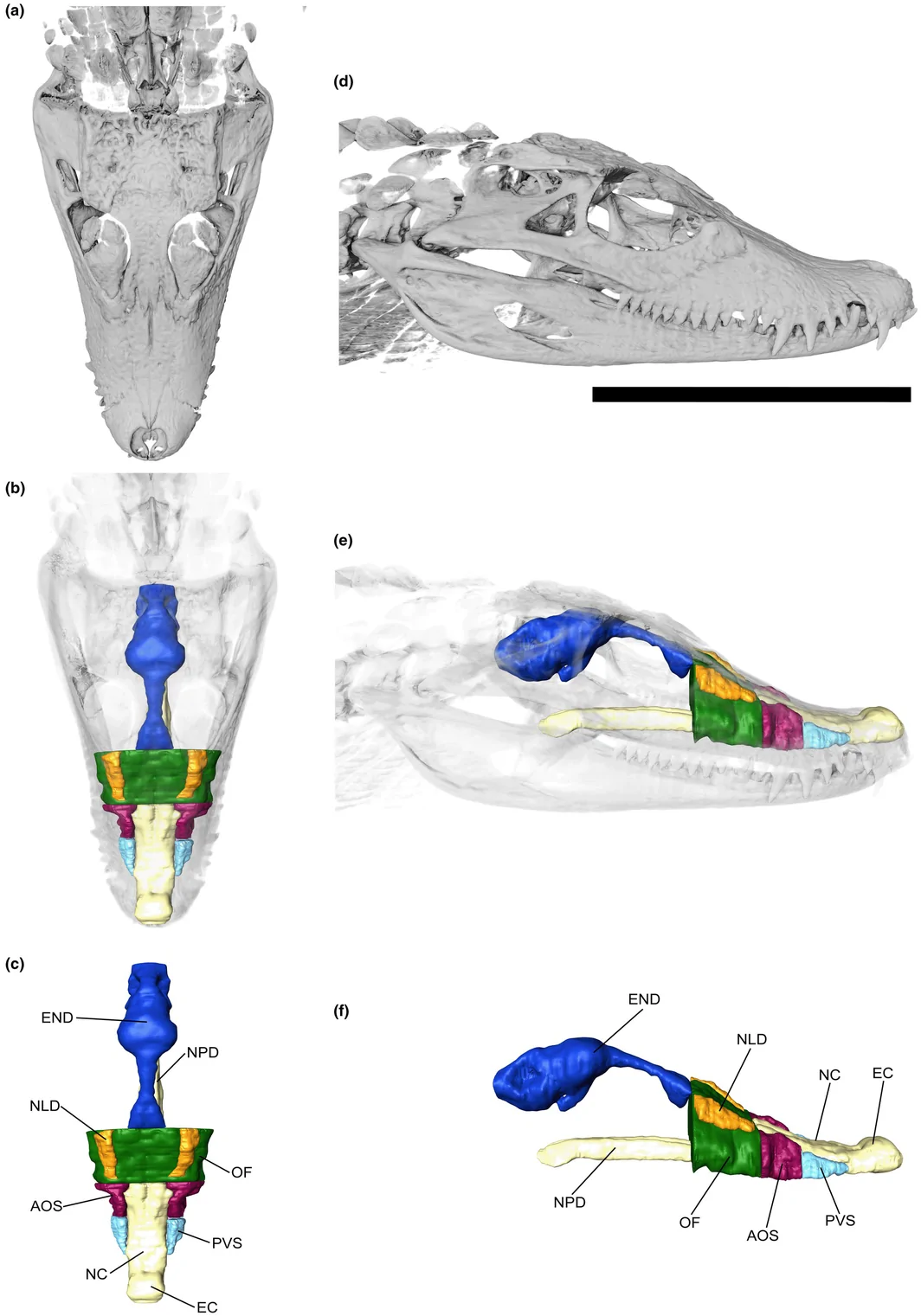
Skull of *Paleosuchus palpebrosus*. (a) skull rendering in dorsal view; (b) skull rendered transparent with internal anatomy visible in dorsal view; (c) internal anatomy of *Paleosuchus palpebrosus* in dorsal view; (d) skull rendering in lateral view; (e) skull rendered transparent with internal anatomy visible in lateral view; (f) internal anatomy of *Paleosuchus palpebrosus* in lateral view. AOS, antorbital sinus; EC, external choana; END, endocast; NC, nasal cavity; NLD, nasolacrimal duct; NPD, nasopharyngeal duct; OF, olfactory region; PVS, postvestibular sinus. Scale bar = 10 cm.

**FIGURE 12:**
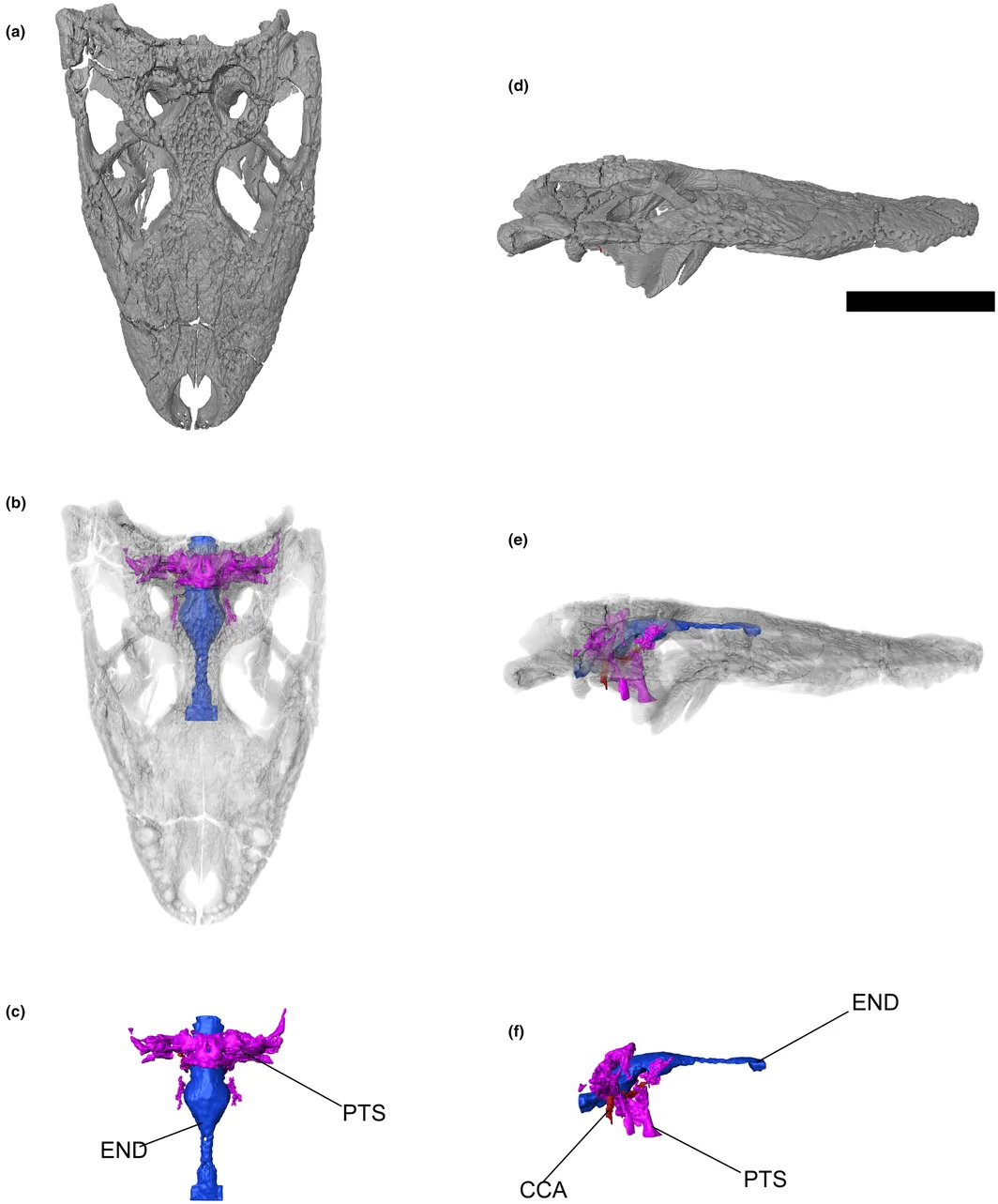
Skull of *Stangerochampsa mccabei* (TMP 1986.61.1) adapted from Donzé et al. (2026). (a) skull rendering in dorsal view; (b) skull rendered transparent with internal anatomy visible in dorsal view; (c) internal anatomy of *Stangerochampsa mccabei* in dorsal view; (d) skull rendering in lateral view; (e) skull rendered transparent in lateral view; (f) internal anatomy of *Stangerochampsa mccabei* in lateral view. CCA, cephalic carotid arteries; END, endocast; PTS, paratympanic sinus. Scale bar = 10 cm.

Contrasting with the encephalic endocasts of Alligatoroidea, crocodyloids (Figures 13, 14, 15, 16, 17, 18, 19, 20, 21, 22, 23, 24) show greater variation in their cephalic and pontine flexure angles; however, overall, they show less curvature in lateral view than alligatoroids. *Crocodylus siamensis* and *Mecistops cataphractus* show the greatest degree of curvature within Crocodyloidea, with *Crocodylus niloticus* displaying the least curvature in this clade (Figures 16, 20 and 21; Table 2). The anteroposterior length of the olfactory bulb in Crocodyloidea is less than one third that of the overall olfactory tract length. The greatest transverse expansion of the cerebrum of most members of Crocodyloidea occurs at its midlength, observed in *Crocodylus acutus* (Figure 13c), *Crocodylus johnstoni* (see Witmer et al., 2008: fig. 6.1), *Crocodylus moreletti* (Figure 15c), *Crocodylus novaeguineae* (Figure 17c), *Crocodylus rhombifer* (Figure 19c), *Crocodylus siamensis* (Figure 20c), *Mecistops cataphractus* (Figure 21c), *Osteolaemus tetraspis* (Figure 22c), *Trilophosuchus rackhami* (Ristevski, 2022; Figure 23c) and *Voay robustus* (Perrichon et al., 2024: Figure 24c). Amongst evaluated crocodyloids, only *Crocodylus halli* (Figure 14c), *Crocodylus niloticus* (Figure 16c,i) and *Crocodylus palustris* (Figure 18c) are characterized by the cerebrum morphology seen in non‐crocodylian eusuchians and alligatoroids. Generally, the transverse width of the cerebrum of crocodyloids is approximately one third that of the skull table, with *Crocodylus niloticus*, *Crocodylus palustris* and *Voay robustus* possessing a cerebrum whose transverse width is less than one third that of the skull table (Table 2).

**FIGURE 13:**
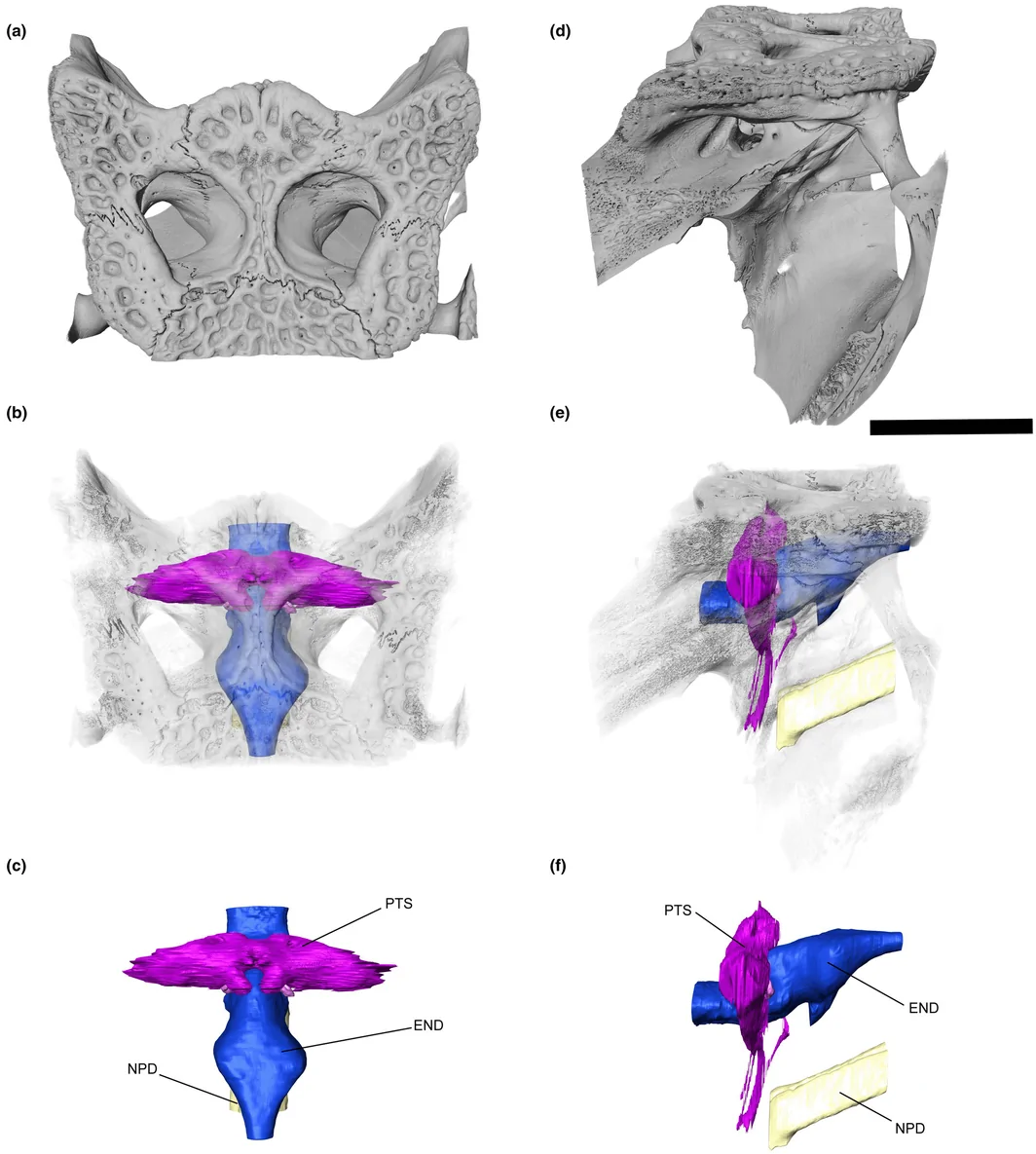
Skull of *Crocodylus acutus* (MZS Cro 055). (a) skull rendering in dorsal view; (b) skull rendered transparent with internal anatomy visible in dorsal view; (c) internal anatomy of *Crocodylus acutus* in dorsal view; (d) skull rendering in lateral view; (e) skull rendered transparent with internal anatomy visible in lateral view; (f) internal anatomy of *Crocodylus acutus* in lateral view. END, endocast; NPD, nasopharyngeal duct; PTS, paratympanic sinus. Scale bar = 5 cm.

**FIGURE 14:**
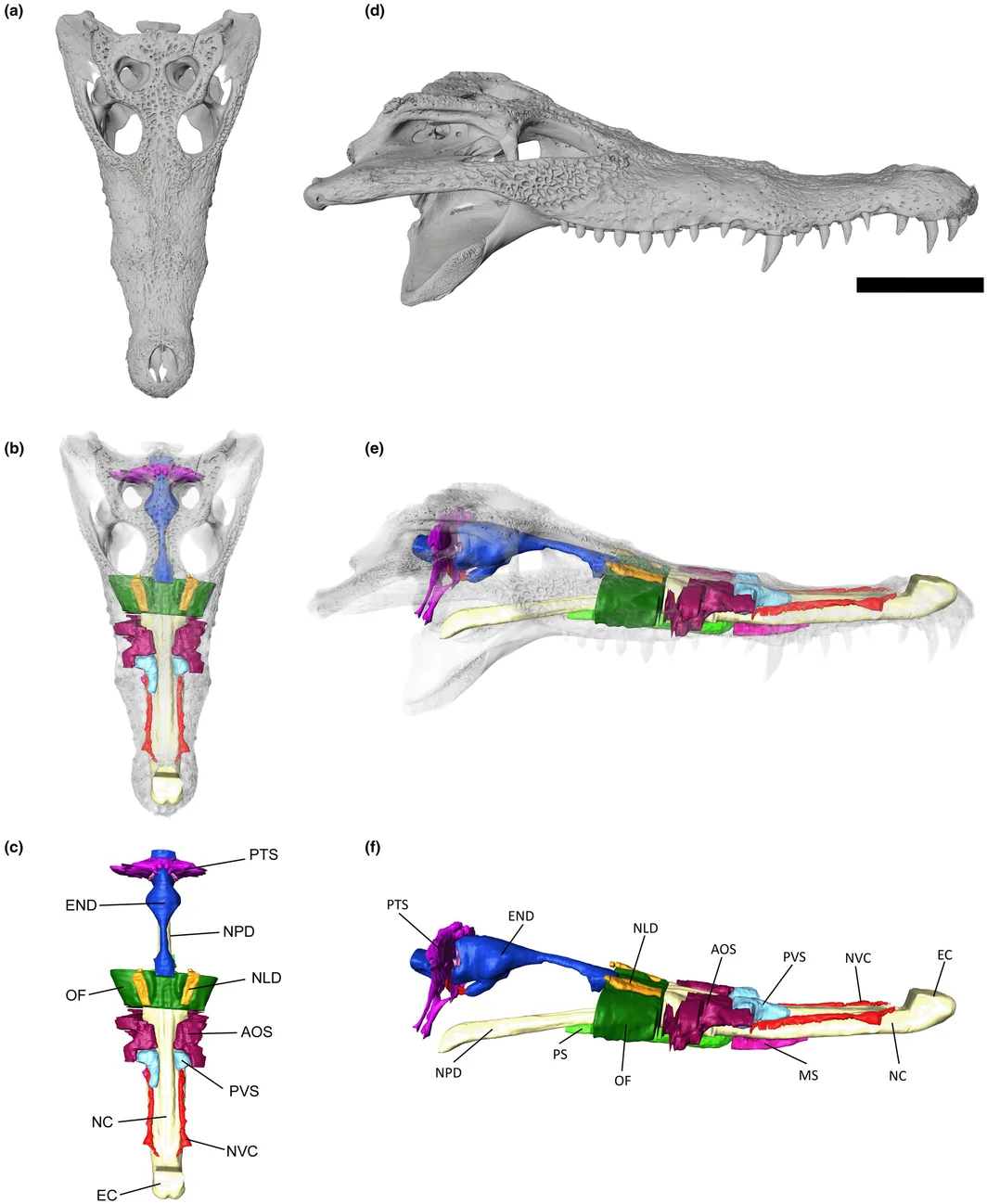
Skull of *Crocodylus halli* (FLMNH UF 145927). (a) skull rendering in dorsal view; (b) skull rendered transparent with internal anatomy visible in dorsal view; (c) internal anatomy of *Crocodylus halli* in dorsal view; (d) skull rendering in lateral view; (e) skull rendered transparent with internal anatomy visible in lateral view; (f) internal anatomy of *Crocodylus halli* in lateral view. AOS, antorbital sinus; EC, external choana; END, endocast; MS, maxillary sinus; NC, nasal cavity; NLD, nasolacrimal duct; NPD, nasopharyngeal duct; NVC, neurovascular canal; OF, olfactory region; PS, palatine sinus; PTS, paratympanic sinus; PVS, postvestibular sinus. Scale bar = 10 cm.

**FIGURE 15:**
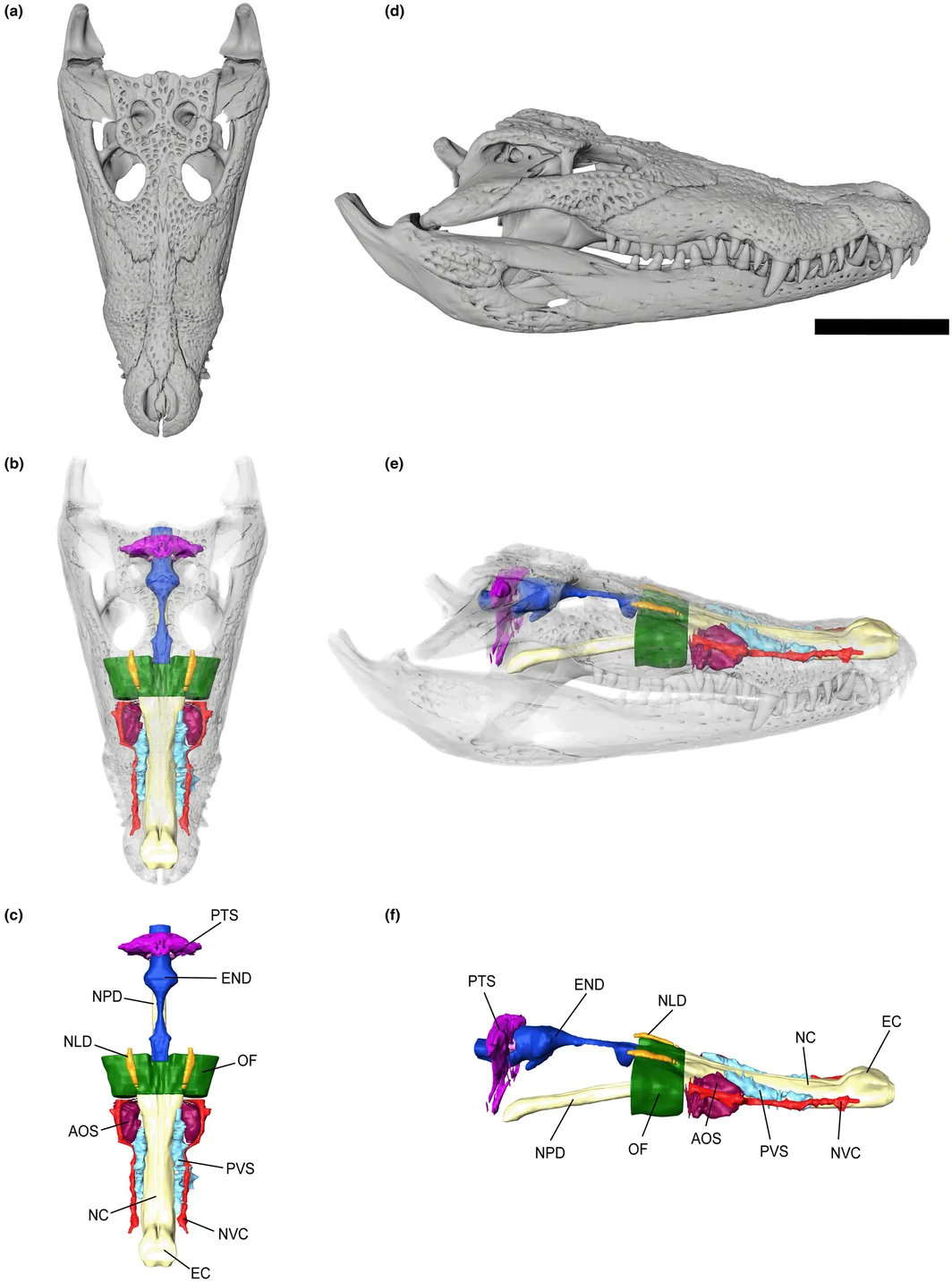
Skull of *Crocodylus moreletii* (TMM 4980). (a) skull rendering in dorsal view; (b) skull rendered transparent with internal anatomy visible in dorsal view; (c) internal anatomy of *Crocodylus moreletii* in dorsal view; (d) skull rendering in lateral view; (e) skull rendered transparent with internal anatomy visible in lateral view; (f) internal anatomy of *Crocodylus moreletii* in lateral view. AOS, antorbital sinus; EC, external choana; END, endocast; NC, nasal cavity; NLD, nasolacrimal duct; NPD, nasopharyngeal duct; NVC, neurovascular canal; OF, olfactory region; PTS, paratympanic sinus; PVS, postvestibular sinus. Scale bar = 10 cm.

**FIGURE 16:**
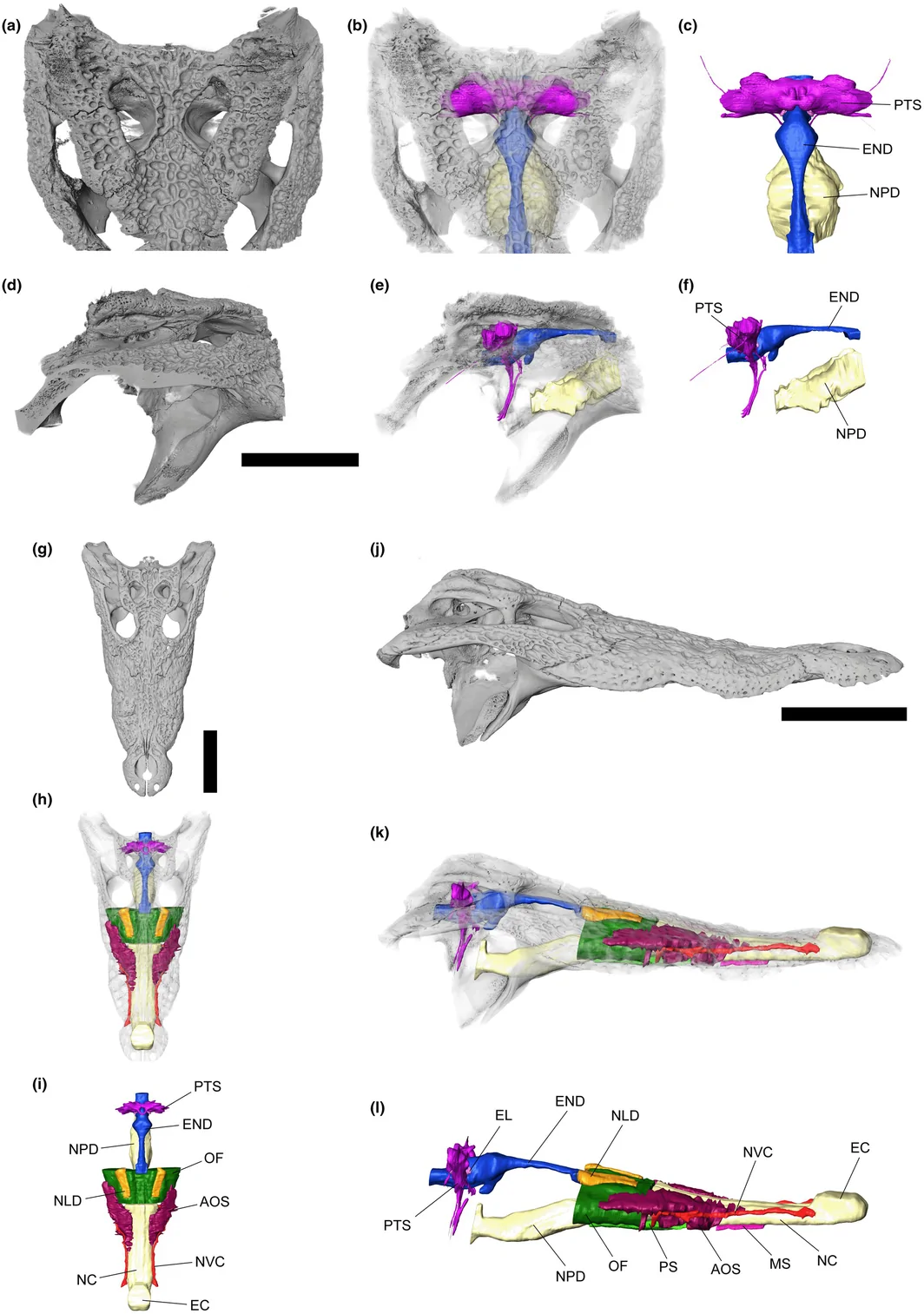
Skull of *Crocodylus niloticus* (MHNL 50001387). (a) skull rendering in dorsal view; (b) skull rendered transparent with internal anatomy visible in dorsal view; (c) internal anatomy of *Crocodylus niloticus* in dorsal view; (d) skull rendering in lateral view; (e) skull rendered transparent with internal anatomy visible in lateral view; (f) internal anatomy of *Crocodylus niloticus* in lateral view; Skull of *Crocodylus niloticus* (MZB 2003–1423) adapted from Serrano‐Martínez et al. (2021). (g) skull rendering in dorsal view; (h) skull rendered transparent with internal anatomy visible in dorsal view; (i) internal anatomy of *Crocodylus niloticus* in dorsal view; (j) skull rendering in lateral view; (k) skull rendered transparent with internal anatomy visible in lateral view; (l) internal anatomy of *Crocodylus niloticus* in lateral view. AOS, antorbital sinus; EC, external choana; EL, endosseous labyrinth; END, endocast; MS, maxillary sinus; NC, nasal cavity; NLD, nasolacrimal duct; NPD, nasopharyngeal duct; NVC, neurovascular canals; OF, olfactory region; PS, palatine sinus; PTS, paratympanic sinus. Scale bars = 10 cm.

**FIGURE 17:**
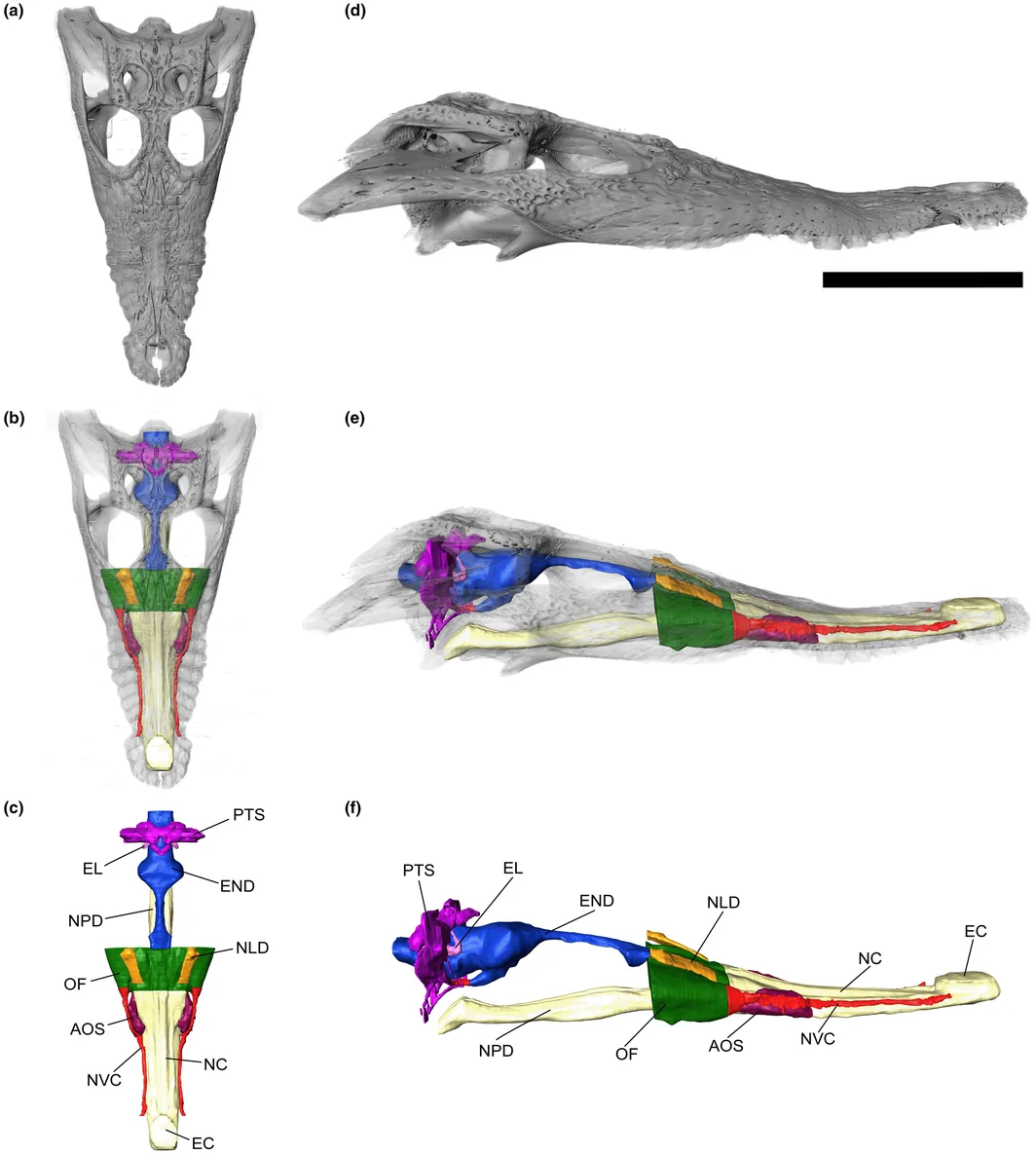
Skull of *Crocodylus novaeguineae* (DVZ M 9/13). (a) skull rendering in dorsal view; (b) skull rendered transparent with internal anatomy visible in dorsal view; (c) internal anatomy of *Crocodylus novaeguineae* in dorsal view; (d) skull rendering in lateral view; (e) skull rendered transparent with internal anatomy visible in lateral view; (f) internal anatomy of *Crocodylus novaeguineae* in lateral view. AOS, antorbital sinus; EC, external choana; EL, endosseous labyrinth; END, endocast; NC, nasal cavity; NLD, nasolacrimal duct; NPD, nasopharyngeal duct; NVC, neurovascular canals; OF, olfactory region; PTS, paratympanic sinus. Scale bar = 10 cm.

**FIGURE 18:**
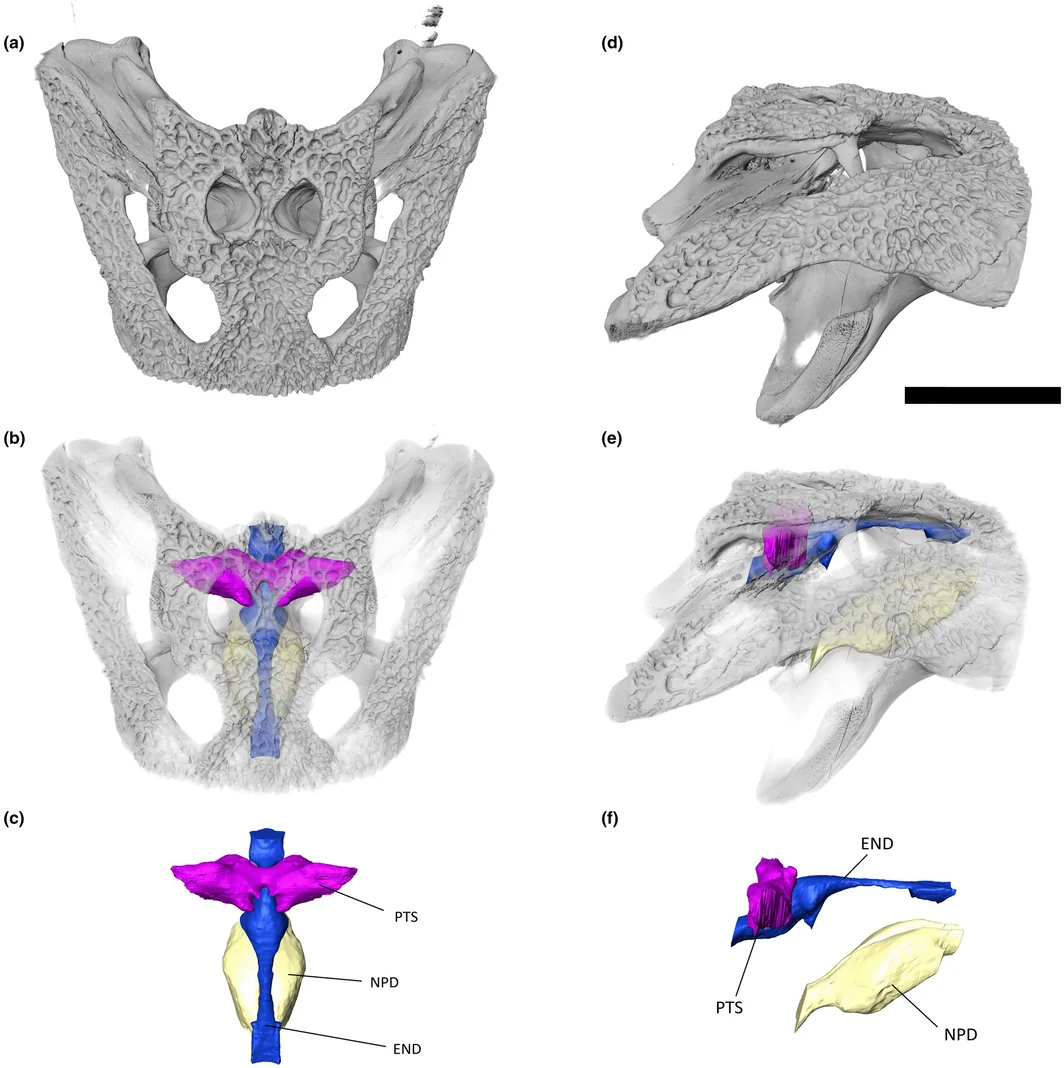
Skull of *Crocodylus palustris* (MHNL 50001398). (a) skull rendering in dorsal view; (b) skull rendered transparent with internal anatomy visible in dorsal view; (c) internal anatomy of *Crocodylus palustris* in dorsal view; (d) skull rendering in lateral view; (e) skull rendered transparent with internal anatomy visible in lateral view; (f) internal anatomy of *Crocodylus palustris* in lateral view. END, endocast; NPD, nasopharyngeal duct; PTS, paratympanic sinus. Scale bar = 5 cm.

**FIGURE 19:**
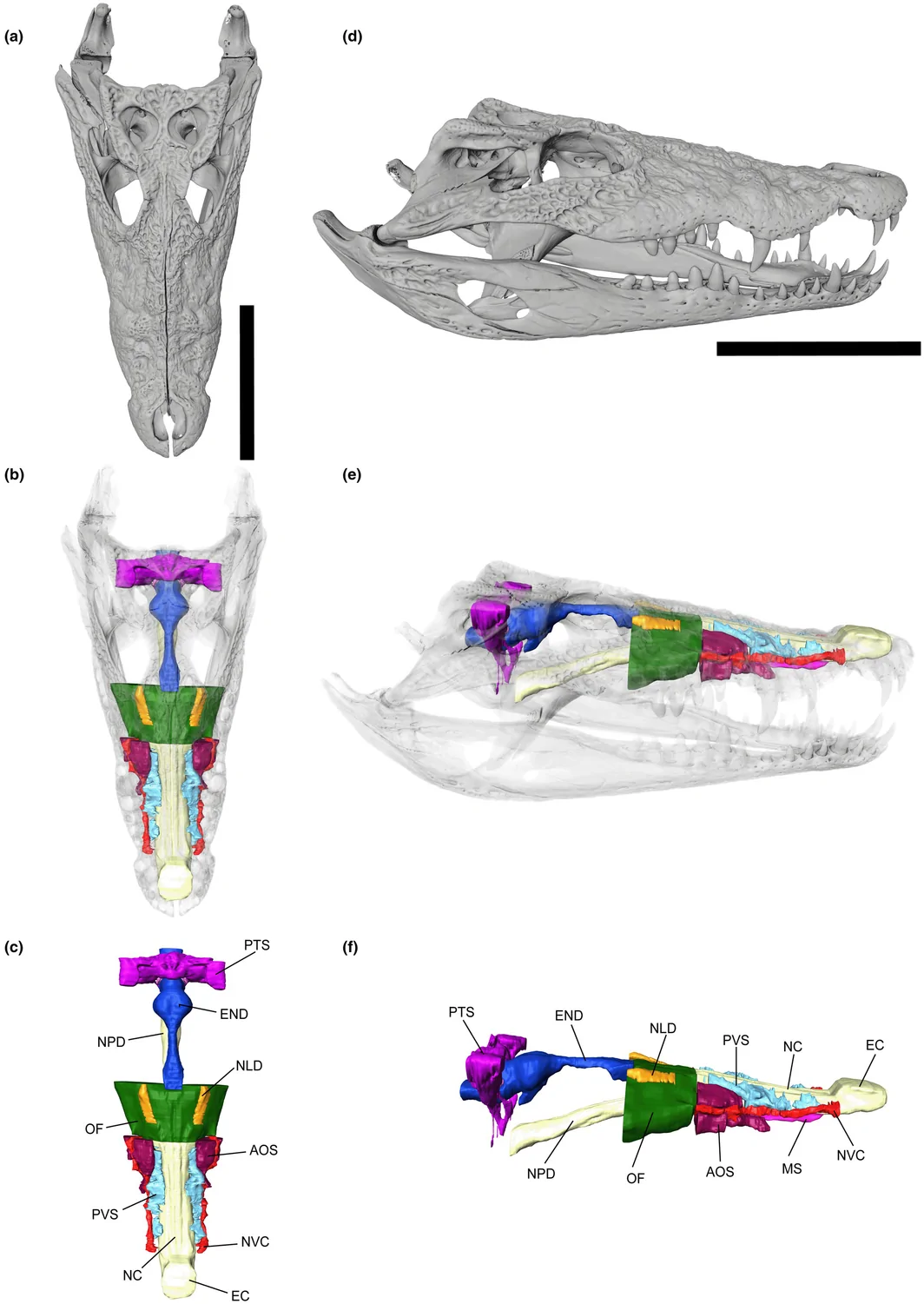
Skull of *Crocodylus rhombifer* (NMB.AB50.0171). (a) skull rendering in dorsal view; (b) skull rendered transparent with internal anatomy visible in dorsal view; (c) internal anatomy of *Crocodylus rhombifer* in dorsal view; (d) skull rendering in lateral view; (e) skull rendered transparent with internal anatomy visible in lateral view; (f) internal anatomy of *Crocodylus rhombifer* in lateral view. AOS, antorbital sinus; EC, external choana; END, endocast; MS, maxillary sinus; NC, nasal cavity; NLD, nasolacrimal duct; NPD, nasopharyngeal duct; NVC, neurovascular canals; OF, olfactory region; PTS, paratympanic sinus; PVS, postvestibular sinus. Scale bars = 10 cm.

**FIGURE 20:**
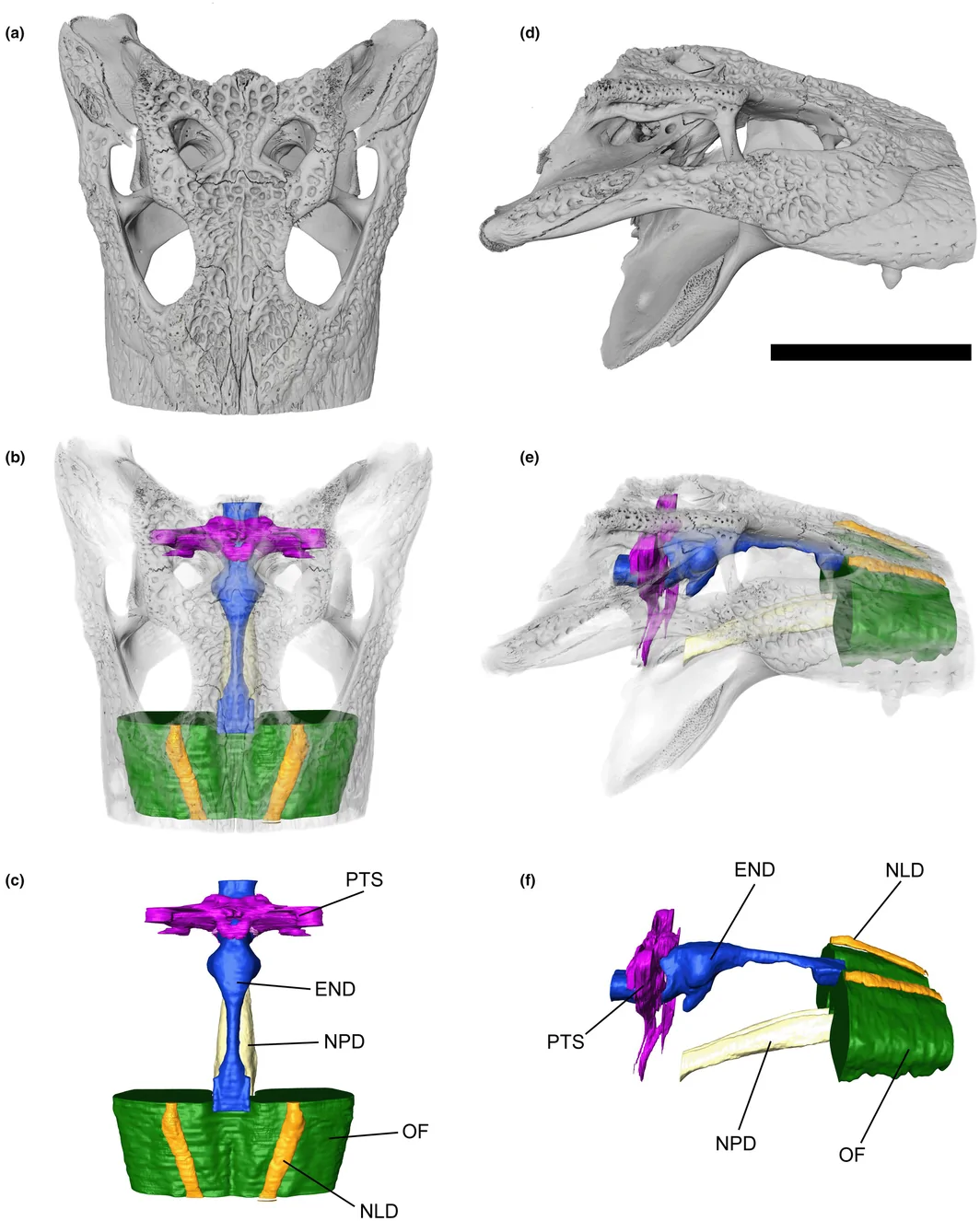
Skull of *Crocodylus siamensis* (MHNL 50001389). (a) skull rendering in dorsal view; (b) skull rendered transparent with internal anatomy visible in dorsal view; (c) internal anatomy of *Crocodylus siamensis* in dorsal view; (d) skull rendering in lateral view; (e) skull rendered transparent with internal anatomy visible in lateral view; (f) internal anatomy of *Crocodylus siamensis* in lateral view. END, endocast; NLD, nasolacrimal duct; NPD, nasopharyngeal duct; OF, olfactory region; PTS, paratympanic sinus. Scale bars = 10 cm.

**FIGURE 21:**
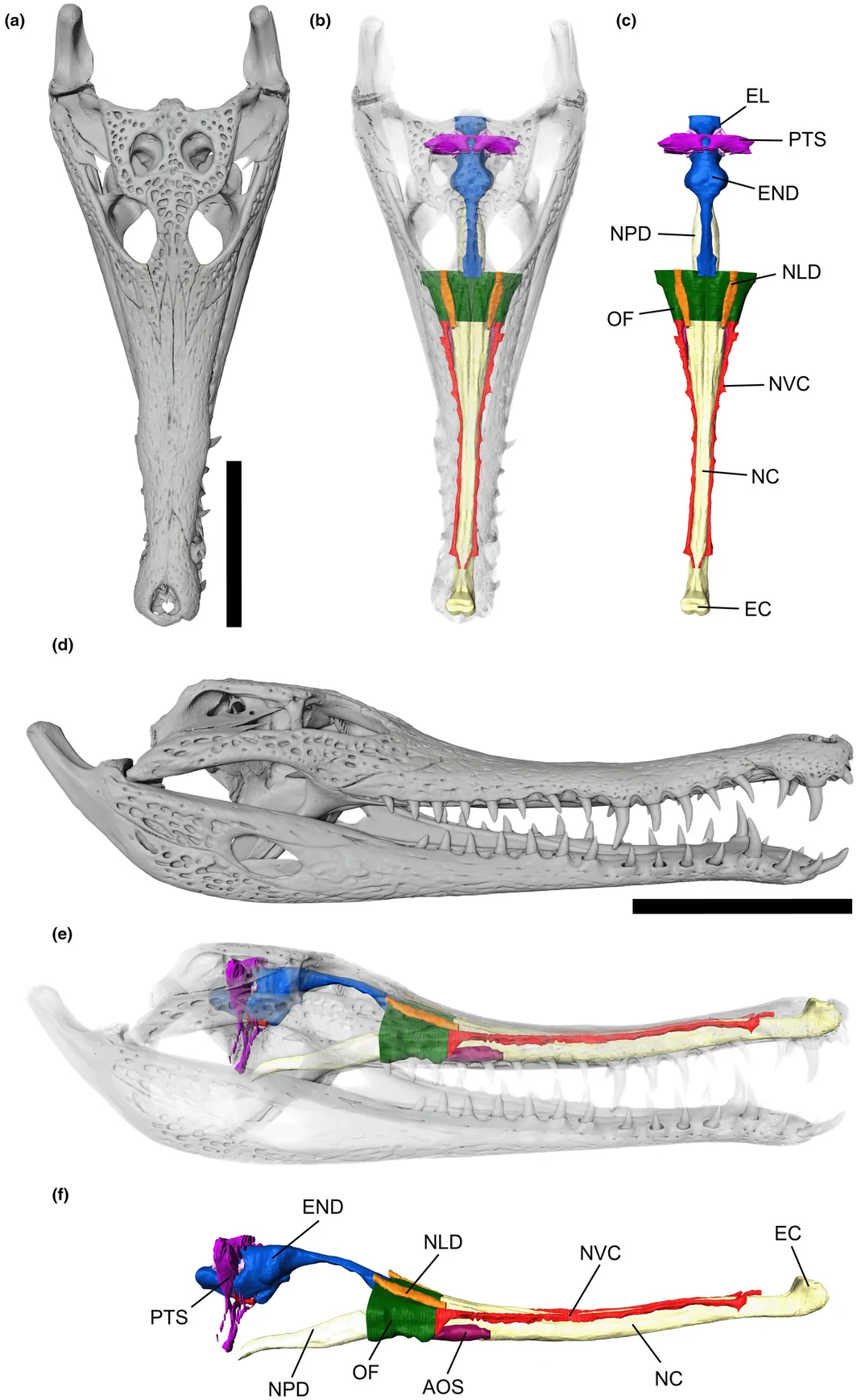
Skull of *Mecistops cataphractus* (TMM M 3529). (a) skull rendering in dorsal view; (b) skull rendered transparent with internal anatomy visible in dorsal view; (c) internal anatomy of *Mecistops cataphractus* in dorsal view; (d) skull rendering in lateral view; (e) skull rendered transparent with internal anatomy visible in lateral view; (f) internal anatomy of *Mecistops cataphractus* in lateral view. AOS, antorbital sinus; EC, external choana; EL, endosseous labyrinth; END, endocast; NC, nasal cavity; NLD, nasolacrimal duct; NPD, nasopharyngeal duct; NVC, neurovascular canals; OF, olfactory region; PTS, paratympanic sinus. Scale bars = 10 cm.

**FIGURE 22:**
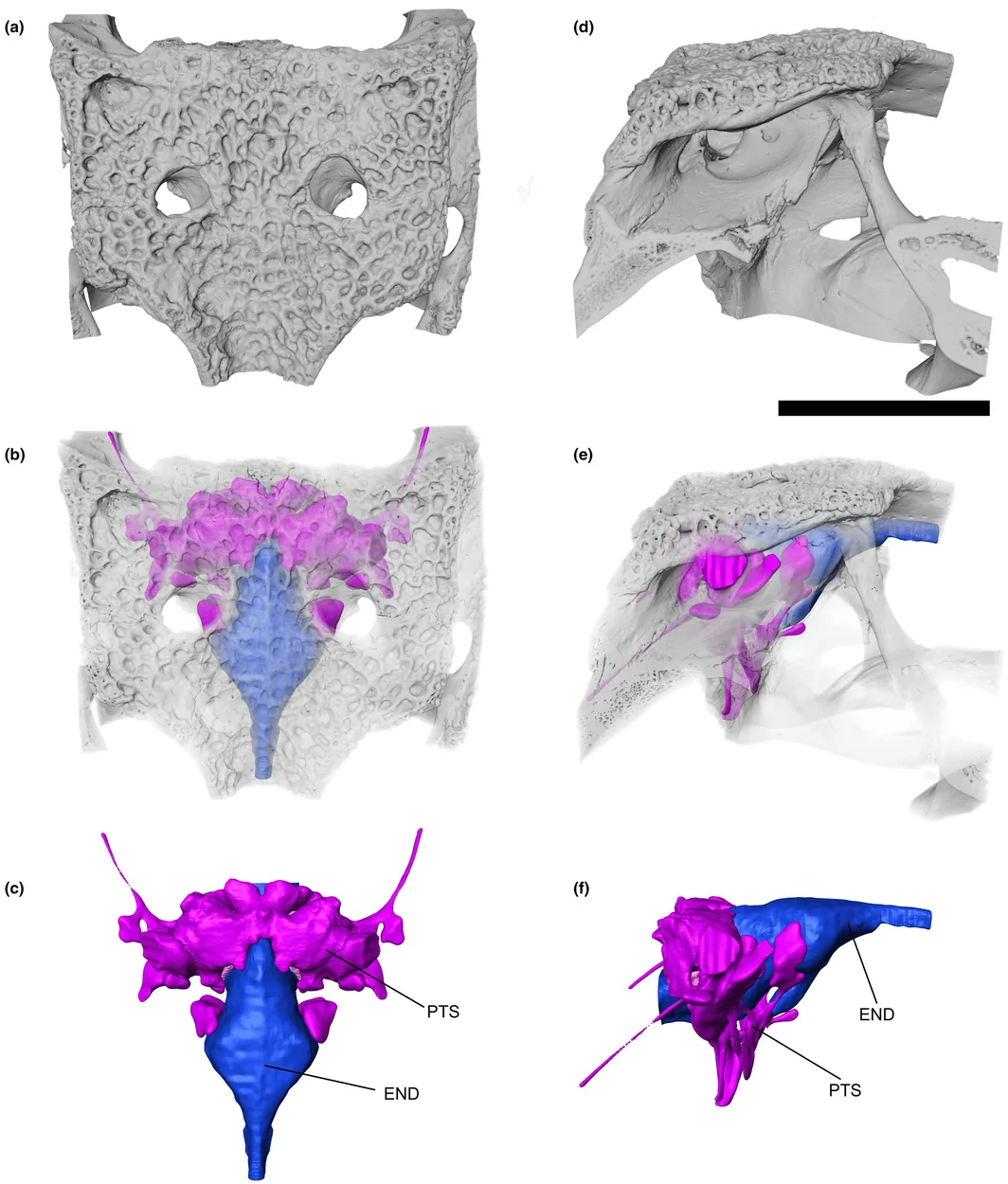
Skull of *Osteolaemus tetraspis* (UCBL 2019–1‐236). (a) skull rendering in dorsal view; (b) skull rendered transparent with internal anatomy visible in dorsal view; (c) internal anatomy of *Osteolaemus tetraspis* in dorsal view; (d) skull rendering in lateral view; (e) skull rendered transparent in lateral view; (f) internal anatomy of *Osteolaemus tetraspis* in lateral view. END, endocast; PTS, paratympanic sinus. Scale bar = 5 cm.

**FIGURE 23:**
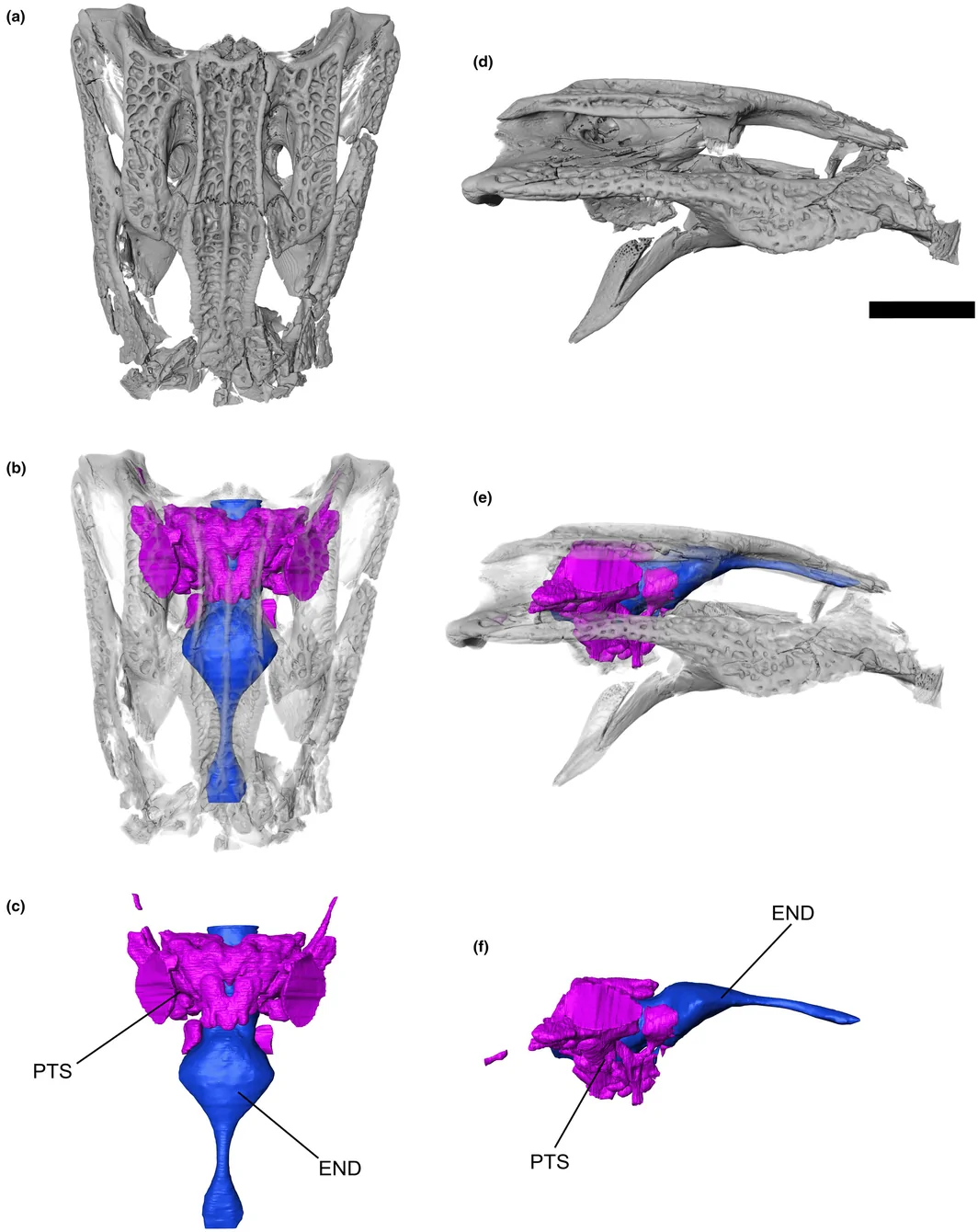
Skull of *Trilophosuchus rackhami* (QMF 16856) adapted from Ristevski (2022). (a) skull rendering in dorsal view; (b) skull rendered transparent with internal anatomy visible in dorsal view; (c) internal anatomy of *Trilophosuchus rackhami* in dorsal view; (d) skull rendering in lateral view; (e) skull rendered transparent in lateral view; (f) internal anatomy of *Trilophosuchus rackhami* in lateral view. END, endocast; PTS, paratympanic sinus. Scale bar = 2 cm.

**FIGURE 24:**
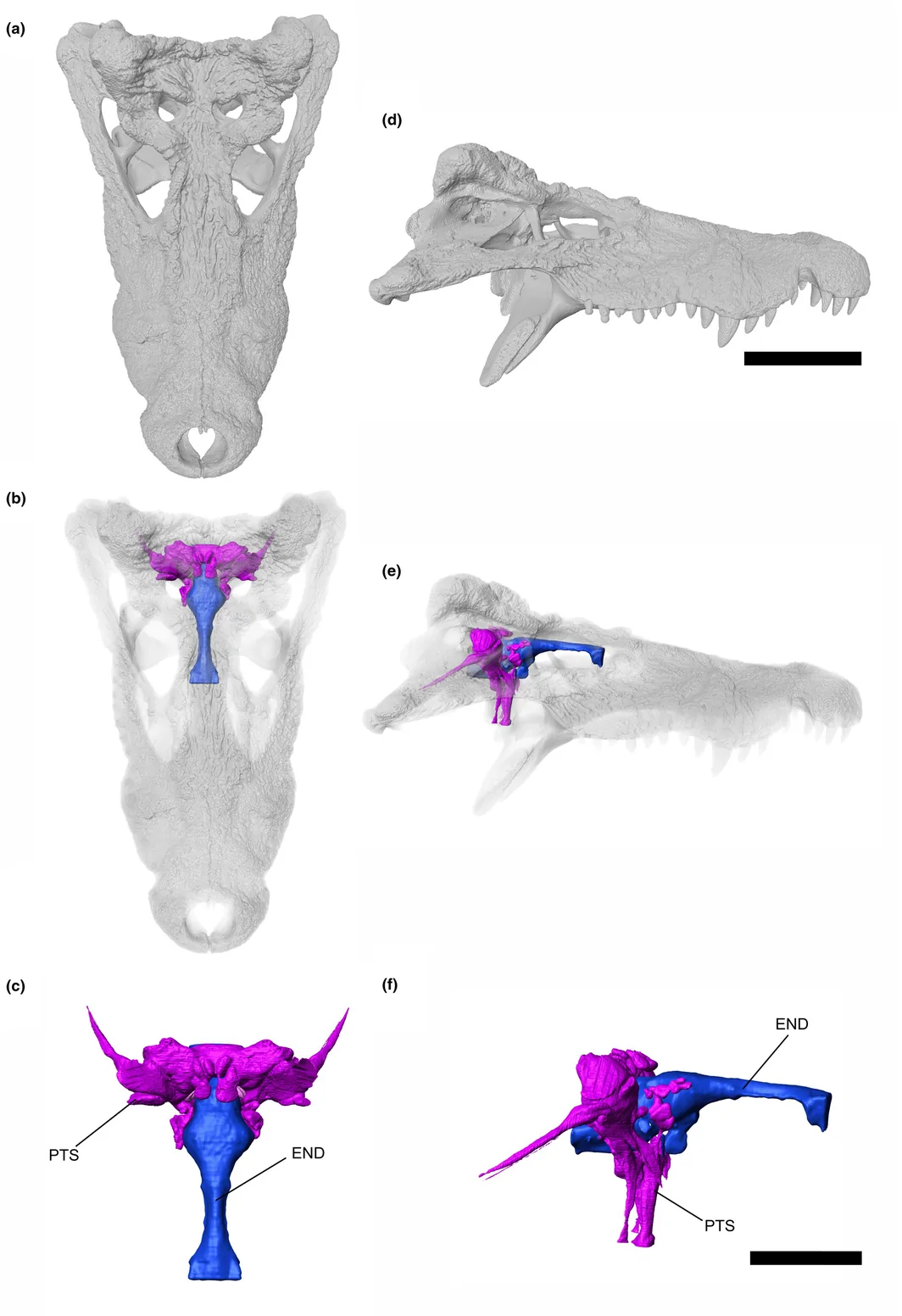
Skull of *Voay robustus* (NHMUK PV R 36685) adapted from Perrichon et al. (2024). (a) skull rendering in dorsal view; (b) skull rendered transparent with internal anatomy visible in dorsal view; (c) internal anatomy of *Voay robustus* in dorsal view; (d) skull rendering in lateral view; (e) skull rendered transparent in lateral view; (f) internal anatomy of *Voay robustus* in lateral view. END, endocast; PTS, paratympanic sinus. Scale bars = 10 cm (d), 5 cm (f).

Amongst Crocodylia, the encephalic endocasts of Gavialoidea are the straightest in lateral view (Figures 25, 26, 27, 28, 29, 30, 31, 32, 33, 34, 35, 36, 37, 38, 39; Table 2). *Argochampsa krebsi* shows the greatest degree of curvature within Gavialoidea, with *Eothoracosaurus mississippiensis* displaying the least curvature in this clade (Pligersdorffer et al., 2025; Boerman et al., in review; Figure 25f; Figure 28f; Table 2). Although the high cephalic and pontine flexure angles in *Eosuchus lerichei* might be accentuated by artefactual dorsoventral compression of the skull table (Figure 27; Burke et al., 2025), thoracosaurs, in general, are characterised by high values (Boerman et al., in review; Table 2). The cerebrum has its greatest transverse width at its most posterior point in gavialoids, narrowing anteriorly, as is the case in non‐crocodylian eusuchians, Alligatoroidea, and in some crocodyloids. Burke and Mannion (2023) noted that the greatest transverse width of the cerebrum in *Tomistoma schlegelii* (Figure 39i) is at its midlength; however, this appears to reflect the immature ontogenetic status of the studied specimen (TMM M6342), as the cerebrum of adult *Tomistoma schlegelii* has its greatest expansion at the posterior end, narrowing anteriorly (Figure 39c). The transverse width of the cerebrum of gavialoids is relatively small compared to that of the skull table, with most species displaying ratios of 0.3 or lower, except for *Argochampsa krebsi* and *Tomistoma schlegelii* (Table 2). Thoracosaurs and *Gavialis* display the smallest values (Table 2). As in Crocodyloidea, the anteroposterior length of the olfactory bulb is less than 30% that of the olfactory tract in Gavialoidea. In several gavialoids, including *Eogavialis gavialoides* (Figure 26c), *Eothoracosaurus mississippiensis* (Figure 28c), *Gavialis gangeticus* (Figure 29c), *Gavialosuchus eggenburgensis* (Figure 30c), *Thecachampsa americana* (Figure 36c) and ‘*Tomistoma’ cairense* (Figure 38c), the olfactory bulb is difficult to distinguish from the olfactory tract in dorsal and ventral views as there is little to no difference in transverse width between them. The bulb can be distinguished in lateral view in some of these species though, including *Thecachampsa americana* (Figure 36f) and ‘*Tomistoma*’ *cairense* (Figure 38f), wherein the bulb increases in dorsoventral height relative to the tract.

**FIGURE 25:**
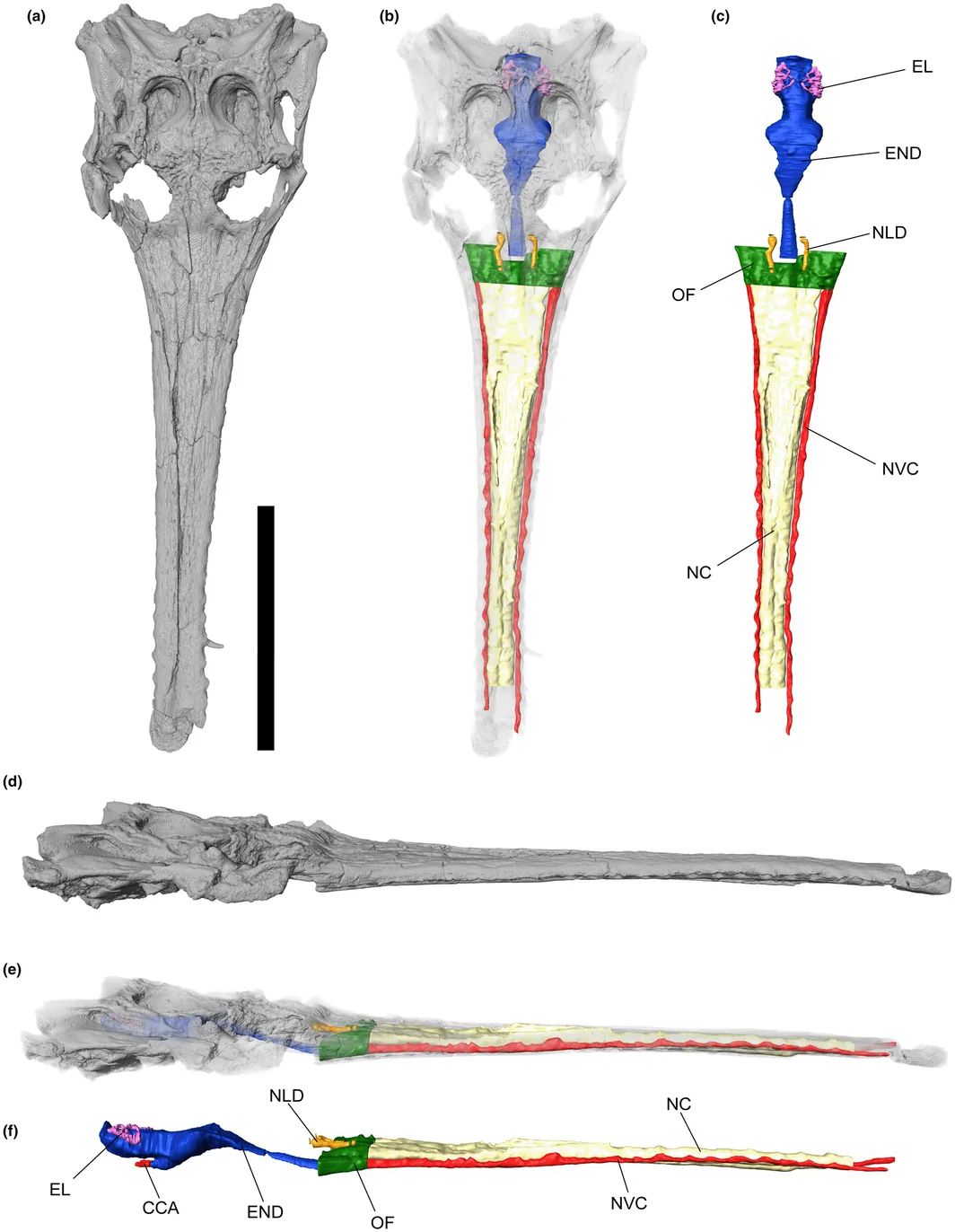
Skull of *Argochampsa krebsi* (NHMUK PV R36872) adapted from Pligersdorffer et al. (2025). (a) skull rendering in dorsal view; (b) skull rendered transparent with internal anatomy visible in dorsal view; (c) internal anatomy of *Argochampsa krebsi* in dorsal view; (d) skull rendering in lateral view; (e) skull rendered transparent with internal anatomy visible in lateral view; (f) internal cranial anatomy of *Argochampsa krebsi* in lateral view. CCA, cerebral carotid arteries; EL, endosseous labyrinth; END, endosseous labyrinth; NC, nasal cavity; NLD, nasolacrimal duct; NVC, neurovascular canal; OF, olfactory region. Scale bar = 10 cm.

**FIGURE 26:**
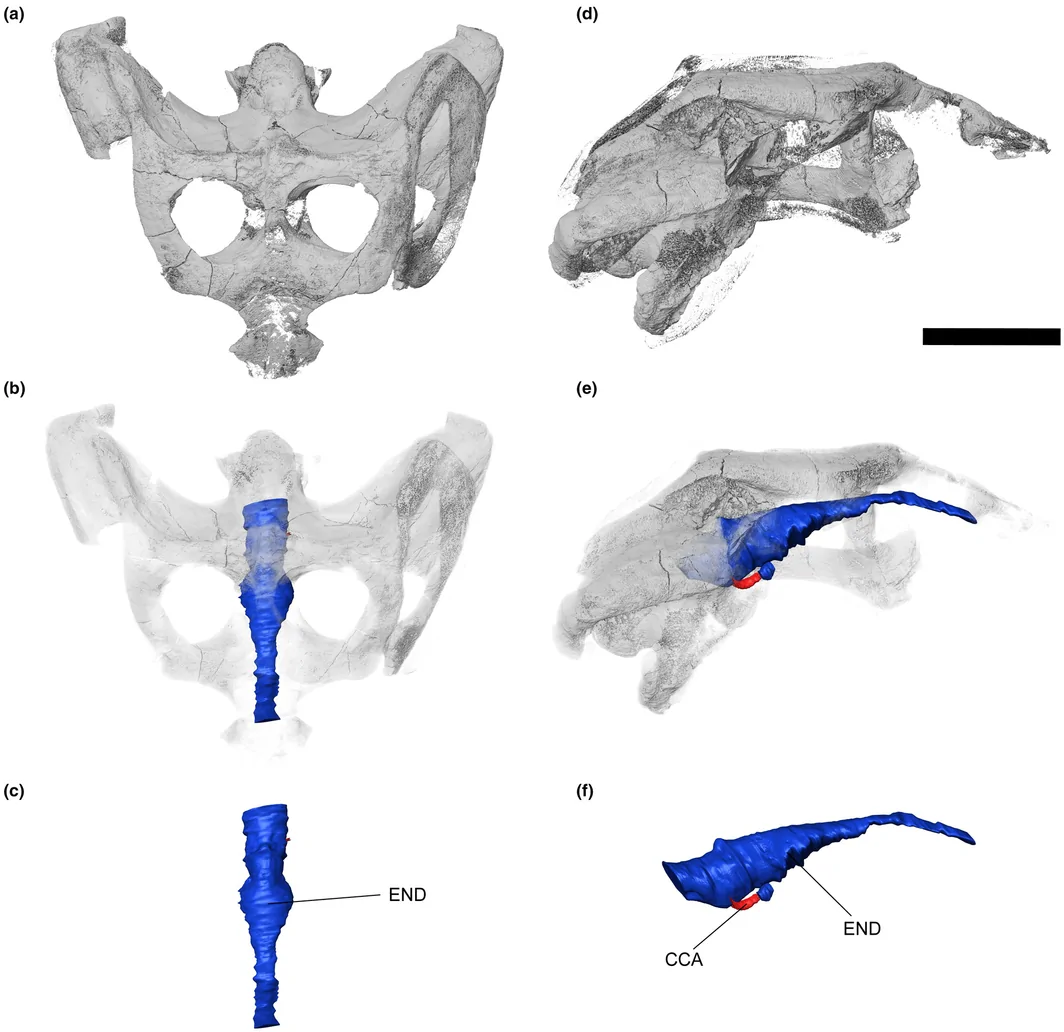
Skull of *Eogavialis gavialoides* (NHMUK R3325). (a) skull rendering in dorsal view; (b) skull rendered transparent with internal anatomy visible in dorsal view; (c) internal anatomy of *Eogavialis gavialoides* in dorsal view; (d) skull rendering in lateral view; (e) skull rendered transparent with internal anatomy visible in lateral view; (f) internal cranial anatomy of *Eogavialis gavialoides* in lateral view. CCA, cerebral carotid arteries; END, endocast. Scale bar = 10 cm.

**FIGURE 27:**
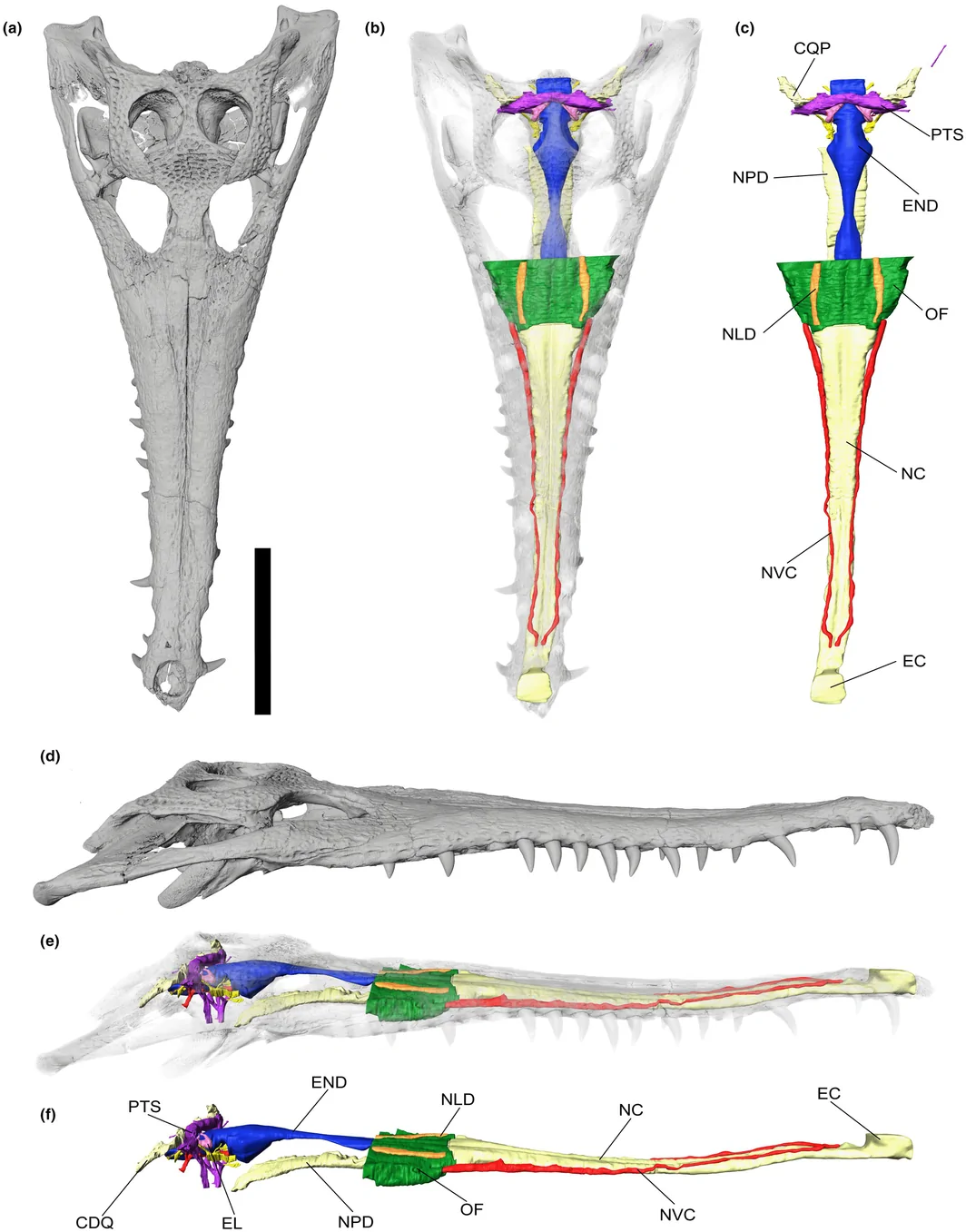
Skull of *Eosuchus lerichei* (IRSNB R49). (a) skull rendering in dorsal view; (b) skull rendered transparent with internal anatomy visible in dorsal view; (c) internal anatomy of *Eosuchus lerichei* in dorsal view; (d) skull rendering in lateral view; (e) skull rendered transparent with internal anatomy visible in lateral view; (f) internal anatomy of *Eosuchus lerichei* in lateral view. CQP, cranioquadrate passage; EC, external choana; EL, endosseous labyrinth; END, endocast; NC, nasal cavity; NLD, nasolacrimal duct; NPD, nasopharyngeal duct; NVC, neurovascular canals; OF, olfactory region; PTS, paratympanic sinus. Scale bar = 10 cm.

**FIGURE 28:**
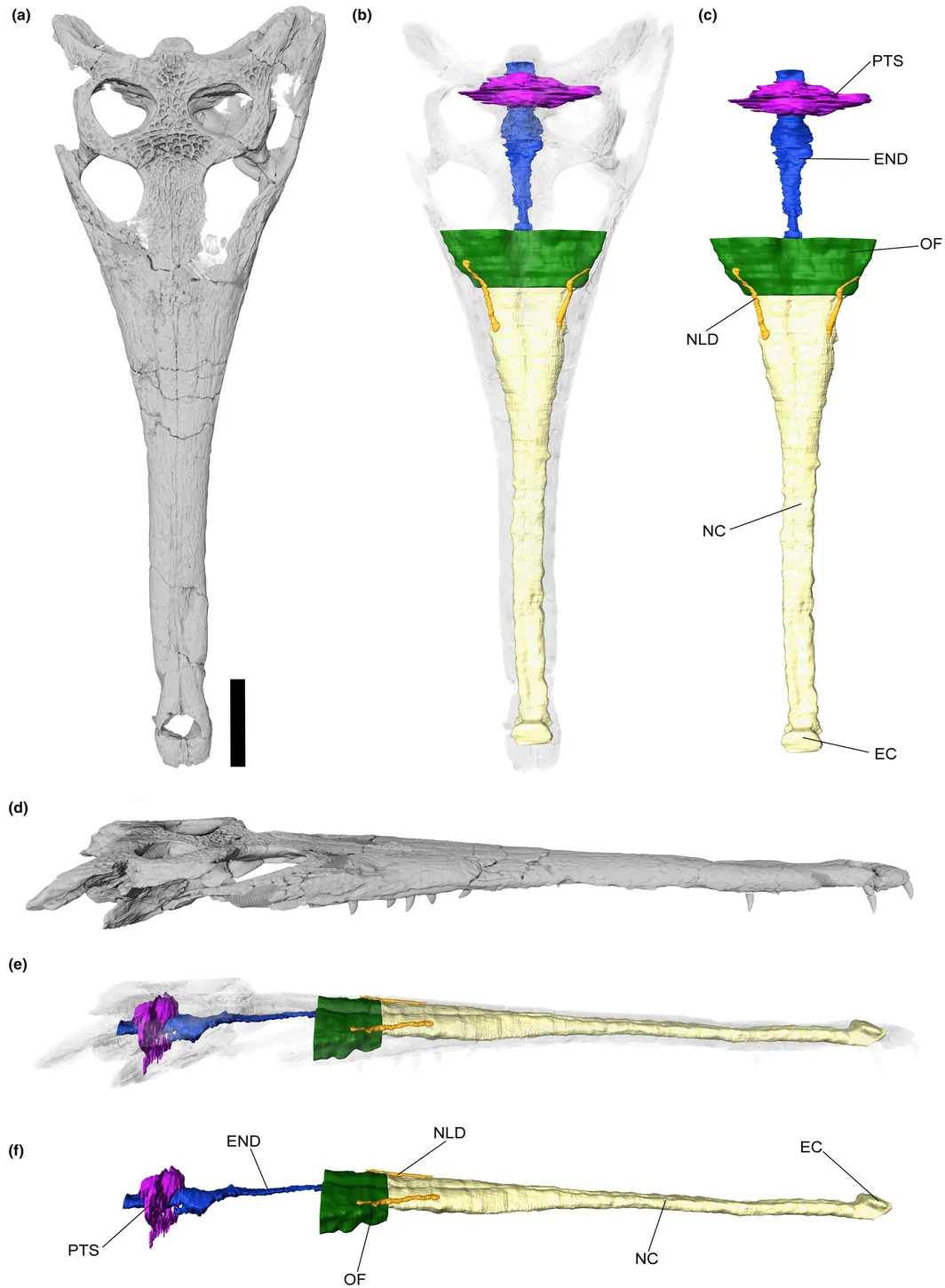
Skull of *Eothoracosaurus mississippiensis* (DSM 3293) adapted from Boerman et al. (in review). (a) skull rendering in dorsal view; (b) skull rendered transparent with internal anatomy visible in dorsal view; (c) internal anatomy of *Eothoracosaurus mississippiensis* in dorsal view; (d) skull rendering in lateral view; (e) skull rendered transparent with internal anatomy visible in lateral view; (f) internal anatomy of *Eothoracosaurus mississippiensis* in lateral view. EC, external choana; END, endocast; NC, nasal cavity; NLD, nasolacrimal duct; OF, olfactory region; PTS, paratympanic sinus. Scale bar = 10 cm.

**FIGURE 29:**
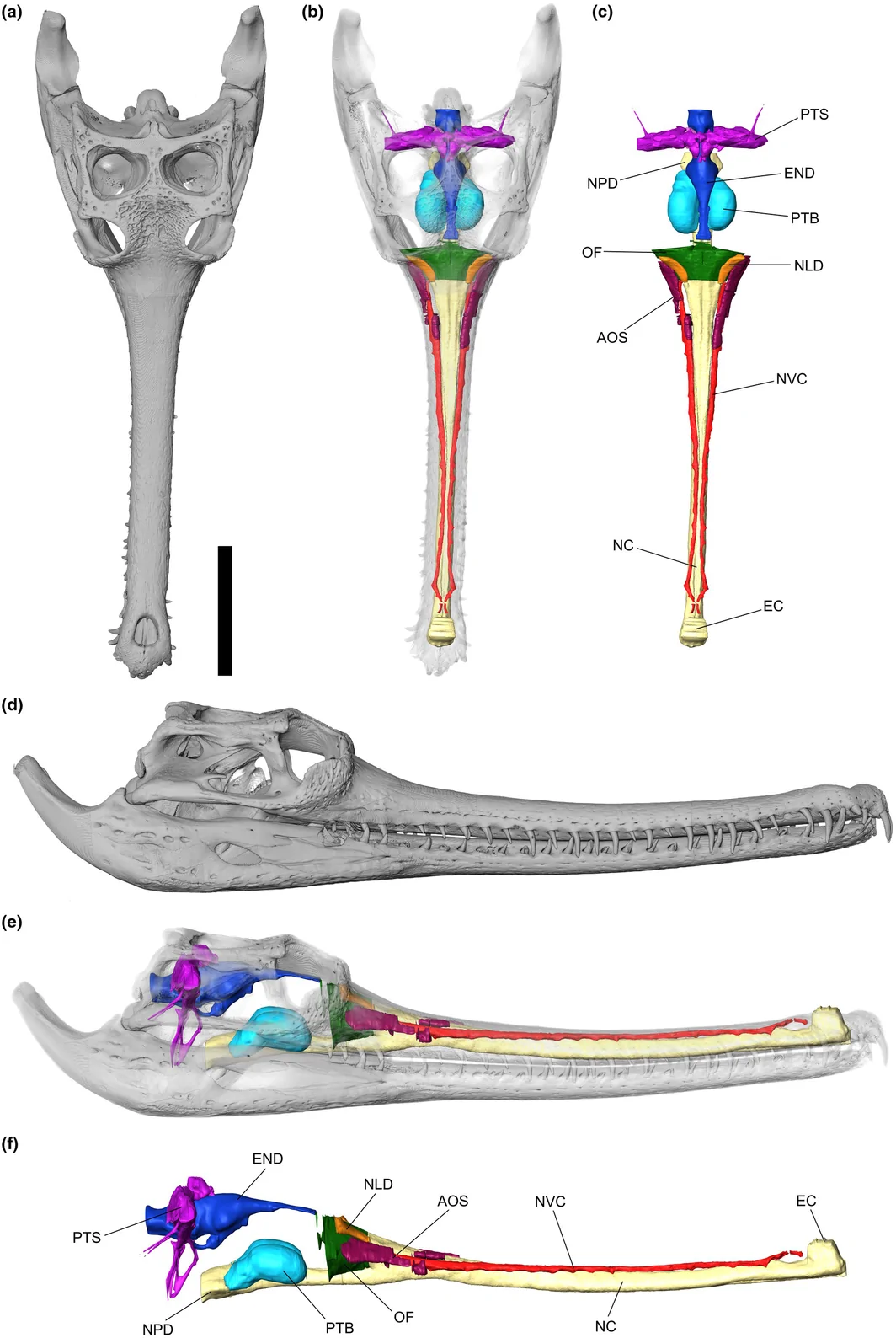
Skull of *Gavialis gangeticus* (FLMNH UF 118998). (a) skull rendering in dorsal view; (b) skull rendered transparent with internal anatomy visible in dorsal view; (c) internal anatomy of *Gavialis gangeticus* in dorsal view; (d) skull rendering in lateral view; (e) skull rendered transparent with internal anatomy visible in lateral view; (f) internal anatomy of *Gavialis gangeticus* in lateral view. AOS, antorbital sinus; EC, external choana; END, endocast; NC, nasal cavity; NLD, nasolacrimal duct; NPD; nasopharyngeal duct; NVC, neurovascular canals; OF, olfactory region; PTB, pterygoid bulla; PTS, paratympanic sinus. Scale bar = 10 cm.

**FIGURE 30:**
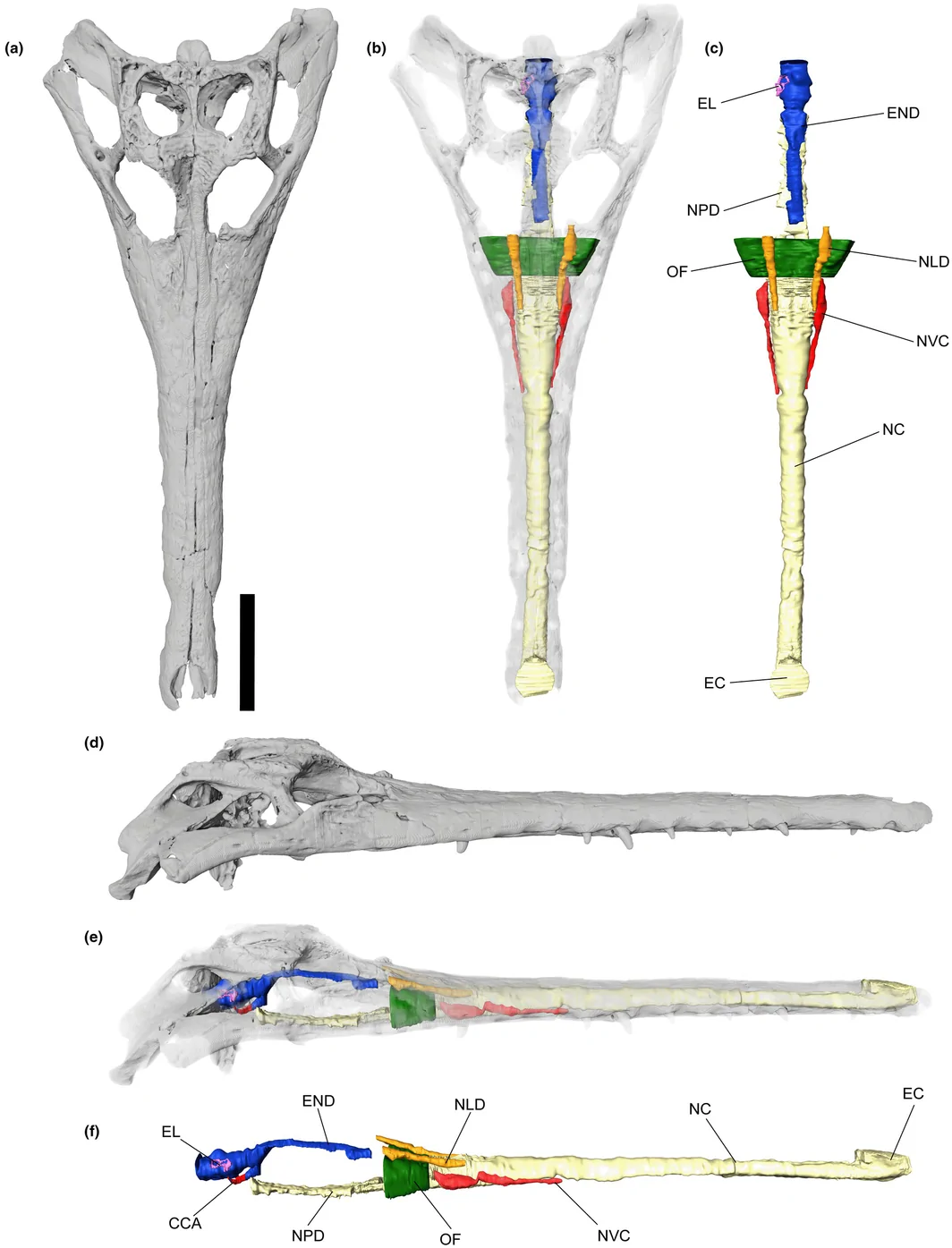
Skull of *Gavialosuchus eggenburgensis*. (a) skull rendering in dorsal view; (b) skull rendered transparent with internal anatomy visible in dorsal view; (c) internal anatomy of *Gavialosuchus eggenburgensis* in dorsal view; (d) skull rendering in lateral view; (e) skull rendered transparent with internal anatomy visible in lateral view; (f) internal anatomy of *Gavialosuchus eggenburgensis* in lateral view. CCA, cephalic carotid arteries; EC, external choana; EL, endosseous labyrinth; END, endocast; NC, nasal cavity; NLD, nasolacrimal duct; NPD; nasopharyngeal duct; NVC, neurovascular canals; OF, olfactory region. Scale bar = 10 cm.

**FIGURE 31:**
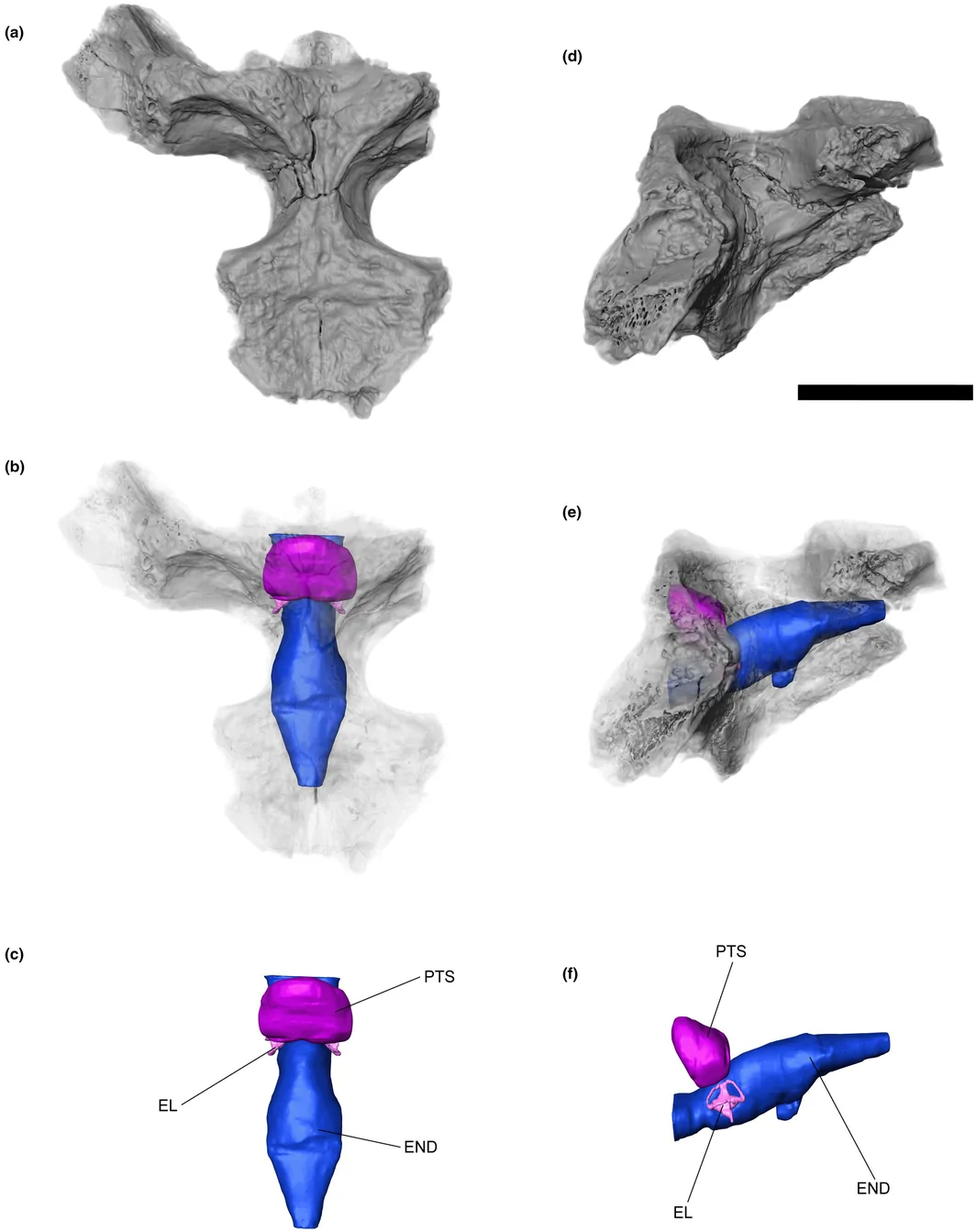
Skull of *Gunggamarandu maunala* (QMF 14.548) adapted from Ristevski et al. (2021). (a) skull rendering in dorsal view; (b) skull rendered transparent with internal anatomy visible in dorsal view; (c) internal anatomy of *Gunggamarandu maunala* in dorsal view; (d) skull rendering in lateral view; (e) skull rendered transparent with internal anatomy visible in lateral view; (f) internal anatomy of *Gunggamarandu maunala* in lateral view. EL, endosseous labyrinth; END, endocast; PTS, paratympanic sinus. Scale bar = 10 cm.

**FIGURE 32:**
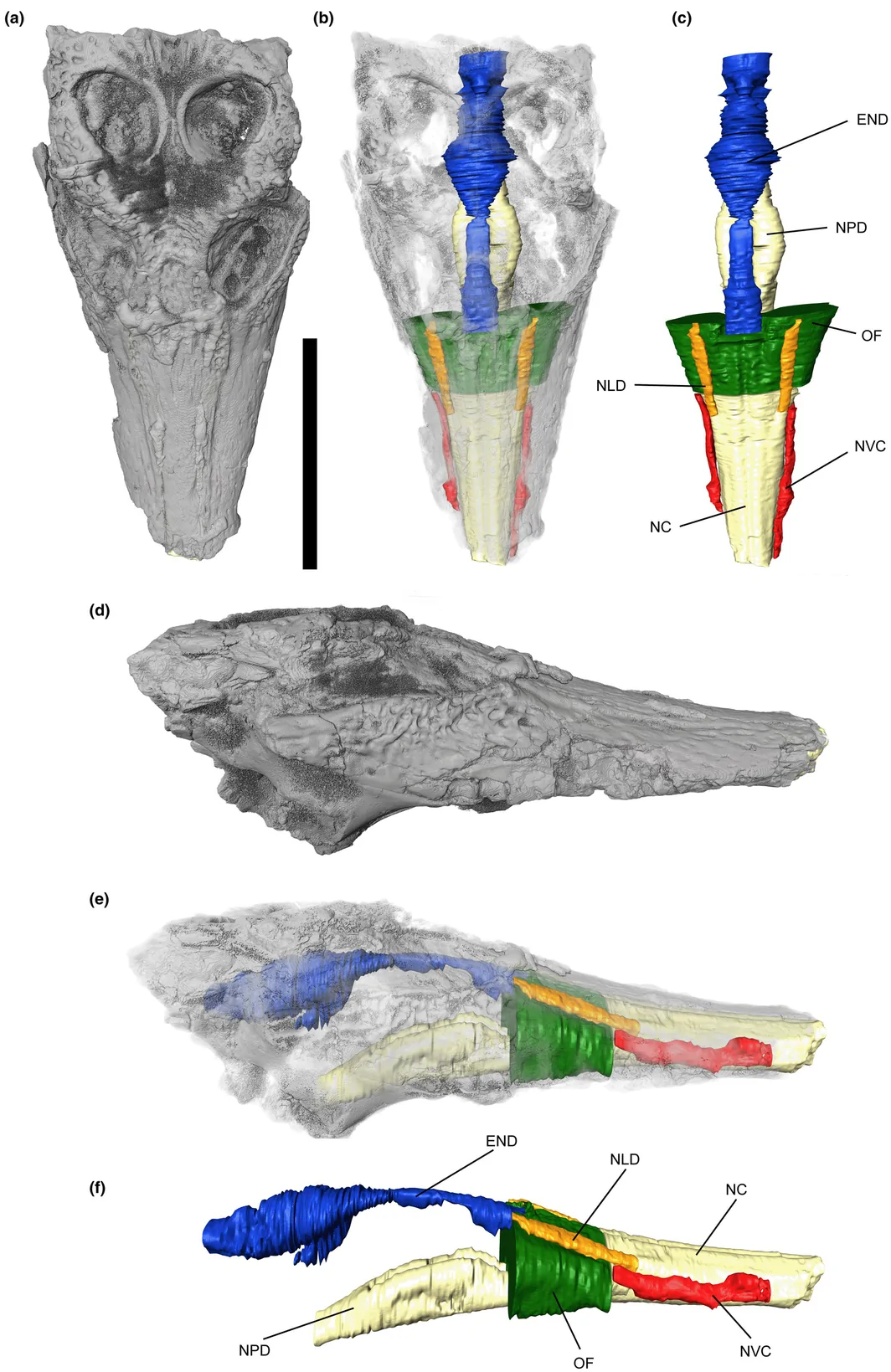
Skull of *Kentisuchus spenceri* (NHMUK R19633). (a) skull rendering in dorsal view; (b) skull rendered transparent with internal anatomy visible in dorsal view; (c) internal anatomy of *Kentisuchus spenceri* in dorsal view; (d) skull rendering in lateral view; (e) skull rendered transparent with internal anatomy visible in lateral view; (f) internal anatomy of *Kentisuchus spenceri* in lateral view. END, endocast; NC, nasal cavity; NLD, nasolacrimal duct; NPD, nasopharyngeal duct; NVC, neurovascular canal; OF, olfactory region. Scale bar = 10 cm.

**FIGURE 33:**
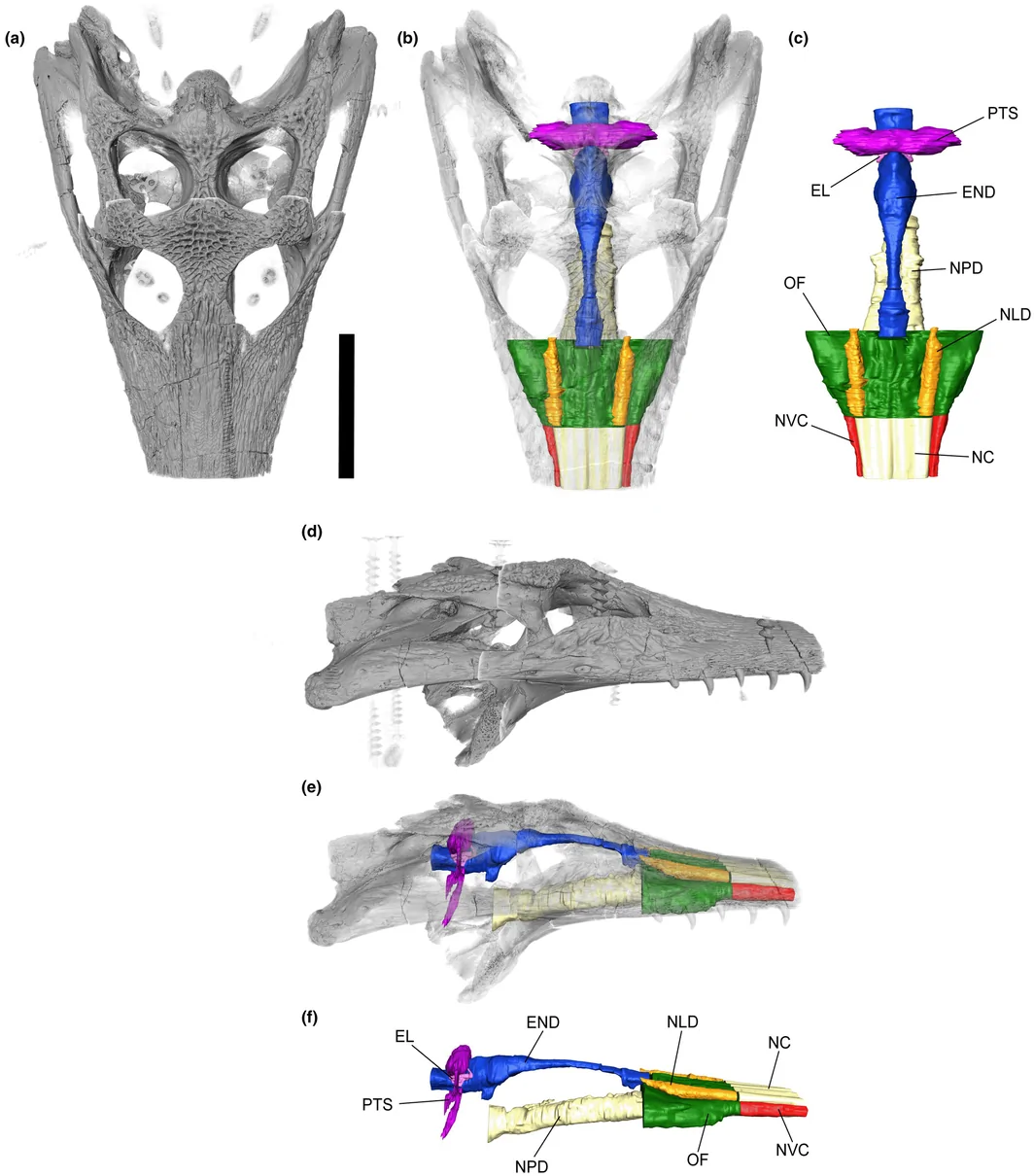
Skull of *Piscogavialis jugaliperforatus* (SMNK 1282). (a) skull rendering in dorsal view; (b) skull rendered transparent with internal anatomy visible in dorsal view; (c) internal anatomy of *Piscogavialis jugaliperforatus* in dorsal view; (d) skull rendering in lateral view; (e) skull rendered transparent with internal anatomy visible in lateral view; (f) internal anatomy of *Piscogavialis jugaliperforatus* in lateral view. EL, endosseous labyrinth; END, endocast; NC, nasal cavity; NLD, nasolacrimal duct; NPD, nasopharyngeal duct; NVC, neurovascular canal; OF, olfactory region; PTS, paratympanic sinus. Scale bar = 10 cm.

**FIGURE 34:**
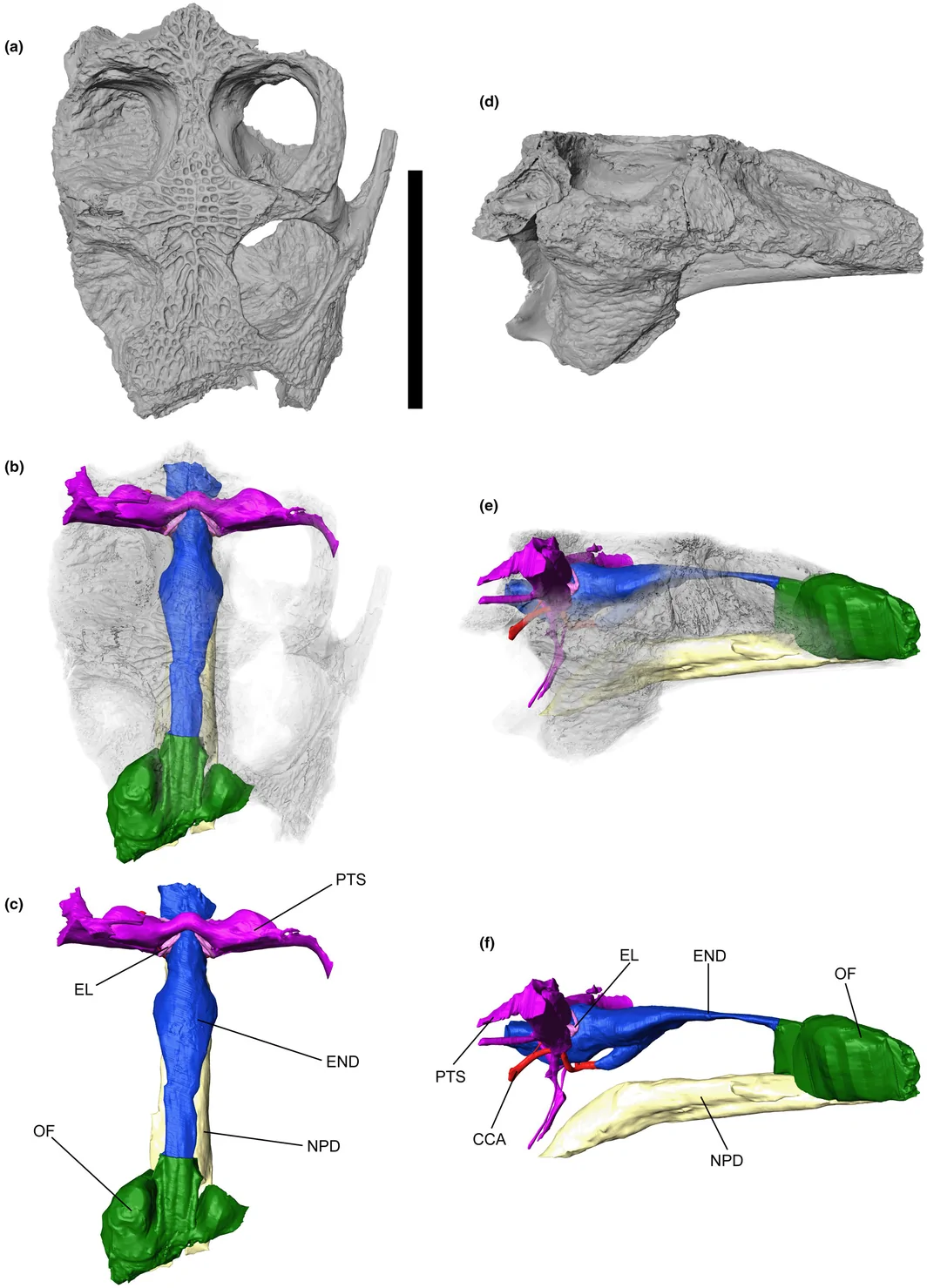
Skull of *Portugalosuchus azenhae* (ML1818) adapted from Puértolas‐Pascual et al. (2023). (a) skull rendering in dorsal view; (b) skull rendered transparent with internal anatomy visible in dorsal view; (c) internal anatomy of *Portugalosuchus azenhae* in dorsal view; (d) skull rendering in lateral view; (e) skull rendered transparent with internal anatomy visible in lateral view; (f) internal anatomy of *Portugalosuchus azenhae* in lateral view. CCA, cerebral carotid arteries; EL, endosseous labyrinth; END, endocast; NPD, nasopharyngeal duct; OF, olfactory region; PTS, paratympanic sinus. Scale bar = 10 cm.

**FIGURE 35:**
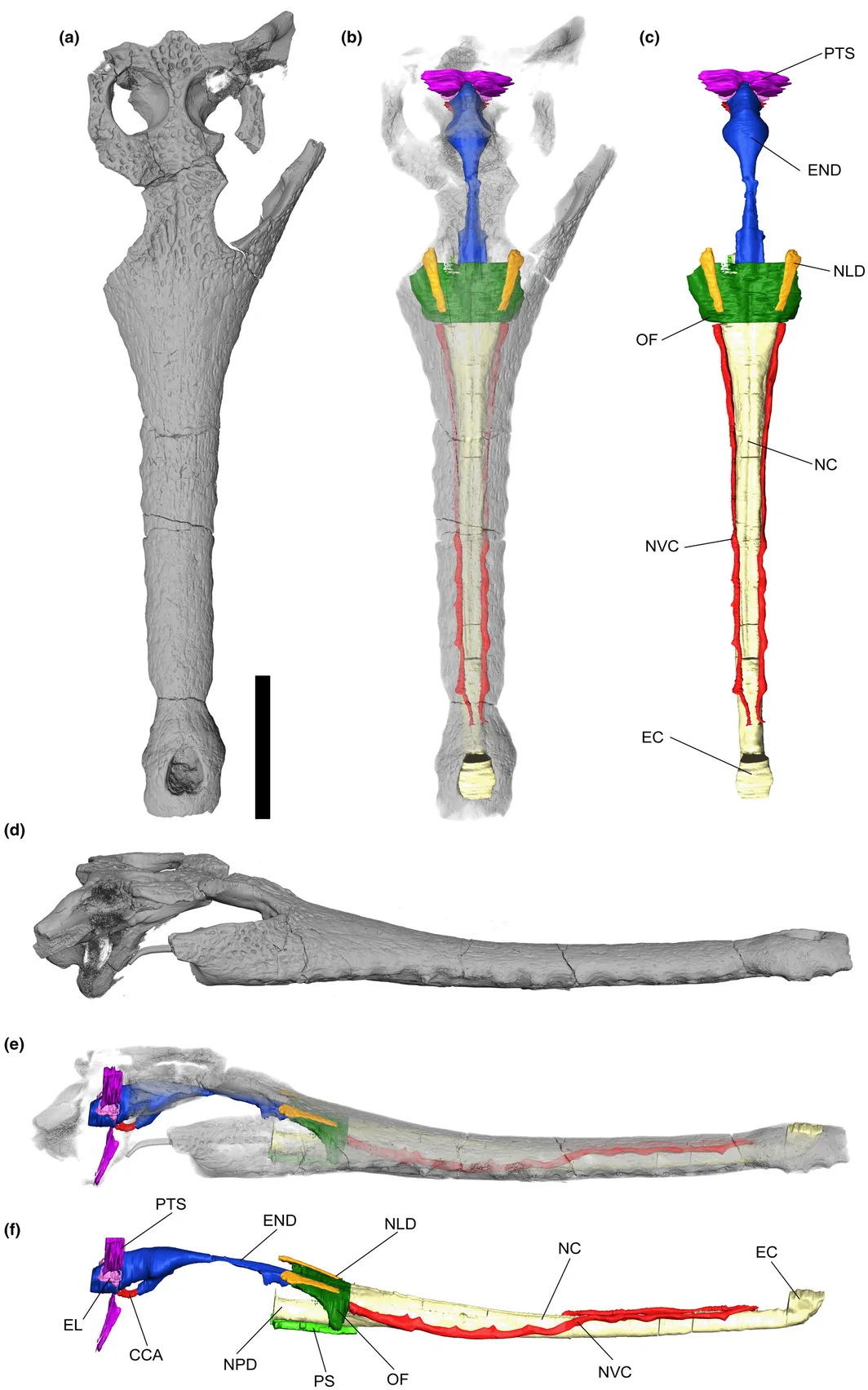
Skull of *Sutekhsuchus dowsoni* (NHMUK PV R4769) adapted from Burke and Mannion (2023). (a) skull rendering in dorsal view; (b) skull rendered transparent with internal anatomy visible in dorsal view; (c) internal anatomy of *Sutekhsuchus dowsoni* in dorsal view; (d) skull rendering in lateral view; (e) skull rendered transparent with internal anatomy visible in lateral view; (f) internal anatomy of *Sutekhsuchus dowsoni* in lateral view. CCA, cephalic carotid arteries; EC, external choana; EL, endosseous labyrinth; END, endocast; NC, nasal cavity; NLD, nasolacrimal duct; NPD, nasopharyngeal duct; NVC, neurovascular canal; OF, olfactory region; PS, palatine sinus; PTS, paratympanic sinus. Scale bar = 10 cm.

**FIGURE 36:**
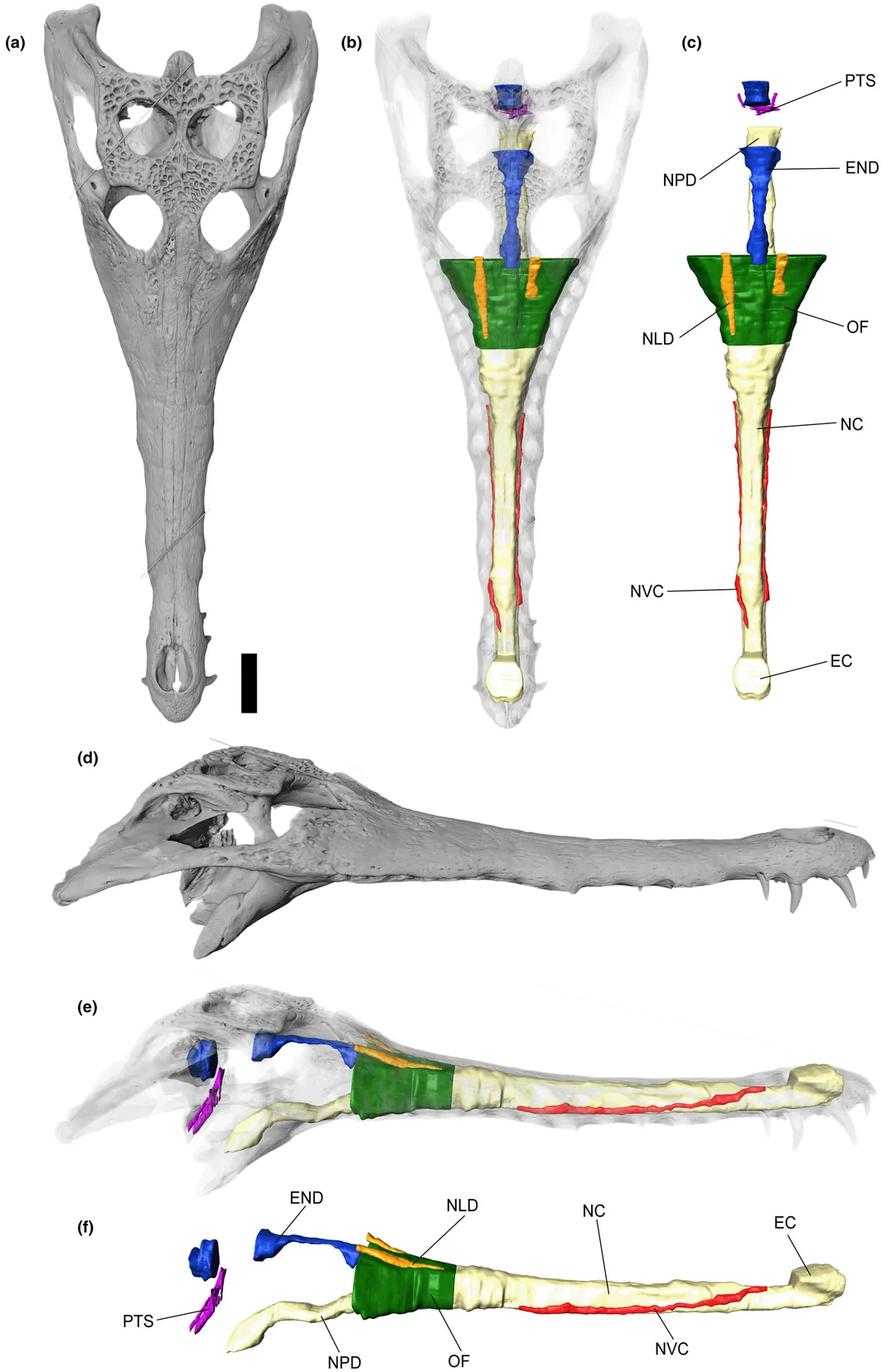
Skull of *Thecachampsa americana* (SMM P8681). (a) skull rendering in dorsal view; (b) skull rendered transparent with internal anatomy visible in dorsal view; (c) internal anatomy of *Thecachampsa americanus* in dorsal view; (d) skull rendering in dorsal view; (e) skull rendered transparent with internal anatomy visible in lateral view; (f) internal anatomy of *Thecachampsa americanus* in lateral view. EC, external choana; END, endocast; NC, nasal cavity; NLD, nasolacrimal duct; NPD, nasopharyngeal duct; NVC, neurovascular canal; OF, olfactory region; PTS, paratympanic sinus. Scale bar = 10 cm.

**FIGURE 37:**
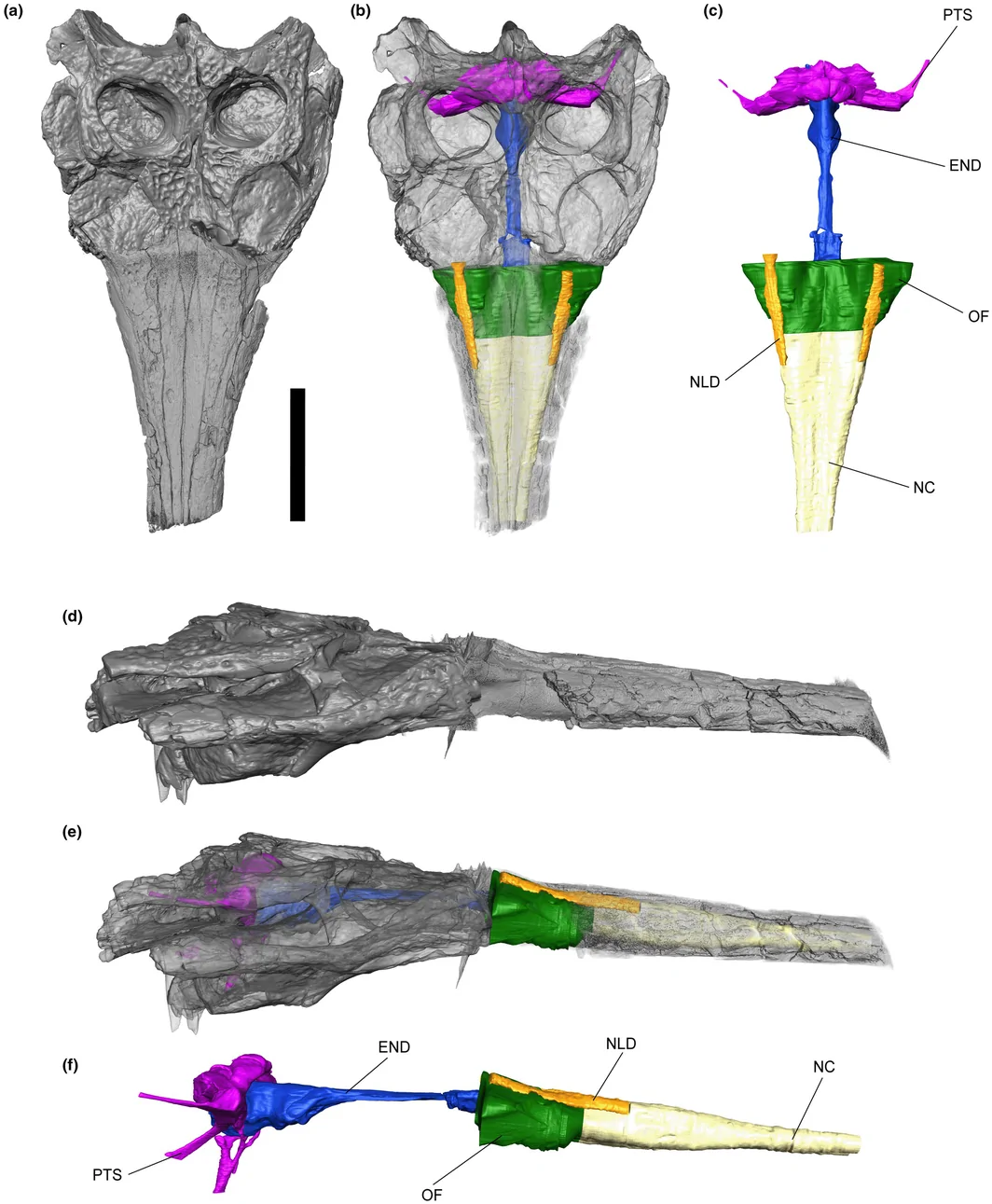
Skull of *Thoracosaurus isorhynchus* (MGL 54101) adapted from Boerman et al. (in review). (a) skull rendering in dorsal view; (b) skull rendered transparent with internal anatomy visible in dorsal view; (c) internal anatomy of *Thorcosaurus isorhynchus* in dorsal view; (d) skull rendering in lateral view; (e) skull rendered transparent with internal anatomy visible in lateral view; (f) internal anatomy of *Thoracosaurus isorhynchus* in lateral view. END, endocast; NC, nasal cavity; NLD, nasolacrimal duct; OF, olfactory region; PTS, paratympanic sinus. Scale bar = 10 cm.

**FIGURE 38:**
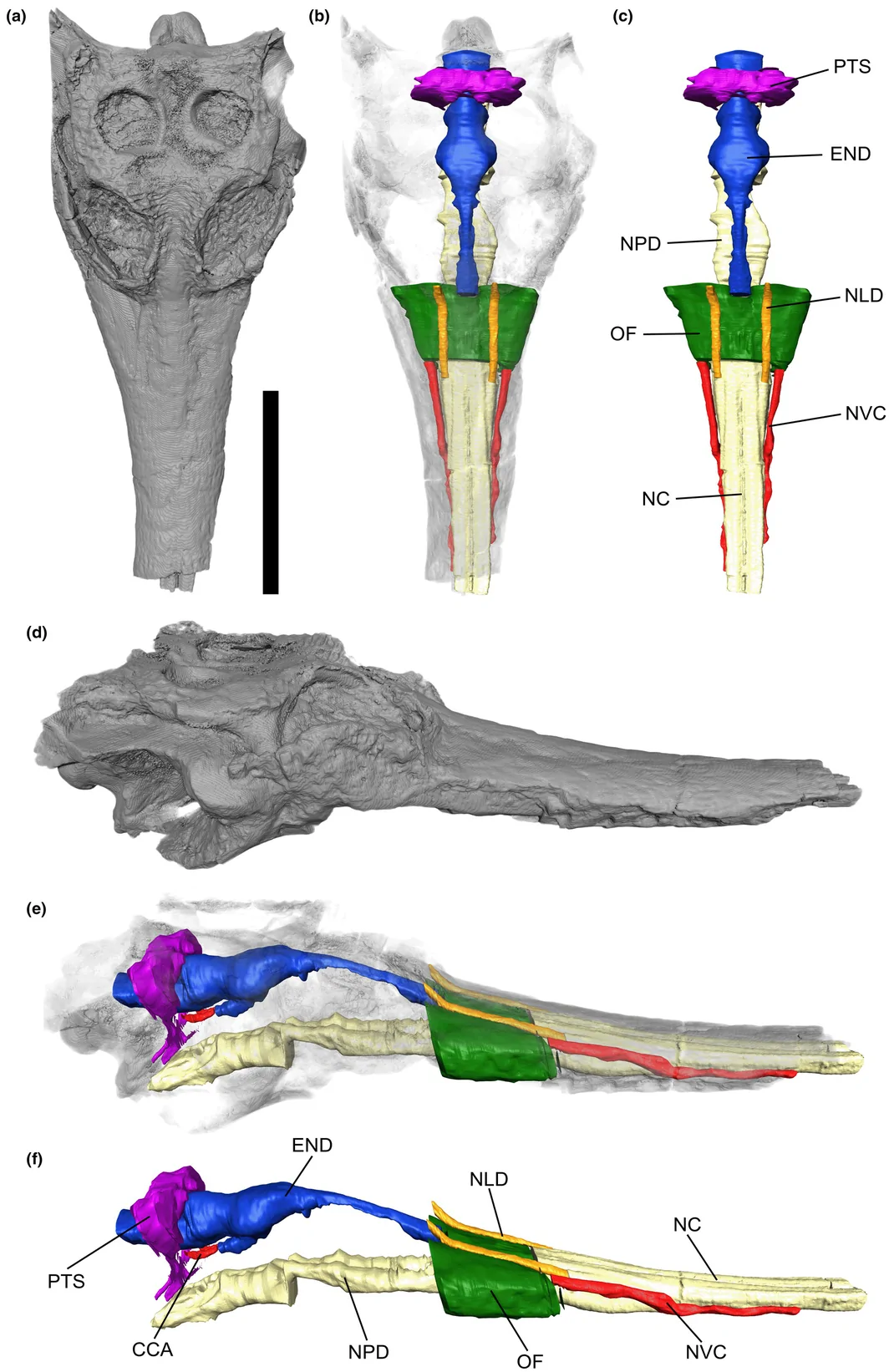
Skull of ‘*Tomistoma’ cairense* (SMNS 10575). (a) skull rendering in dorsal view; (b) skull rendered transparent with internal anatomy visible in dorsal view; (c) internal anatomy of *‘Tomistoma’ cairense* in dorsal view; (d) skull rendering in dorsal view; (e) skull rendered transparent with internal anatomy visible in lateral view; (f) internal anatomy of ‘*Tomistoma’ cairense* in lateral view. CCA, cephalic carotid arteries; END, endocast; NC, nasal cavity; NLD, nasolacrimal duct; NPD, nasopharyngeal duct; NVC, neurovascular canal; OF, olfactory region; PTS, paratympanic sinus. Scale bar = 10 cm.

**FIGURE 39:**
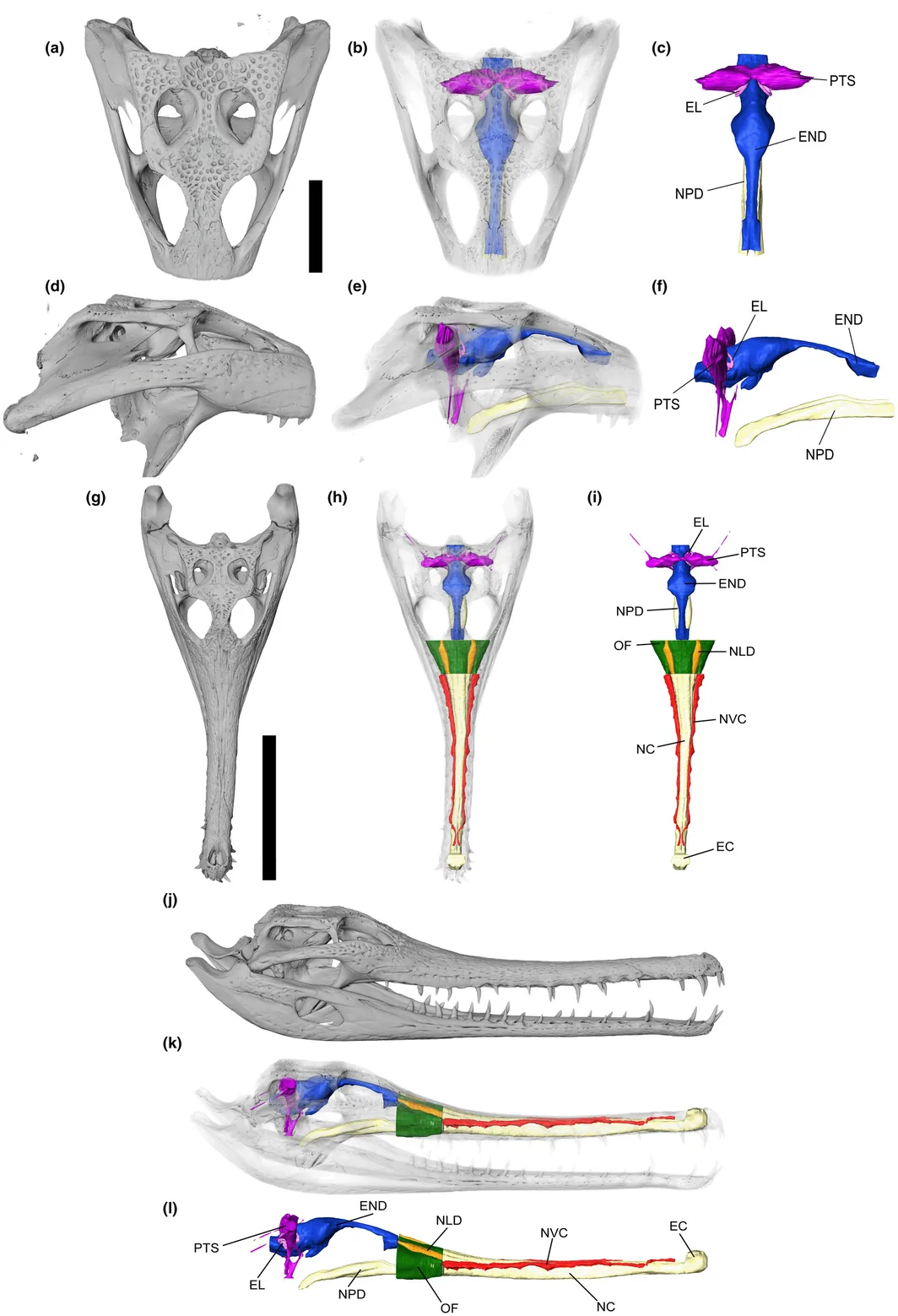
Skull of an adult specimen of *Tomistoma schlegelii* (TMM M6342). (a) skull rendering in dorsal view; (b) skull rendered transparent with internal anatomy visible in dorsal view; (c) internal anatomy of *Tomistoma schlegelii* in dorsal view. (d) skull rendering in lateral view; (e) skull rendered transparent with internal anatomy visible in lateral view; (f) internal anatomy of *Tomistoma schlegelii* in lateral view. Skull of a subadult specimen of *Tomistoma schlegelii* (NHMUK 1893.3.6.14). (g) skull rendering in dorsal view; (h) skull rendered transparent with internal anatomy visible in dorsal view; (i) internal anatomy of *Tomistoma schlegelii* in dorsal view; (j) skull rendering in lateral view; (k) skull rendered transparent with internal anatomy visible in lateral view; (l) internal anatomy of *Tomistoma schlegelii* in lateral view. EC, external choana; EL, endosseous labyrinth; END, endocast; NC, nasal cavity; NLD, nasolacrimal duct; NPD, nasopharyngeal duct; NVC, neurovascular canal; OF, olfactory region; PTS, paratympanic sinus. Scale bars = 5 cm (a); 10 cm (g).

### 3.2 Endosseous labyrinth

The endosseous labyrinth is the bony outer wall of the inner ear. It has been associated with hearing, balance and other locomotory behaviours across terrestrial tetrapods (e.g. Brusatte et al., 2016; Cuthbertson et al., 2015; Georgi, 2008; Rogers, 2005; Spoor, 2003; Spoor et al., 1994, 2002; Witmer et al., 2008). The endosseous labyrinth consists of an internal membranous layer that is surrounded by a bony labyrinth. The latter is differentiated into two distinct sections: (1) the vestibular apparatus, which is formed of the vestibule, as well as the anterior, posterior and lateral semicircular canals, and is associated with equilibrium and spatial position; and (2) the lagena (Barrios et al., 2023) or cochlear duct (Pierce et al., 2017; Ristevski, 2022), which projects posteroventrally and is responsible for sound processing (Georgi, 2008).

The vestibule forms the base of the vestibular apparatus (Perrichon et al., 2024; Ristevski, 2022). The common crus extends dorsally from the vestibule, connecting the anterior and posterior semicircular canals to one another at their most dorsal tips (Ristevski, 2022). In some species (see below), the lateral semicircular canal is characterised by a ‘swelling’ in the anteriormost part of the vestibule, known as the ampulla (Perrichon et al., 2024; Ristevski, 2022). The vestibular apparatus is also characterised by the fenestra ovalis, situated on the lateral side of each endosseous labyrinth, ventral to the lateral semicircular canal, as well as the fenestra pseudorotunda, which is located posteroventral to the fenestra ovalis (Ristevski, 2022).

The endosseous labyrinth could not be reconstructed for any of the non‐crocodylian eusuchians evaluated herein (Puértolas‐Pascual et al., 2022; Serrano‐Martínez et al., 2021). It could be partially segmented for *Paralligator*, but it is largely incomplete and anatomically uninformative (Kuzmin et al., 2025). The vestibular apparatus of crocodylians is typical of those of other crocodylomorphs, and archosaurs more broadly (Bronzati et al., 2021; Brusatte et al., 2016), in which the area of the anterior semicircular canal is larger than that of the posterior semicircular canal. The separation between the cochlear duct of the endosseous labyrinth and middle ear of the paratympanic sinus is indistinguishable in most extant crocodylians, with some authors reconstructing the cochlear duct to expand into the middle ear (Schwab et al., 2020). For consistency in this study, we reconstruct the termination of the cochlear duct to be where it meets the internal surface of the parabasisphenoid (Kuzmin et al., 2021).

In Alligatoroidea, the endosseous labyrinth is relatively equidimensional. The area of the anterior semicircular canal (ASC) is more than three times that of the posterior semicircular canal (PSC), with the most extreme morphology observed in *Alligator mississippiensis*, in which the ASC is more than six times the size of the PSC (Figure 42; Table 2). The ampulla on the lateral semicircular canal and the fenestra ovalis can be identified in all alligatoroids; however, the fenestra pseudorotunda could not be reconstructed from our CT data (Figure 42). *Alligator sinensis* possesses the largest ampulla in alligatoroids, whereas the ampulla in *Caiman crocodilus* is reduced (Figure 42).

The endosseous labyrinth is also relatively equidmensional in Crocodyloidea. In most crocodyloids, the ASC to PSC ratio is approximately 2–3 (Table 2). However, *Mecistops cataphractus* and *Osteolaemus tetrapsis* are characterized by ratios of five and six, respectively. As in alligatoroids, the ampulla is present in all crocodyloids, and reflects the morphology seen in caimanines (Figure 42). The fenestra ovalis can be identified in all crocodyloids; however, the fenestra pseudorotunda could only be identified in *Trilophosuchus rackhami* and *Voay robustus* (Figure 42; Ristevski, 2022; Perrichon et al., 2024).

The ASC to PSC ratio is approximately 2–3 in Gavialoidea (Table 2). In several gavialoids, such as *Argochampsa krebsi*, *Eosuchus lerichei*, *Piscogavialis jugaliperforatus* and *Portugalosuchus azenhae*, the morphology of the semicircular canals is more dorsoventrally compressed than those of other crocodylians. The semicircular canals of some fossil gavialoids, namely *Eosuchus lerichei*, *Piscogavialis jugaliperforatus* and *Thoracosaurus isorhynchus* (Boerman et al., in review), are also thickened relative to those of other crocodylians. These species are also characterized by a larger ampulla than other gavialoids, similar to the morphology seen in *Alligator sinensis* (Figure 42). The fenestra ovalis is identifiable in all gavialoids; however, the fenestra pseudorotunda could not be identified from our CT scan data (Figure 42).

### 3.3 Paratympanic sinus

The paratympanic sinus surrounds the encephalic endocast. This sinus functions to enhance the acoustic capabilities of the middle ear (Dufeau & Witmer, 2015; Witmer & Ridgely, 2008) and has been well studied in extant crocodylians (Perrichon et al., 2023). The paratympanic sinus consists of three expansions, all of which are confluent with one another: the median pharyngeal sinus, the pharyngotympanic sinus system, and the intertympanic sinus system (Perrichon et al., 2024).

The median pharyngeal sinus passes through the basisphenoid and comprises the median pharyngeal canal and the basisphenoid and basioccipital recesses (Perrichon et al., 2024). At its most dorsal part, the median pharyngeal canal separates into two components, with the anterior component forming the basisphenoid recess and the posterior component forming the basioccipital recess. The confluence of these two components is referred to as the pharyngeal intersection (Perrichon et al., 2024). The basisphenoid recess, which inflates the medial part of the basisphenoid, connects to the *recessus epitubaricus*. The latter is an anterior extension of the pharyngotympanic sinus, resulting from a constriction formed by the internal carotid arteries and trigeminal ganglion (Dufeau & Witmer, 2015; Kuzmin et al., 2021).

The basioccipital recess connects the medial pharyngeal canal with the pharyngotympanic sinus system. The latter comprises a rhomboidal recess, which is confluent with the basioccipital recess, the pharyngotympanic recess (=middle ear cavity) and pharyngotympanic tubes. The pharyngotympanic tubes, which connect to the rhomboidal recess ventrally, pass between the basisphenoid and the basioccipital, and exit the braincase through the pharyngotympanic foramina (Perrichon et al., 2024). The pharyngotympanic recess connects to the rhomboidal recess dorsally and is the most voluminous part of the pharyngotympanic sinus system (Perrichon et al., 2024).

The intertympanic sinus, which is mostly confluent with the pharyngotympanic sinus system, is positioned dorsal to the hindbrain of the endocast. It consists of several recesses, the number and morphology of which differ throughout Crocodylia (see Perrichon et al., 2024). Despite the paratympanic sinus as a whole being well studied in extant crocodylians, relatively little is known about this feature in extinct crocodylomorphs (though see Fernández et al., 2011; Pierce et al., 2017). The paratympanic sinus is often only partially segmented in fossil species, due to the difficulty in distinguishing the sinus in low‐contrast CT scans (see Serrano‐Martínez et al., 2021).

The paratympanic sinus could not be reconstructed in *Hylaeochampsa*, but it is partially preserved in *Agaresuchus* and *Arenysuchus*, and well preserved in *Paralligator* (Kuzmin et al., 2025). Taphonomic lateral compression in the skull of *Agaresuchus* means that the separation between the basisphenoid and pharyngeal sinus is indistinguishable (Serrano‐Martínez et al., 2021). The median pharyngeal canal is preserved in *Agaresuchus* and *Paralligator*, with anterior and posterior processes forming the basisphenoid recess and basioccipital recess, respectively, in both species (Serrano‐Martínez et al., 2021: fig. 3; Kuzmin et al., 2025: fig. 17). From the basisphenoid recess, *Paralligator* possesses an expansive *recessus epitubaricus*, as well as two laterosphenoid recesses (Kuzmin et al., 2025: fig. 17). The pharyngotympanic tubes of both *Agaresuchus* and *Paralligator* are mediolaterally wider than dorsoventrally tall (Serrano‐Martínez et al., 2021: fig. 3; Kuzmin et al., 2025: fig. 17). The pharyngotympanic sinus of *Agaresuchus* appears to be dorsoventrally reduced relative to that of *Arenysuchus* and *Paralligator*; however, this is most likely a product of only partial reconstruction of this sinus in *Agaresuchus*, with segmentation in this region difficult as a result of low‐contrast in the CT scans (Serrano‐Martínez et al., 2021: fig. 3). The latter problem also impacts the segmentation of the intertympanic sinus in *Agaresuchus* and *Arenysuchus*, although it is possible to discern that this is anteroposteriorly short in both species (Serrano‐Martínez et al., 2021: figs 3 and 4; Puértolas‐Pascual et al., 2022: fig. 3). In *Paralligator*, the intertympanic sinus is anteroposteriorly expanded and characterized by flat, paired parietal recesses (Kuzmin et al., 2025: fig. 17).

The paratympanic sinus of members of Alligatoroidea is anteroposteriorly expansive. As in *Agaresuchus* and *Paralligator*, the medial pharyngeal canal in Alligatoroidea possesses both anterior and posterior processes; however, most alligatoroids lack a well‐developed basisphenoid recess, except for *Stangerochampsa*, which possesses an expansive *recessus epitubaricus* and laterosphenoid recesses (Donzé et al., 2026; Figure 12). Both *Alligator mississippiensis* and *Diplocynodon tormis* possess an anterior recess, which is a small recess that extends anteriorly from the basisphenoid recess (Serrano‐Martínez et al., 2019: fig. 4; Figure 3). In the most dorsal part of the medial pharyngeal canal, where the canal splits into anterior and posterior processes, Alligatoroidea lacks a depression, whereas this can be observed in lateral view in several other crocodylians (see below; Figures 3, 4, 5, 6, 7, 8, 9, 10 and 12). The pharyngotympanic sinus system of Alligatoroidea is the most anteroposteriorly expansive region of the paratympanic sinus, as seen in both dorsal and lateral views (Figures 3, 4, 5, 6, 7, 8, 9, 10 and 12). The pharyngotympanic tubes are dorsoventrally shorter than mediolaterally wide in Alligatoroidea, as is also the case in non‐crocodylian eusuchians (Figures 4, 5, 6, 7, 8, 9, 10 and 12). Alligatoroids are characterized by three pairs of parietal openings that link the parietal recesses with the intertympanic sinus, whereas other crocodylians only have two pairs (Perrichon et al., 2023). *Alligator*, *Diplocynodon, Leidyosuchus* and *Stangerochampsa* possess a verticalized parietal recess (Serrano‐Martínez et al., 2019: fig. 4; Donzé et al., 2026; Figures 3, 4, 9, 10 and 12), in which the most dorsal portion of the intertympanic sinus is convex in posterior view, whereas this process is flat in *Arambourgia gaudryi* (Conedera et al., 2023; Figure 5) and other caimanines (Perrichon et al., 2023; Figures 6, 7, 8). With the exception of *Caiman latirostris* (Figure 7), the parietal recesses converge in all alligatoroids. They also communicate with the prootic in most alligatoroids, although this is not the case in *Arambourgia* and *Caiman latirostris* (Figures 5 and 7).

The paratympanic sinus of members of Crocodyloidea shows greater morphological variation than that of alligatoroids (Figures 13, 14, 15, 16, 17, 18, 19, 20, 21, 22, 23, 24). As in Alligatoroidea, the most dorsal part of the medial pharyngeal canal possesses anterior and posterior processes to form the basisphenoid and basioccipital recesses. The degree of development of the basisphenoid recess varies throughout Crocodyloidea. *Crocodylus acutus* shows the most reduced recess amongst the clade, in which it is a thin anterior recess from the medial pharyngeal canal, with a small dorsal inflection at its most anterior point (Figure 13f). The basisphenoid recess expands at its most anterior point into the *recessus epitubaricus* in *Crocodylus halli* (Figure 14), *Crocodylus moreletti* (Figure 15), *Crocodylus niloticus* (Figure 16), *Crocodylus novaeguineae* (Figure 17), *Crocodylus rhombifer* (Figure 19), *Crocodylus siamensis* (Figure 20), *Mecistops cataphractus* (Figure 21), *Osteolaemus tetrapsis* (Figure 22), *Paludirex vincenti* (Ristevski et al., 2020), *Trilophosuchus rackhami* (Ristevski, 2022; Figure 23) and *Voay robustus* (Perrichon et al., 2024; Figure 24). Amongst crocodyloids, *Osteolaemus tetrapsis*, *Trilophosuchus rackhami* and *Voay robustus* are characterized by the most developed basisphenoid recesses (Perrichon et al., 2024; Ristevski, 2022), similar to the condition in *Paralligator* and the alligatoroid *Stangerochampsa* (Figures 22, 23, 24). The basisphenoid recess of *Osteolaemus tetrapsis* and *Voay robustus* has a small, anterior extension (=anterior recess; Perrichon et al., 2024). In lateral view, the medial pharyngeal canal intersection is characterised by a narrow depression in all crocodyloids (Figures 13, 14, 15, 16, 17, 18, 19, 20, 21, 22, 23, 24).

The pharyngotympanic sinus system of Crocodyloidea also varies in morphology (Figures 13, 14, 15, 16, 17, 18, 19, 20, 21, 22, 23, 24). Relative to alligatoroids, the pharyngotympanic sinus system is anteroposteriorly shorter in *Crocodylus acutus* (Figure 13), *Crocodylus halli* (Figure 14), *Crocodylus moreletii* (Figure 15), *Crocodylus novaeguineae* (Figure 17), *Crocodylus siamensis* (Figure 20) and *Mecistops cataphractus* (Figure 21). By contrast, the anteroposterior length is more comparable to that of alligatoroids in *Crocodylus niloticus* (Figure 16), *Crocodylus palustris* (Figure 18) and *Crocodylus rhombifer* (Figure 19), with *Osteolaemus tetrapsis* (Figure 22), *Trilophosuchus rackhami* (Ristevski, 2022; Figure 23) and *Voay robustus* (Perrichon et al., 2024; Figure 24) characterized by an anteroposteriorly expansive pharyngotympanic sinus, similar in extent to that of *Paralligator* (Kuzmin et al., 2025). At the most ventral part of the pharyngotympanic sinus, most crocodyloids species are characterized by pharyngotympanic tubes that are dorsoventrally taller than mediolaterally wide, contrasting with those of Alligatoroidea and non‐crocodylian eusuchians. Within Crocodyloidea, *Crocodylus niloticus* is characterized by the dorsoventrally tallest pharyngotympanic tubes relative to mediolateral width, with those of *Crocodylus novaeguineae* the most equidimensional.

In the intertympanic sinus, the parietal recesses of members of Crocodyloidea do not communicate with the prootic. The parietal recesses are separated from each other in *Crocodylus acutus* (Figure 13), *Crocodylus halli* (Figure 14), *Crocodylus palustris* (Figure 18), *Mecistops cataphractus* (Figure 21), *Osteolaemus tetrapsis* (Figure 22) and *Voay robustus* (Perrichon et al., 2024; Figure 24). By contrast, the parietal recesses converge in *Crocodylus moreletti* (Figure 15), *Crocodylus niloticus* (Figure 16), *Crocodylus novaeguineae* (Figure 17), *Crocodylus rhombifer* (Figure 19), *Crocodylus siamensis* (Figure 20) and *Trilophosuchus rackhami* (Ristevski, 2022; Figure 23). The parietal recess is verticalized in *Crocodylus moreletii* (Figure 15), *Crocodylus novaeguineae* (Figure 17), *Crocodylus palustris* (Figure 18) and *Crocodylus siamensis* (Figure 20). Both *Osteolaemus* and *Voay* possess an expanded and verticalized otoccipital recess, and a flat parietal recess (Perrichon et al., 2024; Figures 22 and 24).

Due to the poor quality of the scan and/or lack of preservation, the paratympanic sinus could not be fully reconstructed in several extinct gavialoids, namely *Argochampsa krebsi* (Figure 25), *Eogavialis gavialoides* (Figure 26), *Gavialosuchus eggenburgensis* (Figure 30), *Kentisuchus spenceri* (Figure 32) and *Thecachampsa americana* (Figure 36). In *Gryposuchus neogaeus*, the paratympanic sinus has been segmented; however, there is uncertainty over the presence of specific features, such as lateroventral canals, due to the quality of the CT scan (Bona et al., 2015: fig. 9). The paratympanic sinus of members of Gavialoidea is mostly consistent in terms of overall shape (see Figures 27, 28, 29, 31 and 33, 34, 35, 36, 37, 38, 39). It is anteroposteriorly shorter than in other crocodylians. The anterior process of the medial pharyngeal canal is poorly developed and is seemingly absent in both *Piscogavialis jugaliperforatus* (Figure 33) and *Portugalosuchus azenhae* (Figure 34); however, this could be due to poor preservation in this region in specimens of these two taxa. The *recessus epitubaricus* is preserved in several gavialoids, consisting of *Eosuchus lerichei* (Figure 27), *Eothoracosaurus mississippiensis* (Figure 28), *Gavialis gangeticus* (Figure 29), *Thoracosaurus isorhynchus* (Figure 37) and *Tomistoma schlegelii* (Figure 39); of these taxa, the *recessus epitubaricus* connects to the pharyngotympanic sinus system only in the latter three species (see Boerman et al., in review; Burke et al., 2025). However, the seemingly absent connection in *Eosuchus* and *Eothoracosaurus* is likely a preservational artefact of those specimens. The medial pharyngeal canal intersection possesses a wide, ‘U’‐shaped depression in all gavialoids, except for *Eothoracosaurus mississippiensis* (Figure 28). All gavialoids possess anteroposteriorly short, mediolaterally wide pharyngotympanic tubes at the most ventral part of the pharyngotympanic sinus system. Relative to that of other crocodylians, the pharyngotympanic sinus is transversely compressed in all gavialoids. By contrast with other gavialoids, the pharyngotympanic sinus system of ‘*Tomistoma*’ *cairense* is anteroposteriorly expansive (Figure 38), similar to the morphology in alligatoroids and some crocodyloids.

The intertympanic sinus varies in morphology across Gavialoidea (see Figures 27, 28, 29, 31, 33, 34, 35 and 37, 38, 39). This sinus is flat in *Eosuchus lerichei* (Figure 29), *Eothoracosaurus mississippiensis* (Figure 28), *Gryposuchus neogaeus* (Bona et al., 2015: fig. 9), *Piscogavialis jugaliperforatus* (Figure 33), *Portugalosuchus azenhae* (Figure 34), *Sutekhsuchus dowsoni* (Figure 35) and *Tomistoma schlegelii* (Figure 39). In both *Gavialis gangeticus* (Figure 29) and *Thoracosaurus isorhynchus* (Figure 37), the intertympanic sinus possesses inflated parietal recesses, and ‘*Tomistoma*’ *cairense* (Figure 38) possesses two parietal recesses that are not inflated. Additionally, the intertympanic sinus reduces in anteroposterior length medially in *Eosuchus*, *Gryposuchus*, *Piscogavialis*, *Portugalosuchus, Sutekhsuchus* and *Tomistoma schlegelii*.

### 3.4 Nasal cavity and associated structures

The nasal cavity extends to the anterior end of the rostrum, where it terminates in the external naris (=external choana). Neurovascular canals run parallel to the nasal cavity. Some eusuchian species possess additional snout sinuses, comprising the antorbital sinus, postvestibular sinus, maxillary sinus and palatine sinus, which are lined with the respiratory epithelium. Anterior to the encephalic endocast and orbits, the nasal passageway in crocodylomorphs expands mediolaterally to form the olfactory region, the dorsal region of the cavum nasi proprium (Parsons, 1970). Anteriorly, the olfactory region narrows in mediolateral width. The olfactory region is separated from the posteroventral continuation of the nasal passageway (= nasopharyngeal ducts) and from the respiratory region of the nasal cavity by a bony chamber (Pierce et al., 2017). Crocodylians possess nasolacrimal ducts, which are paired ducts on the dorsal surface of the olfactory region. Similar ducts have been identified within the lacrimal of the early‐diverging crocodylomorph *Junggarsuchus sloani* (Ruebenstahl et al., 2022: fig. 7), the dyrosaurid *Rhabdognathus aslerensis* (Erb & Turner, 2021: fig. 3) and the notosuchian *Barreirosuchus franciscoi* (Fachini et al., 2025: fig. 13); however, the position of these ducts differs from that of crocodylians, and it is unclear whether this is a homologous feature.

In the non‐crocodylian eusuchians included in this study, the nasal cavity could only be partially reconstructed in *Agaresuchus* (Serrano‐Martínez et al., 2021: fig. 3), *Arenysuchus* (Puértolas‐Pascual et al., 2022: fig. 3) and *Hylaeochampsa* (Figure 2), with only the olfactory region and nasopharyngeal ducts possible to segment in the latter two species. The external choana of *Agaresuchus* is comparable in size to those seen in Alligatoroidea and Crocodyloidea (see below). *Agaresuchus* is also characterized by two neurovascular canals (referred to as ‘dorsal alveolar canals’ in Serrano‐Martínez et al., 2021), one on either side of the nasal cavity, as in all crocodylians. *Agaresuchus* also possesses a ‘paranasal sinus system’ (Serrano‐Martínez et al., 2021), which is located ventrolateral to the nasal cavity. Based on the shape and extent of this sinus, it appears to be equivalent (and possibly homologous) to the postvestibular sinus and maxillary sinus seen in several species of alligatoroids and crocodyloids, without a clear separation between the two sinuses in *Agaresuchus*. The olfactory region is bulbous and its lateral margins curve anteriorly in *Agaresuchus* and *Arenysuchus*, resulting in a round anterior margin (Serrano‐Martínez et al., 2021: fig. 3; Puértolas‐Pascual et al., 2022: fig. 3). In *Hylaeochampsa*, the lateral margins are straighter, resulting in a straight anterior margin (Figure 2).

In Alligatoroidea, the nasal cavity occupies between 29% and 39% of the transverse width of the rostrum, measured at the 4th maxillary alveolus (Table 2). Both extant species of *Alligator*, as well as *Arambourgia*, possess an anteroposteriorly elongate septum that divides the external choana (Figures 3, 4, 5). Anterior to the olfactory region, and lateral to the nasal cavity, extant alligatoroids possess an antorbital and postvestibular sinus, separated from one another by a bony chamber. *Caiman latrirostris* (Figure 7) and *Caiman yacare* (Figure 8) possess an additional series of small sinuses, which are herein referred to as the antorbital sinus. The presence of a palatine sinus in *Alligator sinensis* (Figure 4; Donzé et al., 2026) appears to be an autapomorphic acquisition of this feature within Alligatoroidea. The transverse width of the nasolacrimal ducts of *Alligator* (Figures 3 and 4) and *Paleosuchus palpebrosus* (Figure 11) are greater than a third of their length, resulting in thicker ducts than those seen in other caimanines (Figures 6, 7, 8).

Regions anterior to the endocast could not be reconstructed in *Crocodylus acutus* (Figure 13), *Crocodylus niloticus* (Figure 16a–f), *Crocodylus palustris* (Figure 18), *Crocodylus siamensis* (Figure 20) and *Osteolaemus tetraspis* (Figure 22) because only the skull tables of the specimens of these species were CT‐scanned (see Perrichon et al., 2023). In Crocodyloidea, the nasal cavity occupies between 26% (*Crocodylus moreletii*; Figure 15) to 49% (*Crocodylus halli*; Figure 14) of the snout width (Table 2). Lateral to the nasal cavity and anterior to the olfactory region, most crocodyloids are characterised by the presence of antorbital and postvestibular sinuses. However, *Crocodylus niloticus* (Figure 16g–l), *Crocodylus novaeguineae* (Figure 17) and *Mecistops cataphractus* (Figure 21) only possess the antorbital sinus. The size of the antorbital sinus remains consistent throughout most crocodyloids, with that of *Crocodylus niloticus* the most anteroposteriorly expanded (Figure 16g–l). The size of the postvestibular sinus shows greater variation amongst crocodyloids that possess it: it extends for the full anteroposterior length of the nasal cavity in *Crocodylus moreletii* (Figure 15) and *Crocodylus rhombifer* (Figure 19), whereas it is shorter and varies in length on either side of the nasal cavity in *Crocodylus halli* (Figure 14). A maxillary sinus is present in *Crocodylus halli* (Figure 14), *Crocodylus niloticus* (Figure 16g–l) and *Crocodylus rhombifer* (Figure 19), which is situated ventral to the nasal cavity and postvestibular sinus. *Crocodylus halli* (Figure 14) is the only crocodyloid to possess a palatine sinus, which is situated ventral to the nasal cavity and posterior to the maxillary sinus. The olfactory region is expansive and bulbous in Crocodyloidea. In *Crocodylus acutus*, *Crocodylus halli*, *Crocodylus palustris* and *Crocodylus rhombifer*, the dorsal surface of the olfactory region is convex (see Figure 40), with a corresponding depression on the internal surface of the prefrontal. Amongst eusuchians, this feature has previously been identified only amongst extinct species of gavialoids (see below) and is absent in Alligatoroidea. The nasolacrimal ducts vary in mediolateral width in crocodyloids: *Crocodylus halli* (Figure 14), *Crocodylus novaeguineae* (Figure 17) and *Crocodylus rhombifer* (Figure 19) have mediolaterally thick ducts, with a transverse width to anteroposterior length ratio of 0.3 or greater, whereas all other crocodyloids display a ratio lower than 0.3.

**FIGURE 40:**
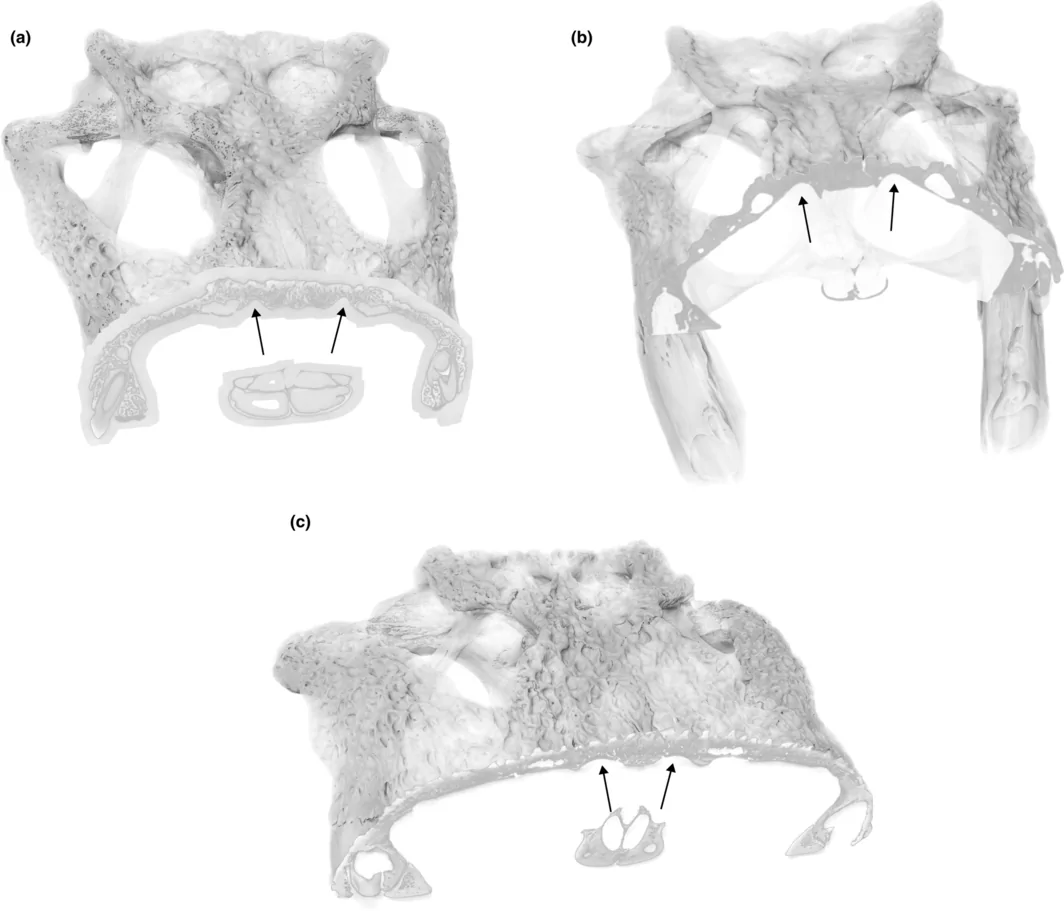
Skulls of (a) *Crocodylus halli*, (b) *Crocodylus rhombifer*, and (c) *Crocodylus palustris* rendered transparent in anterior view, showing a CT slice of the olfactory region, with the anterior section of the snout removed. Arrows indicate the concave depressions in the prefrontal bones.

The nasal cavity could not be reconstructed or fully segmented in several gavialoids, namely *Eogavialis gavialoides* (Figure 26), *Kentisuchus spenceri* (Figure 32), *Piscogavialis jugaliperforatus* (Figure 33), *Thoracosaurus isorhynchus* (Figure 37) and ‘*Tomistoma*’ *cairense* (Figure 38). Unlike some other crocodylians, nearly all gavialoids lack antorbital, postvestibular and palatine sinuses, except for *Gavialis gangeticus*, which possesses an antorbital sinus (Figure 29), and *Sutekhsuchus dowsoni*, which possesses a palatine sinus (Figure 35). Relative to that of other gavialoids (and crocodylians more generally), the olfactory region is small both in anteroposterior length and mediolateral width in *Gavialis gangeticus* (Figure 29) and *Gavialosuchus eggenburgensis* (Figure 30). In several gavialoids, namely *Argochampsa krebsi* (Figure 25; Pligersdorffer et al., 2025), *Eosuchus lerichei* (Figure 27), *Eothoracosaurus mississippiensis* (Figure 28; Boerman et al., in review), *Kentisuchus spenceri* (Figure 32), *Piscogavialis jugaliperforatus* (Figure 33), *Portugalosuchus azenhae* (Figure 34; Burke et al., 2025), *Sutekhsuchus dowsoni* (Figure 35; Pligersdorffer et al., 2025), *Thoracosaurus isorhynchus* (Figure 37; Boerman et al., in review) and ‘*Tomistoma*’ *cairense* (Figure 38), the dorsal surface of the olfactory region is characterized by bulbous, convex expansions, which correspond to depressions on the internal surface on the prefrontal. In most members of Gavialoidea, the nasolacrimal ducts are relatively straight and parallel; however, the lateral margins of these ducts are convex in *Eothoracosaurus mississippiensis* (Figure 28) and *Gavialis gangeticus* (Figure 29). By contrast with Crocodyloidea, the nasolacrimal ducts remain consistent in size in gavialoids.

### 3.5 Nasopharyngeal ducts

Posterior to the olfactory region and ventral to the encephalic endocast, the nasopharyngeal ducts extend from the nasal cavity to the internal choana. In the non‐crocodylian eusuchians *Agaresuchus* and *Arenysuchus*, the nasopharyngeal ducts are bifurcated along their entire length, and the combined mediolateral width of both ducts is less than 75% that of the cerebrum (Puértolas‐Pascual et al., 2022; Serrano‐Martínez et al., 2021).

In Alligatoroidea, the nasopharyngeal ducts remain bifurcated throughout their entire length. In *Caiman latirostris* (Figure 7) and *Paleosuchus palpebrosus* (Figure 11), the ducts retain a consistent dorsoventral height and mediolateral width along their extent. However, in other alligatoroids, the ducts are dorsally expanded posterior to the olfactory region.

In most members of Crocodyloidea, the nasopharyngeal ducts are bifurcated along their entire length and remain consistent in their height and width. However, in *Crocodylus halli* (Figure 14), *Crocodylus moreletii* (Figure 15), *Crocodylus niloticus* (Figure 16g–l), *Crocodylus novaeguineae* (Figure 17) and *Crocodylus rhombifer* (Figure 19), the nasopharyngeal ducts converge anteriorly where they meet the internal choana. *Crocodylus niloticus* and *Crocodylus palustris* each display a dorsal expansion in their nasopharyngeal ducts, similar to that of some alligatoroids (Figures 16g–l and 18).

The nasopharyngeal ducts are bifurcated in all gavialoid species; however, in *Gavialis gangeticus*, the ducts converge anteriorly where they meet the internal choana, matching the morphology seen in some crocodyloids. *Gavialis gangeticus* is also characterized by a pterygoid bulla (Figure 29), a large, egg‐shaped feature positioned lateral to the nasopharyngeal ducts, with no bony separation between them (see Bourke et al., 2021; Burke et al., 2025; Castillo & Gold, 2025). *Gavialis gangeticus* is the only extant crocodylian species to possess this bulla; however, it has been reported in several fossil gavialoids, namely *Gavialis lewisi*, *Gavialis bengawanicus*, *Eogavialis africanum* and *Hanyusuchus sinensis* (Hecht & Malone, 1972; Iijima et al., 2022; Lull, 1944; Martin et al., 2012). *Dollosuchoides densmorei*, *Eogavialis gavialoides*, *Eosuchus lerichei* and *Portugalosuchus azenhae* have all been interpreted to have an ‘early form’ of the bulla, as the nasopharyngeal ducts are laterally expansive relative to the width of the cerebrum in these species (Brochu, 2007; Burke et al., 2025). The lateral expansion of the nasopharyngeal ducts is herein newly observed in *Kentisuchus spenceri* (Figure 32), *Piscogavialis jugaliperforatus* (Figure 33) and ‘*Tomistoma*’ *cairense* (Figure 38).

## 4 DISCUSSION

### 4.1 Is crocodylian endocranial anatomy phylogenetically informative?

The utility of incorporating characters based on endocranial morphology in phylogenetic analyses of crocodylians has been discussed in several recent contributions (Boerman et al., in review; Burke & Mannion, 2023; Donzé et al., 2026; Gold et al., 2014; Perrichon et al., 2023, 2024; Pochat‐Cottilloux et al., 2025). Gold et al. (2014) showed that the inclusion of morphometric data into a combined evidence (morphology + molecular) dataset supported the placement of *Gavialis* within Longirostres when these data emanated from the braincase of crocodylians, whereas *Gavialis* was recovered as a ‘basal’ crocodylian when these data were based solely on Eustachian canal morphology. Perrichon et al. (2024) formulated 25 morphological characters pertaining to the endocranial anatomy of crocodyloids, using *Alligator mississippiensis* as the outgroup taxon. Incorporation of 15 of these characters into an existing phylogenetic data matrix increased resolution in the resulting topology, which was also in closer agreement with molecular‐based topologies than that produced from analyses without endocranial characters (Perrichon et al., 2024). However, many of the excluded endocranial characters seemed to covary with overall skull shape, rather than representing independent, phylogenetically informative morphological variation. Pochat‐Cottilloux et al. (2025) demonstrated that morphological variation in the endosseous labyrinth of crocodylians is primarily due to allometry and that the development of the labyrinth is linked to braincase conformation. Consequently, as is the case with external anatomy, endocranial features should only be characterised for species based on skeletally mature individuals (see also Castillo and Gold (2025) regarding ontogenetic variation in endocranial anatomy). The results of Pochat‐Cottilloux et al. (2025) also caution against the applicability of considering all endosseous labyrinth morphological variation as phylogenetically informative, though one potential caveat is that their dataset was restricted to extant crocodylians, excluding the rich record from fossil taxa.

Here, taking into account the results and cautionary recommendations of these and other studies, we discuss several endocranial features that may be phylogenetically informative for Crocodylia. We utilize our informal supertree to identify potential synapomorphies of the major subclades (Figure 1). Only one identified endocranial feature appears to be synapomorphic for Crocodylia, which is the presence of nasolacrimal ducts, although these might have evolved independently in other crocodylomorph lineages.

Alligatoroidea is characterized by an encephalic endocast that possesses low cephalic and pontine flexure angles in lateral view, resulting in a sigmoidal morphology (Figure 41). The cerebrum has its greatest transverse width at its posterior point in alligatoroids, which are also characterized by an olfactory bulb that is more than half the length of the overall olfactory tract (Figure 41). In the vestibular apparatus of the endosseous labyrinth, the anterior semicircular canal area is over three times the size of the posterior canal in alligatoroids (Figure 42). All alligatoroids possess an anteroposteriorly expanded paratympanic sinus, with a verticalized parietal recess and pharyngotympanic tubes that are mediolaterally wide as opposed to dorsoventrally tall (Figure 43). The anteroposterior expansion of the paratympanic sinus might be ancestral for Crocodylia, as this morphology is also present in the non‐crocodylian eusuchian *Paralligator* (Kuzmin et al., 2025); however, it is not currently possible to assess this feature in eusuchians more closely related to the crocodylian radiation. *Arambourgia* and both extant species of *Alligator* are characterized by an anteroposteriorly elongate septum that divides the external choana, and *Alligator* is further characterized by the presence of antorbital and postvestibular sinuses (Figure 1). The antorbital sinus is also present in the caimanines *Caiman crocodilus*, *Caiman latirostris*, and *Caiman yacare* (Figure 1). Relative to other alligatoroids, the parietal recess in *Caiman* is flattened, suggesting that this might be an autapomorphy of the genus.

**FIGURE 41:**
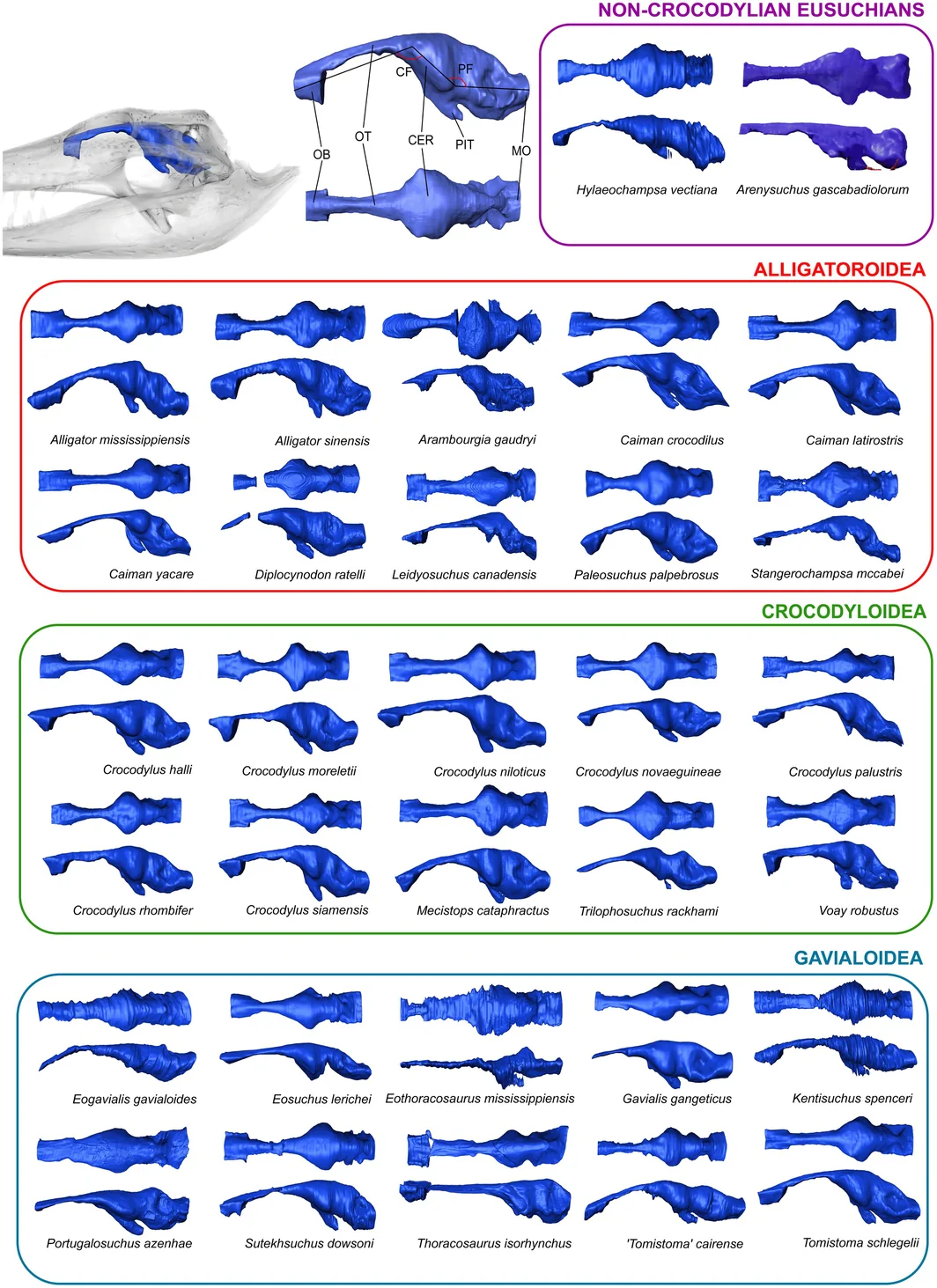
Examples of encephalic endocasts across non‐crocodylian eusuchians and crocodylians in dorsal and lateral view. Skull of *Tomistoma schlegelii* rendered transparent with endocast shown and labelled. Abbreviations. CER, cerebrum; CF, cephalic flexure angle; MO, medulla oblongata; OB, olfactory bulb; OT, olfactory tract; PF, pontine flexure angle; PIT, pituitary fossa.

**FIGURE 42:**
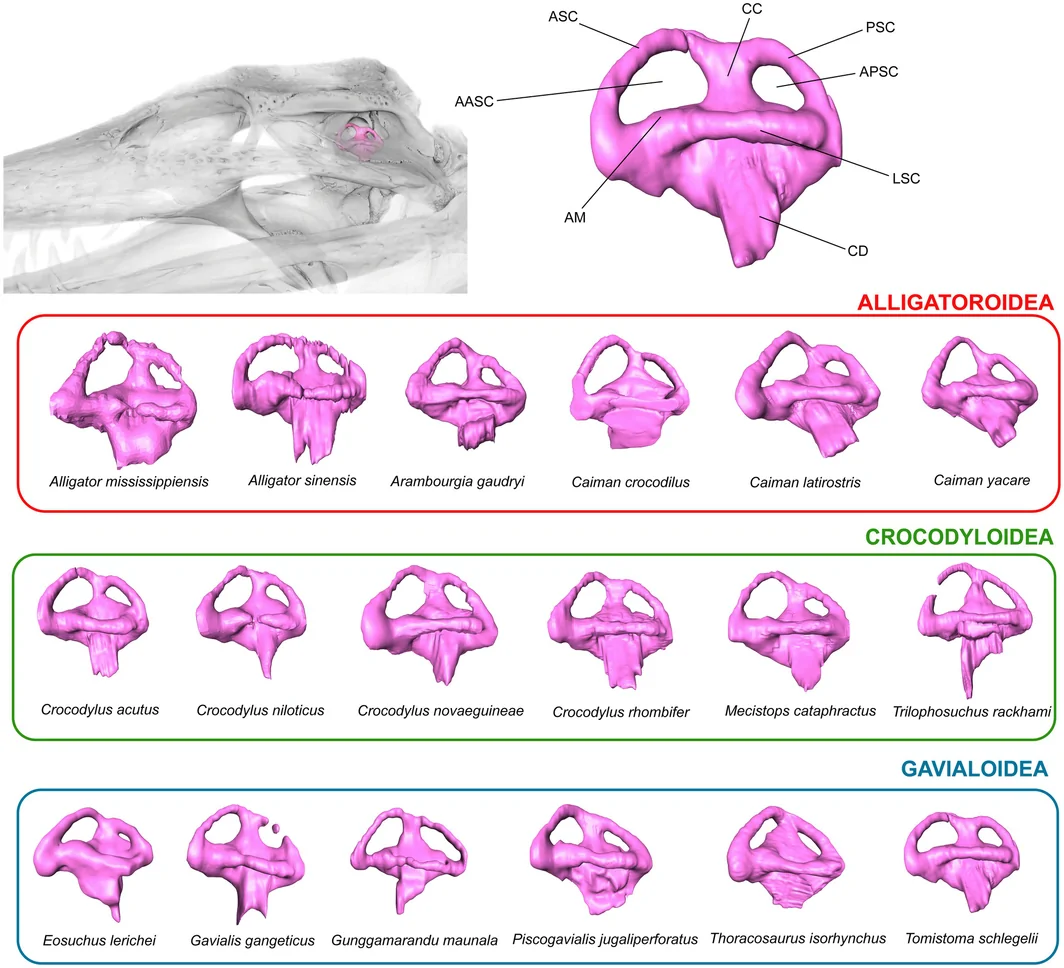
Examples of the endosseous labyrinths of crocodylians in lateral view. Skull of *Tomistoma schlegelii* rendered transparent with endosseous labyrinth shown and labelled. Abbreviations. AASC, anterior semicircular canal area; AM, ampulla; APSC, posterior semicircular canal area; ASC, anterior semicircular canal; CC, common crus; CD, cochlear duct; LSC, lateral semicircular canal; PSC, posterior semicircular canal.

**FIGURE 43:**
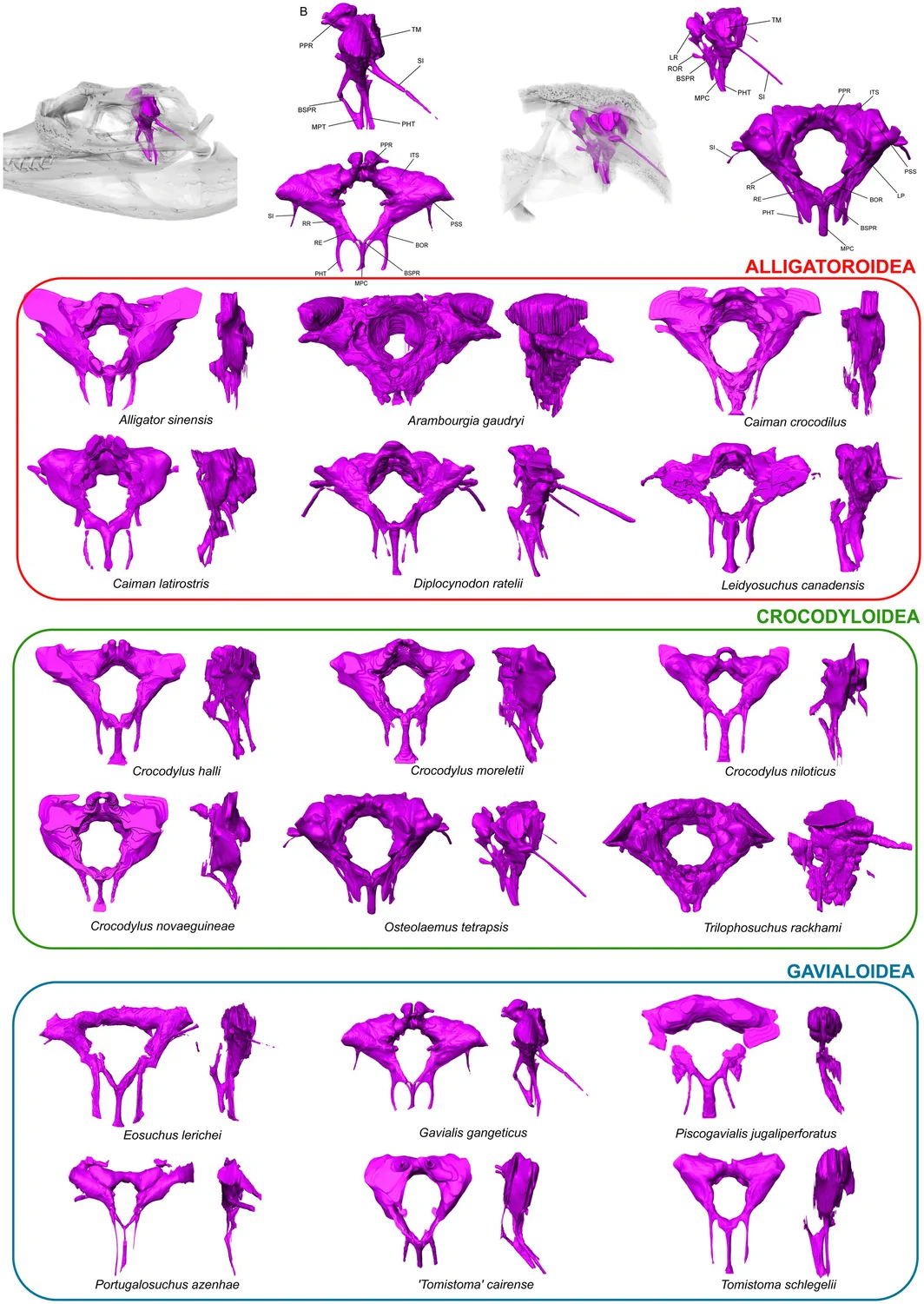
Examples of the paratympanic sinuses of crocodylians in anterior and lateral views. Skulls of *Gavialis gangeticus* and *Osteolameus tetrapsis* rendered transparent with paratympanic sinus shown and labelled. Abbreviations. BSPR, basisphenoid pneumatic recess; BOR, basioccipital recess; ITS, intertympanic sinus; LR, laterosphenoid recess; MPC, medial pharyngeal canal; PHT, pharyngotympanic tubes; PPR, parietal pneumatic recess; PSS, pharyngotympanic sinus system; RE, *recessus epitubaricus*; ROR; rostral pneumatic recess; RR, rhomboidal recess; SI, siphonium; TM, tympanic membrane.

The presence of an anteroposteriorly expansive paratympanic sinus and expansive *recessus epitubaricus* in the crocodyloids *Osteolaemus tetrapsis*, *Trilophosuchus rackhami*, and *Voay robustus* supports the view (see above) that this might be the ancestral condition for Crocodylia, subsequently lost in *Mecistops* and *Crocodylus* (Figures 1 and 43). Additionally, the expansive *recessus epitubaricus* present in *Osteolaemus*, *Trilophosuchus* and *Voay* is potentially synapomorphic for Crocodyloidea, with this lost in *Mecistops* and *Crocodylus* (Figure 43). Crocodyloids are characterized by a paratympanic sinus with dorsoventrally tall pharyngotympanic tubes, antorbital and postvestibular sinuses, and a cerebrum that has its greatest transverse width at its midpoint (Figures 41 and 43). *Crocodylus halli*, *Crocodylus niloticus* and *Crocodylus palustris* revert to a cerebrum wherein the greatest transverse width is at its posterior end (Figure 41), and *Crocodylus niloticus*, *Crocodylus novaeguineae* and *Mecistops cataphractus* show apomorphic loss of the postvestibular sinus (Figure 1). Conversely, the postvestibular sinus is anteroposteriorly expanded in both *Crocodylus morelettii* and *Crocodylus rhombifer* (Figure 1). Both *Crocodylus niloticus* and *Crocodylus palustris* show independent acquisition of an expanded nasopharyngeal duct (Figure 1). The presence of depressions on the internal surface of the prefrontals in several species of *Crocodylus* (*C. acutus*, *C. halli*, *C*. *palustris* and *C. rhombifer*) and their unequivocal absence in others (*C*. *moreletii*, *C*. *niloticus*, *C*. *novaeguineae*) indicates more than one independent acquisition and/or less within the genus (Figures 1 and 40). *Crocodylus halli* and *Crocodylus niloticus* are the only crocodyloids to possess a palatine sinus, suggesting independent acquisition of this feature (Figure 1).

Members of Gavialoidea are characterized by the straightest endocast in lateral view, the greatest transverse width of the cerebrum at its posterior end (Figure 41), an anteroposteriorly short paratympanic sinus, reduced basioccipital recesses in the median pharyngeal canals, a reduced cerebrum width comparative to that of the skull table, the absence of snout sinuses, the presence of depressions in the internal surface of the prefrontals, and an early form of the pterygoid bulla (Figure 1). The latter two features are subsequently lost in *Tomistoma schlegelii*, *Gavialosuchus eggenburgensis* and *Thecachampsa americana*, with the depressions also lost in *Gavialis gangeticus* (Figure 1). The inflation of the pre‐parietal recesses in the paratympanic sinus in *Thoracosaurus isorhynchus* and *Gavialis gangeticus* appears to be independent acquisitions of this feature (Figures 1 and 43). The presence of a postvestibular sinus in *Gavialis gangeticus* was noted by Serrano‐Martínez et al. (2021); however, we disagree with this interpretation and consider it more likely that this recess is associated with alveolar space.

### 4.2 Endocranial anatomical differences between longirostrine crocodylomorph lineages

As is the case in the convergent evolution of the overall longirostrine skull shape of distantly related crocodylomorph lineages, a seemingly associated, generalized endocranial morphology appears to have also evolved in these taxa. This is evident in the ‘simple’ endocranial morphology of thalattosuchians (Pierce et al., 2017), dyrosaurid (Erb & Turner, 2021) and pholidosaurid (Barbini et al., 2026) neosuchians, and gavialoid crocodylians, all of which are characterized by anteroposteriorly ‘straight’ encephalic endocasts, anteroposteriorly compressed pharyngotympanic sinuses, neurovascular canals, and absent or reduced antorbital and postvestibular sinuses. Despite convergence in overall external and internal cranial anatomy, these longirostrine crocodylomorph lineages can nevertheless be distinguished by a number of endocranial features. Whereas endocranial anatomy is well documented for crocodylians and thalattosuchians, our knowledge of that of dyrosaurids and pholidosaurids is largely limited to *Rhabdognathus aslerensis* (Erb & Turner, 2021) and *Pholidosaurus purbeckensis* (Barbini et al., 2026), with CT‐scanned skulls incompletely preserved in both instances.

All crocodylians possess nasolacrimal ducts, which are absent in thalattosuchians (Pierce et al., 2017). These ducts appear to also be present in *Rhabdognathus* (Erb & Turner, 2021: fig. 3), wherein they are much shorter anteroposteriorly than in crocodylians; however, *Pholidosaurus* genuinely lacks these ducts (Barbini et al., 2026). The size, orientation and/or absence of these ducts could therefore aid in the differentiation of longirostrine crocodylians from other crocodylomorphs displaying longirostry.

The encephalic endocasts of thalattosuchians, including *Pelagosaurus typus*, *Metriorhynchus* cf. *westermanni* and *Dakosaurus andiniensis*, possess two dorsally projecting rami (Fernández et al., 2011; Herrera & Vennari, 2015), identified as branches of the dorsal longitudinal venous sinus (Pierce et al., 2017). These are confluent with the paratympanic sinus, and the confluence is absent in all other archosaurs, except for *Rhabdognathus* (Erb & Turner, 2021). A divided dorsal longitudinal venous sinus (Hopson, 1979; Porter et al., 2016) is not present in any eusuchian.

In *Pelagosaurus typus*, *Macrospondylus bollensis* and *Cricosaurus araucanensis*, as well as *Rhabdognathus*, the pituitary fossa is characterised by two anterior projections that represent the orbital arteries (see Pierce et al., 2017: fig. 5; Herrera et al., 2018: fig. 7; Erb & Turner, 2021: fig. 2; Wilberg et al., 2022: fig. 9). These anterior projections are not present in any eusuchian.

The paratympanic sinus is anteroposteriorly reduced in longirostrine crocodylomorphs relative to mesorostrine and brevirostrine species, but the morphology differs between longirostrine clades (Erb & Turner, 2021; Herrera et al., 2018; Pierce et al., 2017; Wilberg et al., 2022). In thalattosuchians, the intertympanic sinus is completely absent, with the dorsal longitudinal venous sinus connecting the pharyngotympanic sinus to the encephalic endocast (see Pierce et al., 2017: fig. 3). Note that in some thalattosuchians, the dorsal longitudinal venous sinus does not appear to meet the pharyngotympanic sinus; however, this could potentially be due to poor preservation in specimens of these species (see Herrera et al., 2018: fig. 7). An intertympanic sinus is present in *Rhabdognathus*, but comprises a thin canal that communicates with the pharyngotympanic sinus (Erb & Turner, 2021: fig. 6). By contrast, the intertympanic sinus in crocodylians is mostly confluent with the pharyngotympanic sinus and is more voluminous than that of *Rhabdognathus*. Whereas both thalattosuchians and *Rhabdognathus* (Erb & Turner, 2021; Herrera et al., 2018; Pierce et al., 2017) have an intertympanic sinus that connects the encephalic endocast to the paratympanic sinus, there is no such contact in crocodylians. Amongst crocodylomorphs, only eusuchians and notosuchians appear to possess pharyngotympanic tubes (see Pierce et al., 2017: fig. 3B; Herrera et al., 2018: fig. 7G; Erb & Turner, 2021: fig. 5; Pochat‐Cottilloux et al., 2022: fig. 3; Wilberg et al., 2022: fig. 10B; Pochat‐Cottilloux et al., 2023: fig. 4).

*Gavialis gangeticus* is the only extant crocodylian to possess a pterygoid bulla (Bourke et al., 2021; Martin & Bellairs, 1977). Evidence for a pterygoid bulla, including a small or ‘early form’ of the bulla is present in several fossil gavialoids, namely *Dollosuchoides densmorei*, *Eogavialis africanum*, *Eogavialis gavialoides*, *Eosuchus lerichei*, *Gavialis bengawanicus*, *Gavialis lewisi*, *Hanyusuchus sinensis*, *Kentisuchus spenceri*, *Piscogavialis jugaliperforatus*, *Portugalosuchus azenhae* and ‘*Tomistoma*’ *cairense* (Lull, 1944; Hecht & Malone, 1972; Brochu, 2007; Martin et al., 2012; Iijima et al., 2022; Burke et al., 2025; this study). Regardless of whether thoracosaurs are gavialoids, this distribution suggests that the pterygoid bulla, or its ‘early form’, is a synapomorphy of Gavialoidea, with gavialoid species that lack this feature (including *Tomistoma schlegelii*) displaying apomorphic loss. If thoracosaurs lie outside of Gavialoidea, then this would suggest an independent origin of the pterygoid bulla in this lineage. No other crocodylomorph clade, including thalattosuchians and dyrosaurids, possesses any form of pterygoid bulla.

Metriorhynchoid thalattosuchians, hypothesized to be fully pelagic, are characterized by an endosseous labyrinth with thickened semicircular canals (Schwab et al., 2021). Thickened semicircular canals also characterize near‐shore teleosauroid thalattosuchians, although not to the same extent as those seen in metriorhynchoids (Schwab et al., 2021). The thickness of the semicircular canals of gavialoids more closely reflects those of teleosaurids than metriorhynchoids; however, several extinct gavialoids, namely *Eosuchus*, *Piscogavialis* and *Thoracosaurus*, are characterized by semicircular canals that are intermediate between extant members of the clade and metriorhynchoids (Burke et al., 2025). By contrast, *Rhabdognathus* possesses an endosseous labyrinth with a thickened vestibular apparatus rather than thickened semicircular canals (Erb & Turner, 2021). As such, semicircular canal thickness potentially has some limited utility in differentiating longirostrine crocodylomorph lineages.

### 4.3 Evidence for ‘nasal salt glands’ in Crocodylomorpha

Extant crocodyloids possess lingual salt glands, which allow them to frequent saltwater and brackish environments (Taplin et al., 1982). These glands are reduced or absent in alligatoroids and gavialoids, with very low secretion rates where present (Taplin et al., 1985). By contrast, there is no evidence for nasal salt glands in any extant crocodylian. The presence of nasal salt glands in extinct crocodylomorphs was first proposed in fully pelagic metriorhynchoid thalattosuchians, with natural casts of salt‐excreting glands reported in a specimen of *Geosaurus araucanensis* (Fernández & Gasparini, 2008). These glands, located anterior to the orbits and prefrontal pillars, can also potentially be identified in the form of depressions on the internal surface of the prefrontal bones (Cowgill et al., 2023). Comparable depressions are present in the extant marine iguana *Amblyrhynchus crisatus*, in which they are osteological correlates of nasal salt glands (Dunson, 1969). As a result, the presence of these depressions in additional metriorhynchoids (see Cowgill et al., 2023), dyrosaurids (Pligersdorffer et al., 2025), pholidosaurids (Barbini et al., 2026) and extinct gavialoids (Burke et al., 2025; Pligersdorffer et al., 2025) has been hypothesized to be an osteological correlate for nasal salt glands in these aquatic crocodylomorph lineages.

Amongst Gavialoidea, these depressions have been identified in *Argochampsa krebsi*, *Eosuchus lerichei*, *Eothoracosaurus mississippiensis*, *Kentisuchus spenceri*, *Piscogavialis jugaliperforatus*, *Portugalosuchus azenhae*, *Sutekhsuchus dowsoni* and *Thoracosaurus isorhynchus* (Boerman et al., in review; Burke et al., 2025; Pligersdorffer et al., 2025). Specimens of all these species come from coastal to shallow marine deposits (Boerman et al., in review). As all these species frequented saltwater environments, it is hypothesized that they must have had adaptations to tolerate saltwater. The widespread presence of these depressions amongst gavialoids supports the hypothesis that saltwater tolerance is the ancestral condition for Gavialoidea (Brochu, 2003; Buffetaut, 1982; Burke et al., 2025; Delfino et al., 2005; Martin et al., 2012). The buccal structure of *Gavialis gangeticus* suggests a secondary reduction (apomorphic loss) for saltwater tolerance (the buccal structure of *Tomistoma schlegelii* has yet to be assessed; Taplin et al., 1985). Based on the topology of Gavialoidea presented in Figure 2, there appears to have been several independent losses (and/or acquisitions) of nasal salt glands, with depressions absent in the extinct subclade formed by *Thecachampsa americana* and *Gavialosuchus eggenburgensis*, in addition to the two extant species.

We herein identify depressions on the internal surface of the prefrontals of crocodyloids for the first time, with their presence documented in *Crocodylus acutus*, *Crocodylus halli*, *Crocodylus palustris* and *Crocodylus rhombifer* (Figure 40). They are unequivocally absent in *Mecistops*, *Crocodylus moreletii*, *Crocodylus niloticus* and *Crocodylus novaeguineae*. Diffusible iodine‐based contrast‐enhanced computed tomography (diceCT) uses iodine as a contrast agent to investigate the relationship between soft and hard tissues in extant species. Due to their high lipid density, glands appear as ‘bright white’ regions in CT scans as they become enriched in iodine (Callahan et al., 2021). A subadult specimen of *Crocodylus acutus*, evaluated using diceCT, has small depressions on the internal surface of the prefrontals; however, these appear to be characterised by airspace rather than housing salt glands, as they do not appear bright white in the CT scan (Blackburn et al., 2024). Airspace appears to also characterise these depressions in a juvenile specimen of *Crocodylus rhombifer* (Melkersson et al., 2024; Figure 44). This suggests that the function of these depressions is to increase capacity for airflow, at least within Crocodyloidea. It therefore potentially casts doubt on their hypothesised function for housing nasal salt glands in gavialoids (as well as dyrosaurids and pholidosaurids). However, the absence of these depressions in several crocodyloids means they are not necessarily ancestral for Crocodyloidea, and thus, their presence in Gavialoidea might be an independent acquisition, rather than a synapomorphy of Longirostres. Given that the depressions, regardless of their function, are convergently present in several crocodylomorph lineages, it remains possible that their function in gavialoids was convergent with that of metriorhynchoid thalattosuchians, rather than crocodyloids.

**FIGURE 44:**
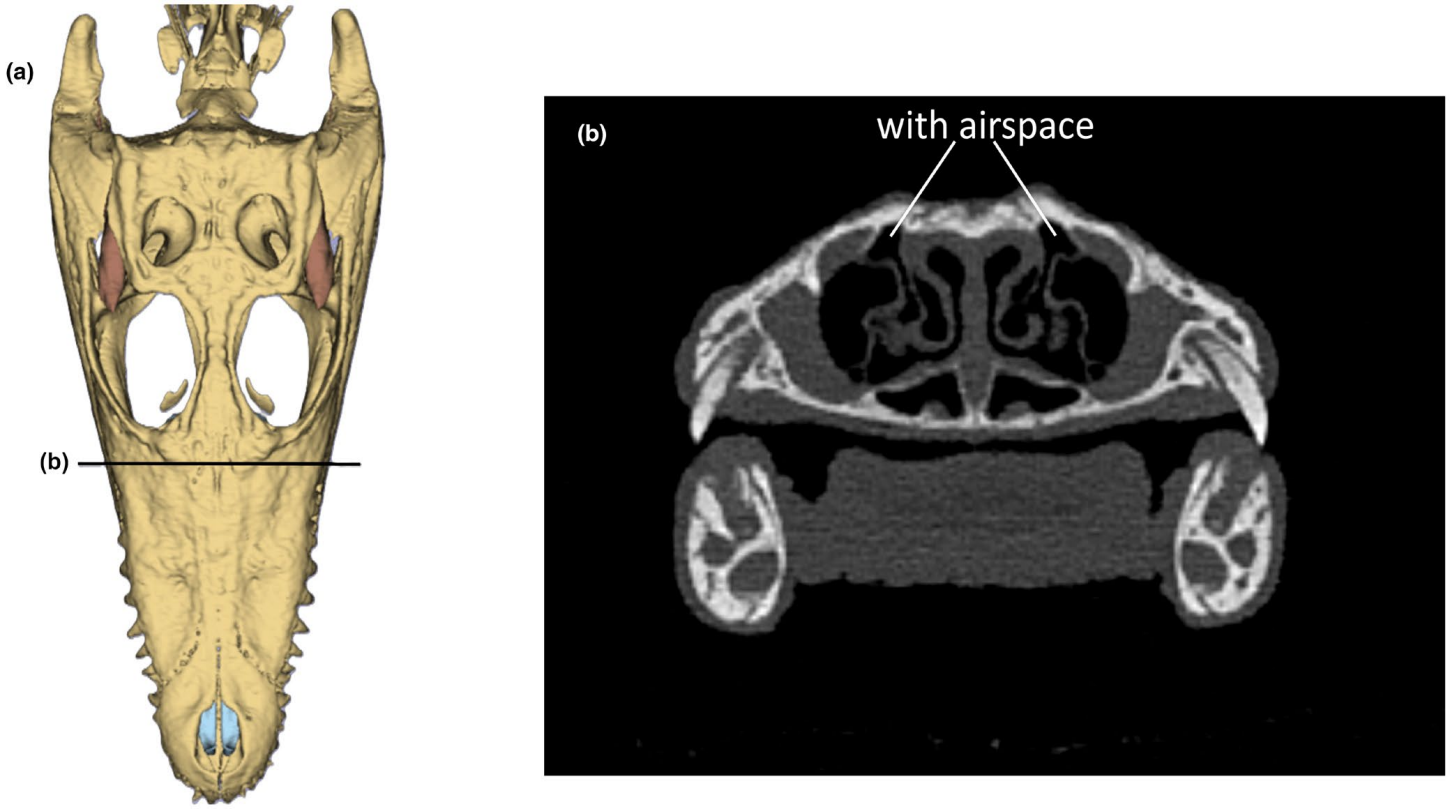
(a) Skull rendering of a juvenile *Crocodylus rhombifer* (adapted from Melkersson et al., 2024) and (b) CT slice showing the olfactory region of *Crocodylus rhombifer*.

## 5 CONCLUSIONS

We present an extensive overview of morphological variation in endocranial anatomy of crocodylians, highlighting a number of features that appear to have a phylogenetic signal between and within the major subclades: Alligatoroidea, Crocodyloidea and Gavialoidea. Although there is convergence in endocranial anatomy between distantly related longirostrine crocodylomorph lineages, especially gavialoids and thalattosuchians, we identify a number of differences that facilitate their differentiation. For the first time, we identify depressions on the internal surface of the prefrontals in crocodyloids, which are characterised by airspace. These have previously been interpreted as osteological correlates for nasal salt glands in extinct gavialoids based on their presence in fully marine thalattosuchians. Consequently, their hypothesized function in gavialoids remains highly uncertain.

## Data Availability

The data that supports the findings of this study are available on MorphoSource: https://www.morphosource.org/projects/000862041?locale=en with 3D models associated with the scans from Perrichon et al. (2023) subsequently uploaded to MorphoMuseum.
